# KLRG1-expressing CD8+ T cells are exhausted and polyfunctional in patients with chronic hepatitis B

**DOI:** 10.1371/journal.pone.0303945

**Published:** 2024-05-22

**Authors:** Li Wang, Fangli Liao, Liping Yang, Linshan Jiang, Liang Duan, Bo Wang, Di Mu, Juan Chen, Ying Huang, Qin Hu, Weixian Chen

**Affiliations:** 1 Department of Laboratory Medicine, The Second Affiliated Hospital of Chongqing Medical University, Chongqing, China; 2 The Key Laboratory of Molecular Biology of Infectious Diseases Designated by the Chinese Ministry of Education, Chongqing Medical University, Chongqing, China; 3 Department of Infectious Diseases, The Second Affiliated Hospital, Chongqing Medical University, Chongqing, China; Central University of Tamil Nadu, INDIA

## Abstract

Killer cell lectin-like receptor G1 (KLRG1) has traditionally been regarded as an inhibitory receptor of T cell exhaustion in chronic infection and inflammation. However, its exact role in hepatitis B virus (HBV) infection remains elusive. CD8+ T cells from 190 patients with chronic hepatitis B were analyzed ex vivo for checkpoint and apoptosis markers, transcription factors, cytokines and subtypes in 190 patients with chronic hepatitis B. KLRG1+ and KLRG1− CD8+ T cells were sorted for transcriptome analysis. The impact of the KLRG1-E-cadherin pathway on the suppression of HBV replication mediated by virus-specific T cells was validated in vitro. As expected, HBV-specific CD8+ T cells expressed higher levels of KLRG1 and showed an exhausted molecular phenotype and function. However, despite being enriched for the inhibitory molecules, thymocyte selection-associated high mobility group box protein (TOX), eomesodermin (EOMES), and Helios, CD8+ T cells expressing KLRG1 produced significant levels of tumour necrosis factor (TNF)-α, interferon (IFN)-γ, perforin, and granzyme B, demonstrating not exhausted but active function. Consistent with the in vitro phenotypic assay results, RNA sequencing (RNA-seq) data showed that signature effector T cell and exhausted T cell genes were enriched in KLRG1+ CD8+ T cells. Furthermore, in vitro testing confirmed that KLRG1−E-cadherin binding inhibits the antiviral efficacy of HBV-specific CD8+ T cells. Based on these findings, we concluded that KLRG1+ CD8+ T cells are not only a terminally exhausted subgroup but also exhibit functional diversity, despite inhibitory signs in HBV infection.

## Introduction

Infection with the hepatitis B virus (HBV) is a significant global health issue that affects almost 300 million people worldwide. It can lead to severe liver diseases including acute and chronic hepatitis, cirrhosis, and hepatocellular carcinoma (HCC) [1, 2]. Current antiviral therapies are based on the use of reverse transcriptase inhibitors, including nucleoside and nucleotide analogs (NUCs) and interferon (IFN) [3], which can effectively suppress viral replication and significantly improve the disease course. Nevertheless, the incidence of functional cure remains low even after years of regular antiviral therapy [4].

Hepatitis B virus-specific CD8+ T lymphocytes are the main effector of viral clearance and liver inflammation in cases of infection with HBV [5, 6]. The principal function of these cells is to clear the virus by secreting proinflammatory cytokines as part of either cytolytic (mainly perforin and granzyme) or non-cytolytic(mainly IFN-γ and tumour necrosis factor [TNF]-α) processes [7]. In cases of antigen persistence, such as during viral infection or when a tumor is present, CD8+ T cells can become functionally inept, a condition known as T cell exhaustion [8]. Notably, one study found that although T cell exhaustion was common, most of the patients in the study were not significantly clinically immunocompromised, and that a proportion of the patients showed more effective HBV-specific or non-specific CD8+ T cell subsets [9].

T cell exhaustion was first identified in chronic lymphocytic choriomeningitis virus infection [10], and subsequent studies confirmed that it occurs in many other types of chronic viral infections, including infection with human immunodeficiency virus (HIV) [11, 12], hepatitis C virus (HCV) [13], and HBV [14], and in cases of autoimmune diseases [15]. An essential feature of exhausted CD8+ T cells is the continuous expression of inhibitory receptors, including the classical molecules of programmed cell death protein 1 (PD-1), T cell immunoglobulin and mucin domain 3 (TIM-3), and lymphocyte-activation gene 3 (Lag-3) [16]. In addition, the killer cell lectin-like receptor G1 (KLRG1) is a transmembrane glycoprotein with an extracellular C-type lectin-like domain and a cytoplasmic immunoreceptor tyrosine-based inhibitory motif (ITIM) [17], plays a critical role in immune cell exhaustion: interaction between KLRG1 and E-cadherin has been shown to induce functional inhibition in T lymphocytes and natural killer (NK) cells [18]. Furthermore, there are high levels of co-expression between KLRG1 and other inhibitory receptors, including PD-1, in CD8+ T cells in individuals infected with Epstein-Barr virus (EBV) or HBV [19, 20].

Data from several clinical trials have shown that KLRG1 expression is elevated in lymphocytes—particularly in CD8+ T lymphocytes—during chronic human viral infections, including infection with cytomegalovirus (CMV), HIV, and EBV, but reduced in cases of acute self-limited infection (e.g., in infection with influenza virus) [21–23]. In a recent study, CD8+ T cells from long-term antiviral-treated chronic hepatitis B (CHB) patients demonstrated higher KLRG1 expression than CD8+ T cells from healthy controls (HCs) [24]. In addition, another study showed that KLRG1 expression was significantly downregulated in HBV-specific CD8+ T cells in the intrahepatic compartment compared to that in peripheral blood mononuclear cells (PBMCs) [25]. Yet, to date, the role that KLRG1 plays in HBV-specific or non-specific CD8+ T cells in naive CHB patients remains unknown.

To further understand the role that KLRG1+ cells play in CHB, in this study, we determined the presence and characteristics of KLRG1+ HBV-specific and non-specific CD8+ T cells in the peripheral blood of CHB patients. We also analyzed the expression profiles of these cells and their associations with virological indicators or the degree of liver inflammation. The findings highlight the implications that KLRG1 expression has for CHB patients who have not received treatment and offer new insights into potential immunotherapeutic approaches for treating HBV infection.

## Materials and methods

### Study cohort

Between September 1, 2021, and March 20, 2023, blood samples were obtained from 190 CHB patients recruited by the Institute of Viral Hepatitis of the Second Affiliated Hospital of Chongqing Medical University and 58 age-matched HCs enrolled in the physical examination center. All the recruited CHB patients were outpatients who were not on antiviral therapy. They were classified into the different clinical phases of chronic HBV (cHBV) infection according to the 2017 European Association for the Study of the Liver guideline [26], which considers the presence of hepatitis B e antigen (HBeAg), HBV DNA levels, transaminase levels (alanine aminotransferase [ALT] and aspartate aminotransferase [AST]), and the presence or absence of liver inflammation.

For this study, 46 patients with HBeAg-positive cHBV infection (HBeAg+ cHBV), 34 patients with HBeAg-positive CHB (HBeAg+ CHB), 89 patients with HBeAg-negative cHBV infection (HBeAg− cHBV), and 21 patients with HBeAg-negative CHB (HBeAg− CHB) were enrolled. CHB and cHBV are distinguished by the presence or absence of liver inflammation; in CHB, liver inflammation is present, while is it absent in patients infected with cHBV. The characteristics of these patients enrolled in this study are shown in Table 1.

**Table 1:** Patient characteristics.

Clinical data	Healthy controls	HBeAg-positive cHBV infection	HBeAg-positive CHB	HBeAg-negative cHBV infection	HBeAg-negative CHB
	(HCs)	(HBeAg+ cHBV)	(HBeAg+ CHB)	(HBeAg− cHBV)	(HBeAg− CHB)
N	58	46	34	89	21
Gender (male/female)	45/13	18/28	21/13	42/47	14/7
Age (years)	25 (20−45)	27 (18−42)	30 (19−45)	34 (18−45)	34 (21−45)
ALT (U/L)	18.5 (15−38)	22.5 (10−38)	83.5 (40−393)	22 (7−39)	48 (40−513)
AST (U/L)	20 (10−40)	21 (11−66)	47.5 (17−148)	22 (11−41)	35.5 (21−212)
HBsAg (IU/mL)	negative	52273.8 (3634.2−115548.4)	38171.3 (4.1−124252.1)	656.4 (0.1−41013.7)	2078.6 (46.9−20339.8)
HBeAg (PEIU/mL)	negative	1551.3 (2−2565)	1172.7 (3−1776)	negative	negative
HBV DNA (Log_10_ IU/mL)	n.a.	8.3 (7.1−8.9)	7.8 (4.4−9)	2.3 (1−3.2)	5 (3.5−8.4)

All numerical data are expressed as medians (range).

N, number of cases; ALT, alanine aminotransaminase; AST, aspartate aminotransferase;HBV, hepatitis B virus; CHB, chronic hepatitis B; HBeAg, hepatitis B e antigen.

### Flow cytometry

Single-cell suspensions were stained with monoclonal antibodies (mAbs) specific for human CD3, CD8, CD4, CCR7, KLRG1, CD45RA, CD127, PD-1, CD69, TIM-3, and Lag-3. For intranuclear staining, cells were fixed/permeabilized using theTranscription Factor Buffer Set from BD Bioscience (USA), following the manufacturer’s instructions. Intranuclear staining of fixed human cells was performed using antibodies specific for human T cell factor 1 (TCF-1), Helios, eomesodermin (EOMES), thymocyte selection-associated high mobility group box protein (TOX), and T-box expressed in T cells (T-bet). For the intracellular cytokine assay, cells were stimulated with 1 μL of phorbol myristate acetate/ionomycin mixture (250 X) (Multi Sciences, China) at 37°C for 1 h. Then, 1 μL of brefeldin A/monensin mixture (250 X) (Multi Sciences, China) was added to continuously block the transport of cytokines for 4 h. Intracellular staining was performed using the Cytofix/Cytoperm™Fixation/Permeabilization Kit from BD Bioscience (USA), and cells were stained with labeled anti-IL-2, anti-TNF-α, anti-granzyme B, and anti-perforin antibodies. All flow cytometry/cell analysis panels and reagent lists are available in the supplementary materials.

### Expansion of virus-specific CD8+ T cells and assessment of CD8+ T-cell function

Virus-specific CD8+ T cells populations were expanded as previously described (20). In brief, PBMCs (2×10^6^ cells) were cultured in RPMI 1640 (Thermo Scientific, USA) supplemented with 10% human serum (GeminiBio, USA) after thawing. Cells were stimulated with HBc18−27 (10μg/mL, FLPSDFFPSV, Sangon Biotech, China) and an anti-CD28 mAb (0.5 μg/ml, BD Bioscience, Germany) on day 1, supplemented with IL-2 (20U/mL, Peprotech, USA) every three days, and harvested on day 14. Then, dextramer staining and cytokine production assays were performed as previously described [27].

### Statistics

For analyses that included more than two groups, the Kruskal−Wallis test was used. Post hoc analyses were performed using Dunnett’s multiple comparison test. The paired Student’s t-test was used to compare matched samples, while the unpaired Student’s t-test or Wilcoxon test was used for unmatched samples. Data are shown as the mean ± standard deviation (SD) or median value with the interquartile range (IQR). P-values below 0.05 were considered significant. The statistical analysis was performed using SPSS 22.0 (IBM).

### Ethics statement

The study was conducted according to the guidelines of the Declaration of Helsinki, and approved by the Ethics Committee of The Second Affiliated Hospital of Chongqing Medical University (2016–12).

## Results

### KLRG1 expression strongly correlates with the dysfunction of HBV-specific CD8+ T cells in chronic HBV infection

**Fig 1:**
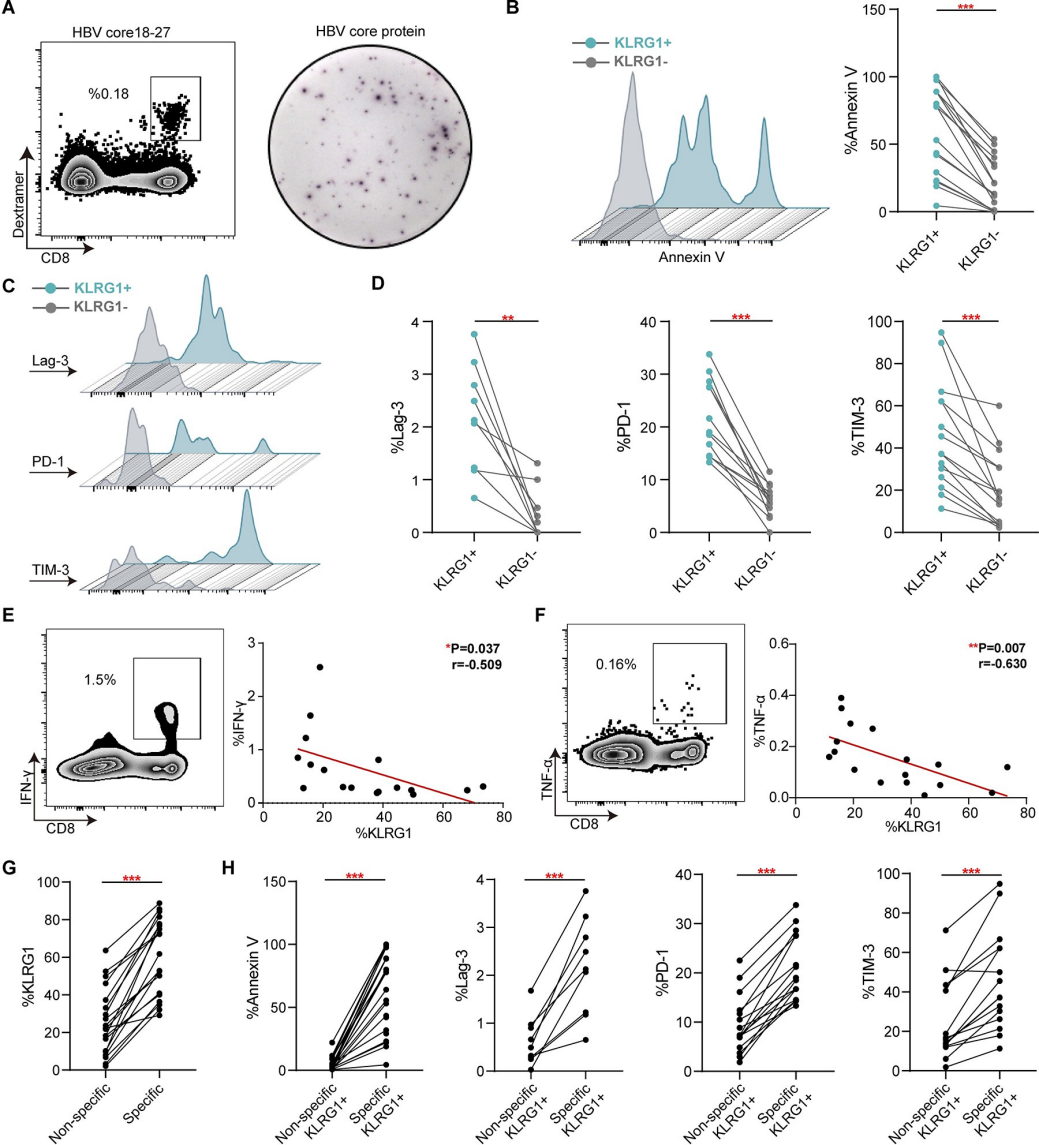
KLRG1 expression was linked to HBV-specific CD8+ T cell exhaustion in chronic HBV infection. **(A)** Representative plots of HBc18−27-specific CD8+ T cells and HBcAg-specific CD8+ T cells. **(B**−**D)** Representative flow cytometric histograms and expression of annexin V and inhibitory molecules (Lag-3, PD-1, and TIM-3) in KLRG1+ versus KLRG1− HBc18−27-specific CD8+ T cell populations. ***p < 0.001, **p < 0.01, paired Student’s t-test. **(E**−**F)** Representative dot plots of cytokines (TNF-α and IFN-γ) production and their correlation with KLRG1 expression. **p < 0.01, *p < 0.05, Pearson R correlation. **(G)** Expression of KLRG1 in HBV-specific CD8+ T cells versus non-specific CD8+ T cells. ***p < 0.001, paired Student’s t-test. **(H)** Expression of Annexin V and inhibitory molecules (Lag-3, PD-1, and TIM-3) in KLRG1+ HBV-specific CD8+ T cells versus KLRG1+ non-specific CD8+ T cells. ***p < 0.001, paired Student’s t-test.

After successfully expanding populations of HBV-specific CD8+ T cells (Fig 1A), we examined the occurrence of apoptosis and inhibitory molecules in populations from 15 CHB patients. Typically, the histograms and pairwise analysis results indicated that there was more apoptosis in HBV-specific KLRG1+ CD8+ T cells than in KLRG1− CD8+ T cells (Fig 1B). Next, we found that all three inhibitory markers (Lag-3, PD-1 and TIM-3) were more highly expressed in KLRG1+ HBV-specific CD8+ T cells versus KLRG1− HBV-specific CD8+ T cells (Fig 1C and 1D). Distinctively, we also observed an inverse correlation between KLRG1 expression and HBV-specific cytokine production (IFN-γ and TNF-α) (Fig 1E and 1F). Furthermore, we found considerably lower levels of KLRG1 expression in non-specific CD8+ T cells compared to HBV-specific CD8+ T cells, as well as lower amounts of annexin V and inhibitory molecules (Fig 1G and 1H). These results imply that KLRG1 is not only a marker for HBV-specific CD8+ T cells with the exhaustion phenotype but also strongly linked to functional impairment.

### Differential KLRG1 expression in CD8+ T cells from CHB patients is linked to the clinical phases of infection

**Fig 2:**
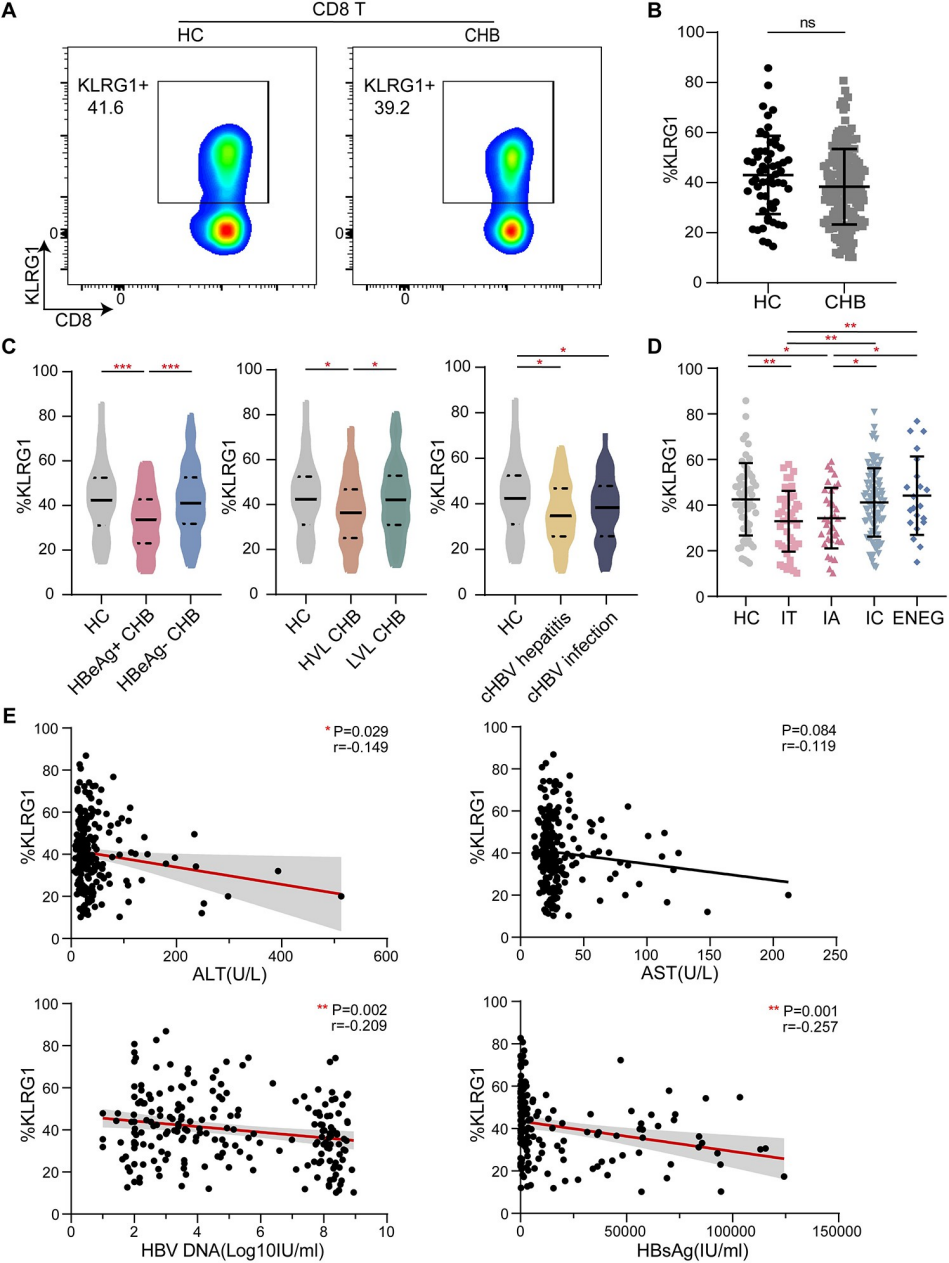
KLRG1 expression in CD8+ T cells was associated with different phases of chronic HBV infection. **(A)** Representative flow cytometric plots showing the data from one healthy control (HC) and one chronic hepatitis B (CHB) patient. The plots show the frequency of peripheral blood CD8+ T cells based on the surface expression of KLRG-1. **(B)** KLRG1 expression in CD8+ T cells from HCs and CHB patients. Data are shown as median values with IQRs. ns, not significant (*p* > 0.05), Wilcoxon test. **(C)** KLRG1 expression in CD8+ T cells according to a single indicator: HBeAg, HBV DNA, or ALT. Data are shown as median values with IQRs. ***p < 0.001, *p < 0.05, Kruskal−Wallis test with Dunnett’s multiple comparison test. **(D)** Analysis of KLRG1 expression in CD8+ T cells obtained from HCs and patients with different phases of CHB. Data are shown as median values with IQRs. **p < 0.01, *p < 0.05, Kruskal−Wallis test with Dunnett’s multiple comparison test **(E)** Correlation analysis of serum ALT, AST, HBV DNA, and HBsAg and the frequency of KLRG1-expressing CD8+ T cells from the CHB patients are depicted, respectively. **p < 0.01, *p < 0.05, Pearson R correlation.

In the second set of experiments, we analyzed the expression of KLRG1 in CD8+ T cells from 190 patients chronically infected with HBV and 58 age-matched HCs. The representative pseudocolor images of the two populations are shown in Fig 2A. The total T cell and CD8+ T cell frequencies were significantly lower in the samples from the CHB patients (S2 Fig); however, the KLRG1 expression in the CD8+ T cells from the CHB patients was not significantly different from that in the cells from the HCs (Fig 2B). The expression of KLRG1 was significantly lower in the HBeAg-positive patients than in the HCs; however, there was no significant difference in the KLRG1 expression between the HBeAg-negative patients and the HCs (Fig 2C).

When we examined KLRG1 expression in terms of low-level viremia (LLV) [28], we found that KLRG1 expression was significantly lower in patients with HBV DNA levels ≥2000 IU/mL compared to that in both the HCs and patients with HBV DNA levels <2000IU/mL (Fig 2C). However, only the chronic infection (ALT < 40 U/L) and chronic hepatitis (ALT > 40 U/L) groups were found to exhibit this reduction in KLRG1 expression compared to the HCs (Fig 2C). Interestingly, KLRG1 was more highly expressed in patients classified as HBeAg− cHBV and HBeAg− CHB than in patients classified as HBeAg+ cHBV and HBeAg+ CHB, respectively (Fig 2D). Correlation analysis showed that high expression of KLRG1 was accompanied by low levels of HBV DNA and HBsAg. Meanwhile, KLRG1 expression was negatively correlated with ALT and AST levels (Fig 2E). Thus, our results showed that KLRG1 was differentially expressed in peripheral blood CD8+ T cells from untreated CHB patients and that the cells did not exhibit any overall differences when compared to those from HCs.

### KLRG1 expression is associated with phenotypic characteristics of non-specific CD8+ T cell exhaustion

**Fig 3:**
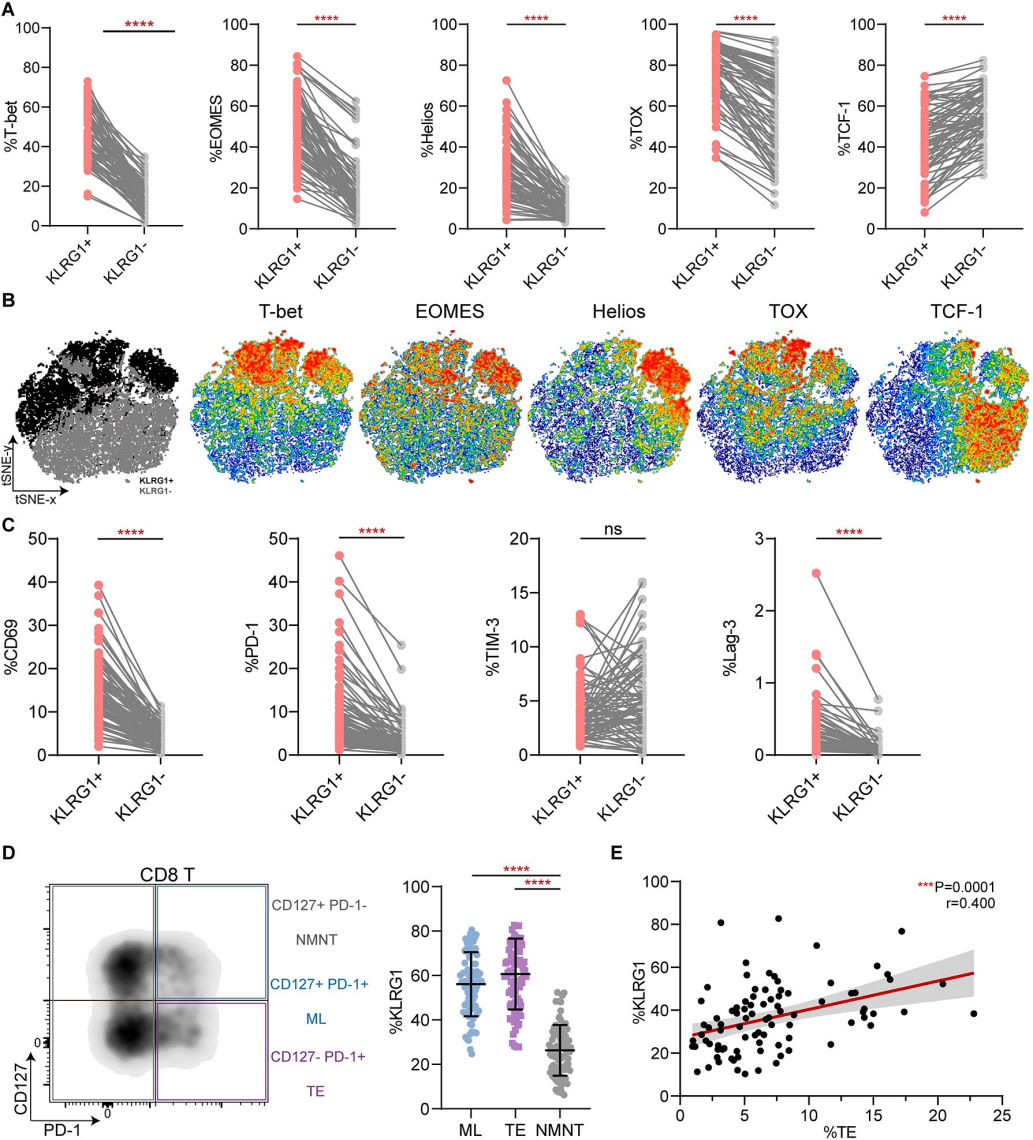
KLRG1-expressing CD8+ T cells exhibited a phenotypic profile of T cell exhaustion. **(A)** Differential expression of the transcription factors T-bet, EOMES, Helios, TOX, and TCF-1 in KLRG1+ and KLRG1− CD8+ T cells. ****p < 0.0001, paired Student’s t-test. **(B)** t-SNE representation analysis of concatenated flow cytometry data obtained from KLRG1+ and KLRG1− CD8+ T cells. Expression levels of T-bet, EOMES, Helios, TOX, and TCF-1 are plotted on the t-SNE plot. **(C)** The expression of membrane surface molecules (CD69, PD-1, TIM-3, and Lag-3) in KLRG1+ and KLRG1− CD8+ T cell was assessed. Representative flow cytometric histograms are shown that include the gating of the individual markers. ns, not significance (*p* > 0.05), ****p < 0.0001, paired Student’s t-test. **(D)** KLRG1 expression was determined in subsets of CD8+ T cells based on CD127 and PD-1 expression using flow cytometry. Representative flow density plots are shown. NMNE, CD127+ PD-1− non-memory non-exhausted T cells; ML, CD127+ PD-1+ memory like T cells; TE, CD127− PD-1+ terminally exhausted T cells. Data are shown as median values with IQRs. ****p < 0.0001, Kruskal−Wallis test with Dunnett’s multiple comparison test. **(E)** Correlation analysis of the frequency of the CD127/PD-1 subsets and the frequency of KLRG1-expressing CD8+ T cells from the CHB patients. Each dot represents one CD8+ T cell population. ***p < 0.001, Pearson R correlation. t-SNE, t-distributed stochastic neighbor embedding.

Compared with KLRG1− CD8+ T cells, KLRG1+ CD8+ T cells demonstrated higher expression of EOMES, Helios, and TOX (Fig 3A), which are reportedly classical transcription factors involved in T cell exhaustion [29–31]. T-bet, which has been shown to sustain exhausted CD8+ T cells during chronic viral infection [31], was also highly expressed in the KLRG1+ CD8+ T cells (Fig 3A). Conversely, low expression of TCF-1 was observed in the target cell population (Fig 3A). As previously reported, TCF-1 is vital to maintaining the virus-specific CD8+ T cell response [32, 33]. We concatenated all the files from the 89 patients with CHB into a single file and performed a t-distributed stochastic neighbor embedding (t-SNE) analysis. The results are shown in Fig 3B, and the gray and black dots represent KLRG1− and KLRG1+ CD8+ T cells, respectively. We then generated scatter plots to examine the expression in representative individuals (Fig 3C). The general activating molecule CD69 was strongly expressed in KLRG1+ CD8+ T cells. We also observed PD-1 and Lag-3,which are markers of T-cell exhaustion, were highly expressed by KLRG1+ subsets. Then, based on their differential expression of PD-1 and CD127, the CD8+ T cells were divided into three subsets: CD127+ PD-1+ memory like (ML) T cells, CD127− PD-1+ terminally exhausted (TE) T cells, and CD127+ PD-1− non-memory non-exhausted (NMNE) T cells (Fig 3D). We noticed that both ML and TE T cells had significantly higher levels of KLRG1 than NMNE T cells (Fig 3D). Indeed, we identified a clear correlation between the frequency of TE T cells and KLRG1 expression in the CHB patients (Fig 3E). Together, these results indicate that KLRG1 is co-expressed with other inhibitory checkpoint molecules in cHBV infection.

### KLRG1+ CD8+ T cells exhibit greater cytotoxicity than KLRG1− CD8+ T cells

**Fig 4:**
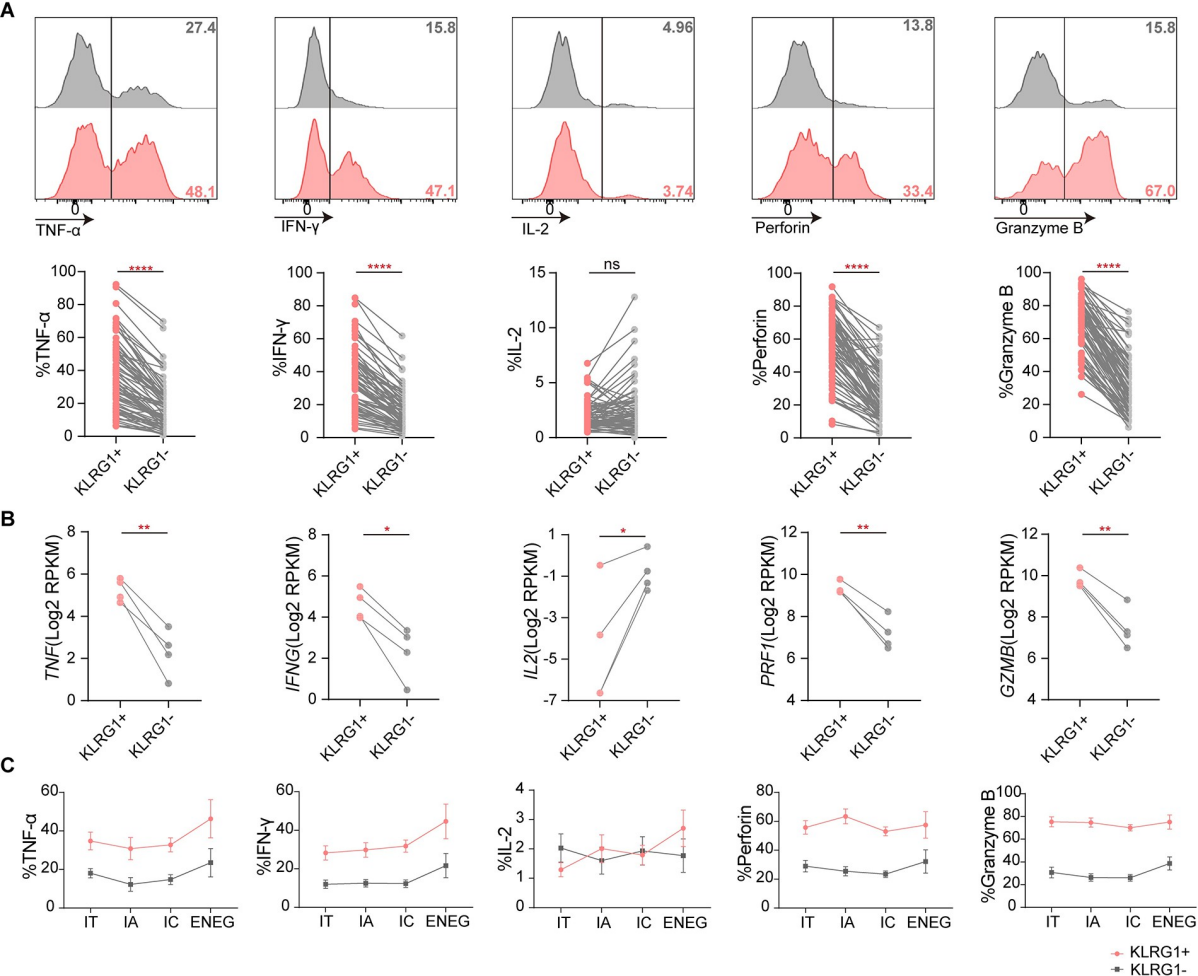
KLRG1 expression induced strong cytotoxic activity via increased release of proinflammatory cytokines in chronic HBV infection. **(A)** Expression of TNF-α, IFN-γ, IL-2, perforin, and granzyme B in KLRG1+ and KLRG1− CD8+ T cell populations from all the CHB patients. Representative flow cytometric histograms, with gating of the individual markers, are displayed. **(B)** Expression of *TNF* (i.e., TNF-α), *IFNG* (i.e., IFN-γ), *IL2* (i.e., IL-2), *PRF1* (i.e., perforin), *GZMB* (i.e., granzyme B) was assessed in KLRG1+ and KLRG1− CD8+ T cells at the mRNA level. **(C)** Expression of TNF-α, IFN-γ, IL-2, perforin, and granzyme B in KLRG1+ and KLRG1− CD8+ T cells from patients classified with HBeAg+ CHB, HBeAg+ cHBV, HBeAg− cHBV, and HBeAg− CHB. ns (*p* > 0.05), not significance, **p < 0.01, *p <0.05, ****p <0.0001, paired Student’s t-test.

In Fig 4, a representative histogram based on the data from one patient is shown, as well as an analysis of data from 76 CHB patients. Almost all the detected cytokines, including TNF-α, IFN-γ, perforin, and granzyme B, were highly expressed in KLRG1+ T cells, although we noted that there was somewhat lower expression of IL-2 in KLRG1+ CD8+ T cells compared to KLRG1− CD8+ T cells (Fig 4A). In addition, the transcriptomic data suggested that at the mRNA level, KLRG1+ CD8+ T cells had higher expression of *TNF* (i.e., TNF-α), *IFNG* (i.e., IFN-γ), *PRF1* (i.e., perforin), and *GZMB* (i.e., granzyme B), despite low levels of *IL2* (i.e., IL-2) expression (Fig 4B). These consistent results implied that the antiviral function of KLRG1+ CD8+ T cells was barely suppressed during HBV infection. As shown in the broken-line graph in Fig 4C, the magnitude of transformation was almost the same in the two opposing cell populations; the exception was that IL-2 exhibited the reverse tendency. Overall, these results indicate that KLRG1+ non-specific CD8+ T cells exhibit sustained cytotoxic activity in infected patients.

### Elevated expression of KLRG1 was associated with both short-term effector and memory T cells in CHB

**Fig 5:**
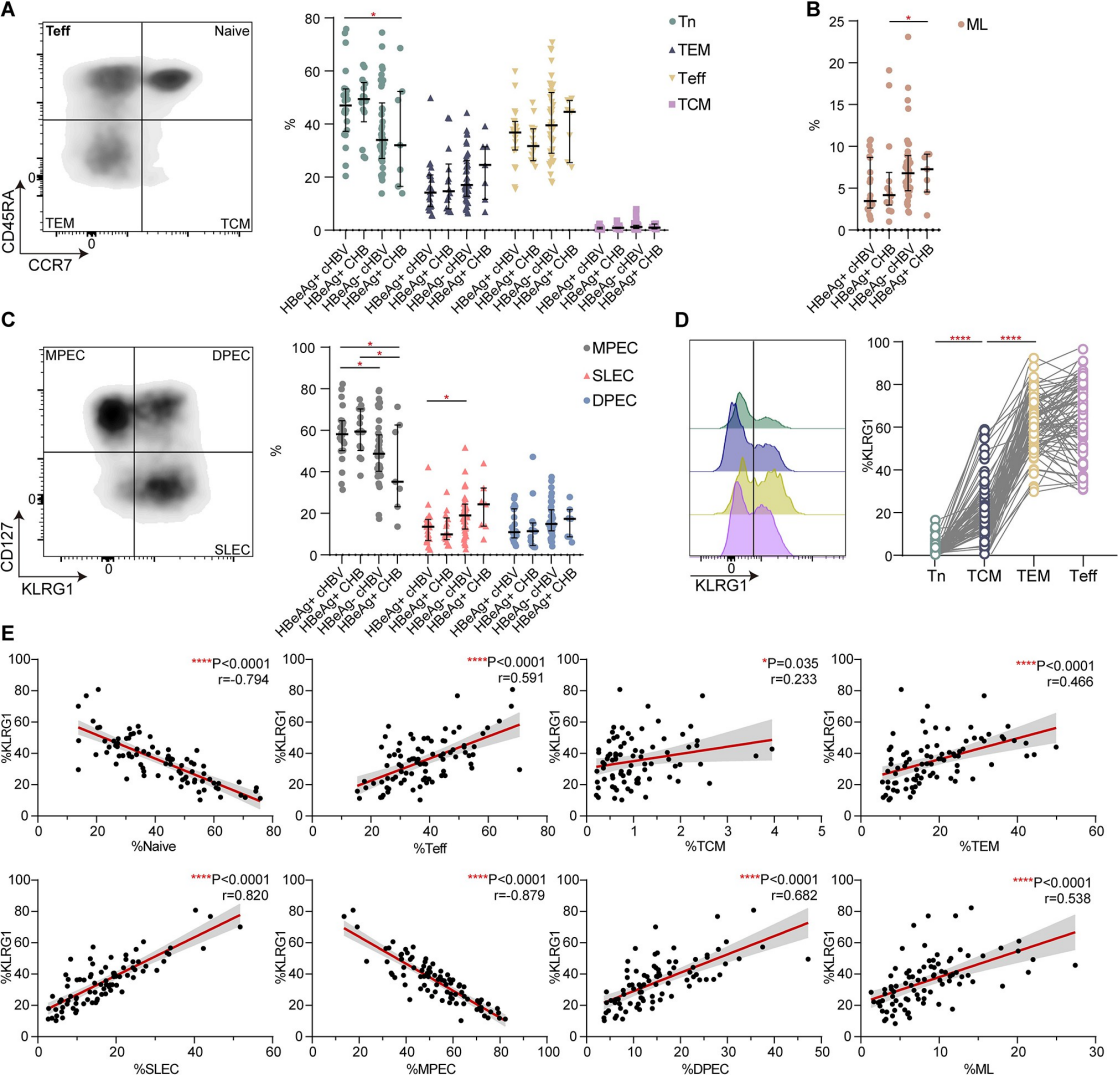
Elevated expression of KLRG1 prompted the acquisition of effector and memory functions in CD8+ T cells. **(A)** CD45RA/CCR7 expression was determined during four phases of CHB. A representative flow cytometry contour plot is shown. Teff, CD45RA+ CCR7− cells; Tn, CD45RA+ CCR7+ Tn cells; TEM, CD45RA− CCR7− cells; and TCM, CD45RA− CCR7+ cells. Data are shown as median values with IQRs. *p < 0.05, Kruskal−Wallis test with Dunnett’s multiple comparison test. **(B)** Alteration of ML cells in different stages of hepatitis B infection. Data are shown as median values with IQRs. *p < 0.05, Kruskal−Wallis test with Dunnett’s multiple comparison test. **(C)** CD127/KLRG1 expression was detected during four phases of CHB. A representative flow cytometry contour plot is shown. MPEC, CD127+ KLRG1− cells; DPEC, CD127+ KLRG1+ cells; and SLEC, CD127− KLRG1+ cells. Data are shown as median values with IQRs. *p < 0.05, Kruskal−Wallis test with Dunnett’s multiple comparison test. **(D)** Data from one patient was used to produce the representative histogram, and data from 78 CHB patients was used to produce the second plot. The results showed elevated expression of KLRG1 in the TCM, TEM, and Teff subsets compared to the Tn subset. ****p < 0.0001, paired Student’s t-test. **(E)** Correlation analysis of the frequency of CD127/PD-1 or CD45RA/CCR7 CD8+ T cell subsets and the frequency of KLRG1-expressing CD8+ T cells obtained from all the CHB patients. Each dot represents one CD8+ T cell population. ****p < 0.0001, Pearson R correlation.

Based on the differential expression of CD45RA and CCR7, CD8+ T cells from the 89 CHB patients were divided into four subsets: CD45RA+ CCR7− effector T (Teff) cells, CD45RA− CCR7− effector-memory (TEM) cells, CD45RA+ CCR7+ naive T (Tn) cells, and CD45RA− CCR7+ central-memory T (TCM) cells [34]. We found that the percentage of Tn cells was greater in the HBeAg+ cHBV and HBeAg+ CHB patients than in the HBeAg− cHBV and HBeAg+ CHB patients. The trends for the Teff, TCM, and TEM cells were less pronounced (Fig 5A). There was also a slight increase in the ML T cells in the HBeAg− cHBV and HBeAg+ CHB patients (Fig 5B). The surface molecules CD127 and KLRG1 are often used as markers to predict the memory or effector potential of activated CD8+ T cells, with short-lived effector cells (SLEC) identified as CD127− KLRG1+, memory precursor effector cells (MPEC) identified as CD127+ KLRG1− and double-positive effector cells (DPEC) identified as CD127+ KLRG1+ [35]. Notably, the, SLEC% and DPEC% values were higher in the former two phases compared to the latter two phases, while the MPEC% values showed the opposite trend (Fig 5C). The results presented in the representative histograms and dot plots shown in Fig 5D indicated that memory and effector subpopulations unsurprisingly expressed higher levels of KLRG1. As expected, we observed that as the KLRG1+% value increased, the proportions of the naive CD8+ T cells and MPEC tended to decrease. Indeed, the proportions of all the memory and SLEC, and even the precursor cells with the ability to develop into protective immune cells, improved as the KLRG1+% value increased (Fig 5E). Collectively, KLRG1 is detected in a considerable number of memory and effector T cell subsets, even during infection, and it is more prevalent in subsets from CHB patients with a greater capacity to suppress the virus.

### Transcriptional profiling identifies KLRG1+ CD8+ T cells as concurrently enriched in “effector” and “exhaustion” molecules

**Fig 6:**
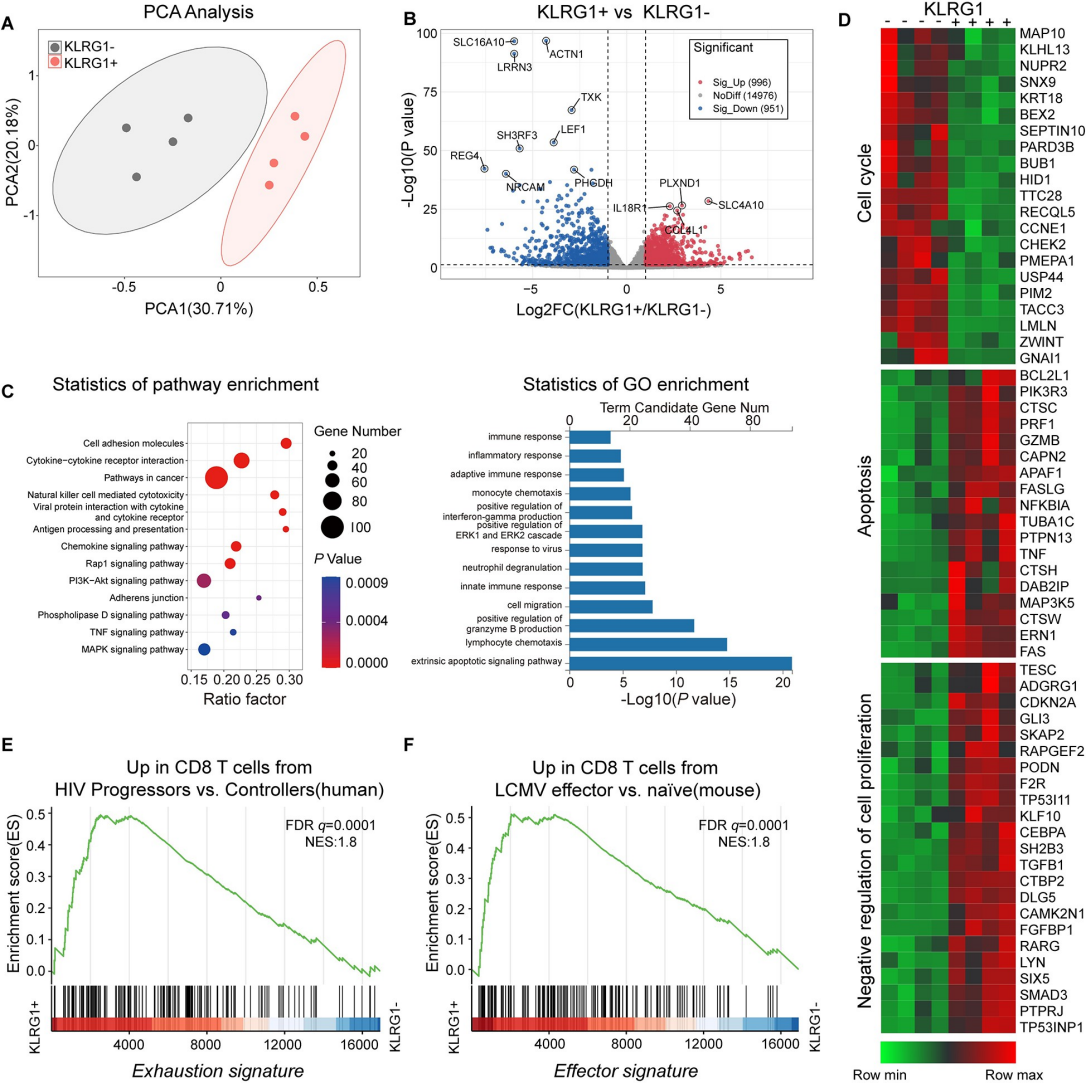
KLRG1+ CD8+ T cells from CHB patients exhibited a distinctive transcriptional profile. Whole-transcriptome sequencing and analysis were performed using sorted KLRG1+ and KLRG1− CD8+ T cells from four strictly paired CHB patients. **(A)** Principal component analysis (PCA) of transcriptomic data (KLRG1 + = 4, KLRG1 − = 4). **(B)** Volcano plot (log10 P-values vs. log2 fold changes in expression) of transcripts differentially expressed in KLRG1+ CD8+ T cells and KLRG1− CD8+ T cells. sig, significant. **(C)** Gene Ontology (GO) and Kyoto Encyclopedia of Genes and Genomes (KEGG) analyses of the 1,947 differentially expressed genes (DEGs). **(D)** Heat map of genes representing apoptosis, cell cycle processes, and negative regulation of proliferation. **(E)** Enrichment of a previously published gene signature of CD8+ T cell exhaustion (described in ref. (36)) was assessed in KLRG1+ and KLRG1− CD8+ T cells from CHB patients by gene set enrichment analysis (GSEA). **(F)** Enrichment of a previously published gene signature of effector CD8+ T cells (described in ref. (37)) was assessed in KLRG1+ and KLRG1− CD8+ T cells from CHB patients by GSEA. NES, normalized enrichment score.

Next, we performed an unbiased microarray analysis of freshly sorted KLRG1+/− CD8+ T cells from four CHB patients. An unsupervised principal component analysis (PCA) of the data showed that both KLRG1+ and KLRG1− CD8+ T cells clustered into two distinct groups (Fig 6A). The volcano plot displayed 1947 differentially expressed genes (DEGs), of which 996 were upregulated and 951 were downregulated (log2[fold change]>1, Padj<0.05) (Fig 6B). We selected representative genes and conducted a detailed enrichment pathway analysis using Gene Ontology (GO) terms and Kyoto Encyclopedia of Genes and Genomes (KEGG) (https://www.genome.jp/kegg/) pathways. The results showed that KLRG1+ T cells were enriched in molecules involved in responses to viruses, and antigen presentation processes, and subsequent immune and inflammatory responses (Fig 6C). The resultant representative heat maps showed that cell cycle-related genes were significantly under-expressed in KLRG1+ T cells and that changes in the apoptotic process coincided with the opposite. Moreover, genes that negatively regulate cell proliferation were also significantly enriched in KLRG1+ T cells (Fig 6D).

Next, we compared the gene expression profile with the known expression signature of exhausted cells via a gene set enrichment analysis (GSEA). The gene signature of previously described exhausted CD8+ T cells from HIV patients with progressive disease [36] was found to be enriched in the KLRG1+ CD8+ T cells from CHB patients (Fig 6E). Interestingly, compared with the KLRG1− CD8+ T cells, the KLRG1+ CD8+ T cells also had an expression profile that was enriched in signature effector CD8+ T cell genes [37] (Fig 6F). Taken together, these findings indicate that the KLRG1-expressing CD8+ T cells have an exhausted molecular signature, despite the transcriptional analysis showing that they are functionally effective.

### Addition of sE-cadherin promotes dysfunction of antiviral activity in HBV-specific CD8+ T cells

**Fig 7:**
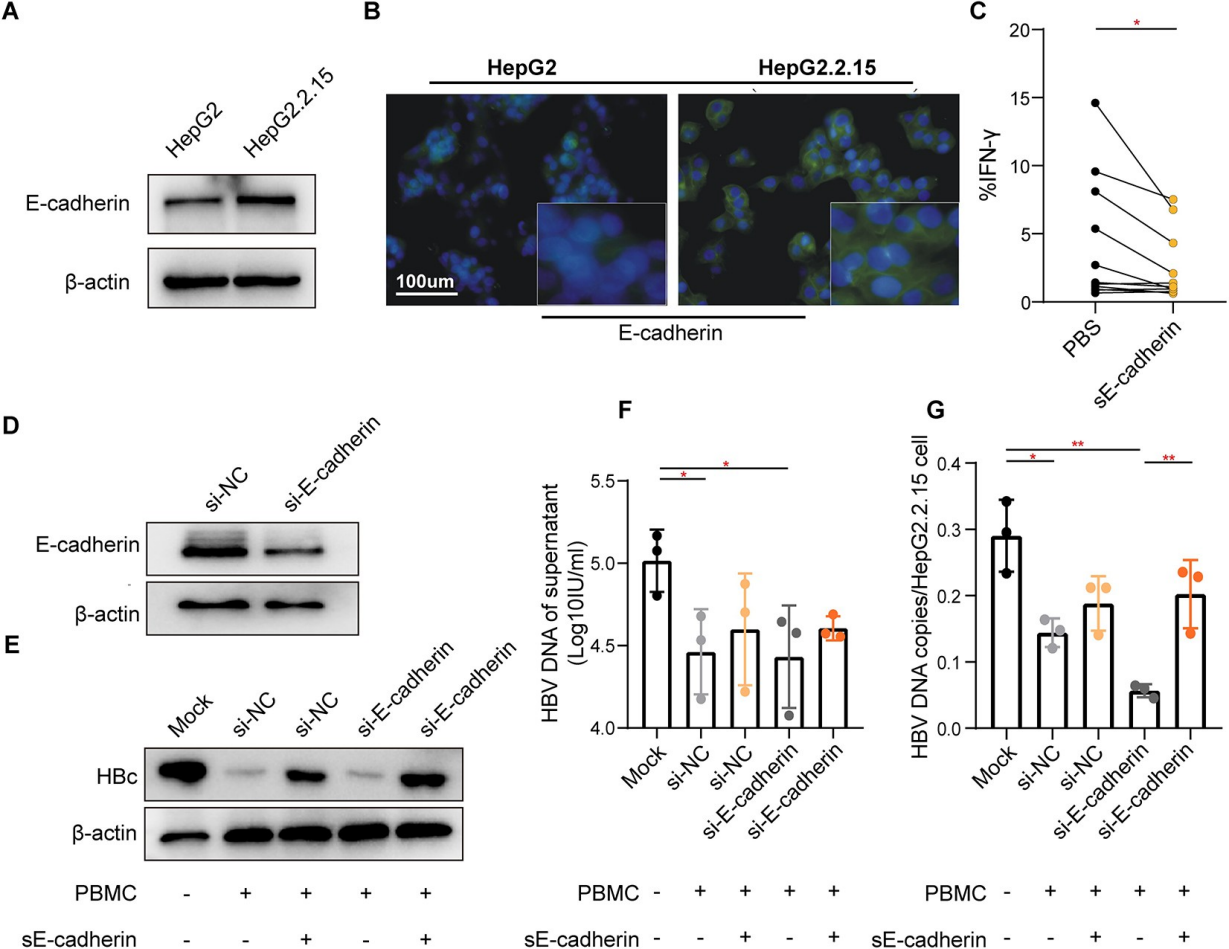
The combination of sE-cadherin and KLRG1 antagonized the inhibitory effect of KLRG1+ CD8+ T cells on HBV replication. E-cadherin expression was higher in HepG2.2.15 cells than in HepG2 cells, as shown by the Western blotting (A) and immunofluorescence (B) results. (C) The representative histogram was produced using data from eight CHB patients. The results showed reduced IFN-γ secretion by global CD8+ T cells in the presence of sE-cadherin. *p < 0.05, paired Student’s t-test. (D) Effect of using siRNA to knockdown E-cadherin in HepG2.2.15 cells. Reduction in level of HBV core protein (E), HBV DNA in supernatant (F), and HBV DNA copies/cells (G) in HepG2.2.15 cells after co-culture with HBc18−27-stimulated PBMCs. Data are shown as mean ± SD, **p < 0.01, *p < 0.05, unpaired Student’s t-test.

Considering E-cadherin is a unique and inhibitory ligand for KLRG1 [38], we investigated the level of soluble E-cadherin (sE-cadherin) present in patient serum samples and a cell line (HepG2.2.15) to confirm the functional connection between the E-cadherin−KLRG1 signaling pathway and virus-specific T lymphocytes. As expected, the concentration of sE-cadherin was significantly higher in the patient samples, particularly in those from patients with a higher degree of liver inflammation (S5 Fig). Significantly higher E-cadherin expression was also observed in HepG2.2.15 cells, which sustained continuous HBV replication compared to HepG2 cells (Fig 7A and 7B). sE-cadherin reduced IFN-γ secretion in HBV-specific KLRG1+ CD8+ T cells (Fig 7C). To further elucidate the observed sE−cadherin-KLRG1 signaling-induced HBV-specific T cell dysfunction, we knocked down E-cadherin in HepG2.2.15 cells using small interfering RNA. (Fig 7D). Interestingly, HBV-specific CD8+ T cells did inhibit HBV replication in HepG2.2.15 cells, and the suppressed viral replication could be restored to some extent by the addition of sE-cadherin (Fig 7E–7G). Moreover, the number of HBV DNA copies/cell was more significantly decreased in the silenced E-cadherin cells (Fig 7G). These findings indicate that KLRG1 functions as a negative immune regulator when combined with its ligand, E-cadherin.

## Discussion

Under sustained stimulation by tumor and pathogen antigens, the immune system’s defenses become ineffective [39], and immune cells eventually lose their effector function, a process known as immune cell exhaustion [40]. This process involves a variety of cells, including T cells, B cells and NK cells [41, 42]. During chronic hepatitis B infection, HBV-specific B cells differentiate into an atypical phenotype that has difficulty producing anti-hepatitis B surface antigen (HBsAg) antibodies [43], whereas NK cells upregulate TIM-3 and T-cell immunoreceptor with Ig and ITIM domains (TIGHT) and exhibit reduced killing and cytokine production [44]. Atypical T cells, such as invariant natural killer T (iNKT) cells and mucosal associated invariant T (MAIT) cells, should not be disregarded. Peripheral MAIT cells in chronic hepatitis B patients have been shown to exhibit significant levels of T-cell exhaustion genes and to be functionally impaired [45, 46]; iNKT cells, on the other hand, have been shown to have lower growth rates and impaired IFN-γ production [47]. Currently, the most extensive and important research is focusing on CD8+ T cells, as they are the most abundant and vital component of adaptive immunity. In this study, we examined the role that KLRG1-expressing CD8+ T cells play during the natural course of cHBV infection to determine whether the phenotype and function of these cells differ.

Initially, in line with other studies [20, 48], we discovered that KLRG1 was produced at higher levels by HBV-specific CD8+ T cells and that these cells were unquestionably phenotypically deficient and functionally compromised. Interestingly, compared to the HBV-specific T cell fraction, the examined CD8+ T cells produced considerably fewer suppressor molecules and were less likely to undergo apoptosis. Therefore, based on this observed differentiation, we conducted an extensive analysis of CD8+ T cells in CHB patients. The most striking finding was that, in contrast to Rinker et al.’s findings [24], KLRG1 expression in HBV non-specific CD8+ T cells did not significantly differ between HCs and CHB patients. To confirm this finding, we examined a larger group. We then concluded that the observed findings may have been due to the fact that we included naive CHB patients rather than treated patients. Differences in patient characteristics can lead to distinct outcomes. In addition, our research and that of others [5] has indicated that HBV-specific CD8+ T cells account for less than 1% of peripheral blood cells, and this factor may have disguised the considerable overexpression of KLRG1 in HBV-specific CD8+ T cells.

Another important discovery was that the studied KLRG1+ CD8+ T cells exhibited an inconsistency in terms of their phenotype and function; notably, they exhibited phenotypic exhaustion but retained their function. First and foremost, exhaustion signals were found to coexist with effector signals, as the transcriptome data clearly demonstrated. A previous study indicated that the T-bet−EOMES transcriptional regulatory axis is involved in T cell differentiation or dysfunction and that a massive enrichment of EOMES is strictly associated with the exhausted phenotype [30]. Subsequent studies have shown that in addition to EOMES, other transcription factors involved in T cell exhaustion [49], such as Helios and TOX, are highly expressed in non-specific KLRG1+ CD8+ T cells. Regarding the classical cell-surface inhibitory receptors PD-1 and Lag-3, we certainly observed parallel expression with KLRG1, as has been reported in some infections and cancers [50–52]. The overwhelmingly high expression of CD69 we observed in the presence of KLRG1 was also fully consistent with the activation of dysfunctional T cells. In addition to the clear positive relationship that was evident between KLRG1 and terminally exhausted cells, the transcriptome and cell typing results also suggested that the studied KLRG1+ CD8+ T cells exhibited exhaustion characteristics. Nevertheless, these cells displayed relatively higher levels of lytic/non-lytic cytokines, including TNF-α, IFN-γ, perforin, and granzyme B. These findings seem to provide support for the hypothesis that KLRG1 mediates a potent protective effects. Cush et al.’s reported similar results; they found that KLRG1+ T cells displayed characteristics of multifunctional effector cells with a capacity to kill, despite the persistence of viral lytic antigens during persistent infection with mouse g-herpesvirus 68 (GHV68) [53]. Next, we examined the distinct signs of T-cell maturation and differentiation based on variations in phenotypic traits and effector functions. Interestingly, KLRG1 expression in CD8+ T cells during HBV infection was strongly associated with terminal differentiation into an effector cell. Hence, our findings showed that high KLRG1 expression was associated with the formation of short-term effector cells and long-term memory cells. Specifically, the major KLRG1+ T cells subsets were Teff, SLEC, DPEC, and ML cells, and the first three of these effector cell types produced granzymes, perforin, Fas ligand, and IFN-γ in a strong and consistent manner [54, 55]. In addition, KLRG1 and T-bet were strongly co-expressed, which is essential for promoting T cell growth and maturation. Conversely, TCF-1, which is required to maintain T-cell responses in chronic viral infections [32, 56], was weakly expressed in the KLRG1+ cell populations, resulting in increased differentiation of Teff cell populations. These findings offer a credible explanation for the functional persistence of T cells.

In the present study, we also discovered that lower-risk, inactive patients (HBeAg− CHB) had increased levels of KLRG1 expression in their non-specific CD8+ T cells. Moreover, there was a substantial negative correlation found between KLRG1 and ALT, HBsAg, and HBV DNA levels. These findings imply that enrichment of KLRG1+ T cells may prevent the progression of CHB and align.with the findings of other studies, which have shown that KLRG1+ T cells have positive immunomodulatory effects in chronic infections and cases of human lung or colorectal adenocarcinoma [57–59]. Similarly, PD-1, a classical inhibitory receptor expressed on the cell surface, was shown to be highly cytotoxic in a chronic inflammatory disease even though it significantly paralleled the expression of remaining exhaustion molecules [60].

Although stimulatory and inhibitory molecules exist for many natural killer gene complex (NKC)-encoded C-type lectin-like receptor (CTLR) subgroups [61, 62], KLRG1 has no stimulatory counterpart. The unique and inhibitory ligand of KLRG1 (E-cadherin) is expressed by antigen-presenting cells in peripheral blood or tissues and can exert its effect in vitro by binding to KLRG1 on CD8+ T cells. Hence, we evaluated the sE-cadherin levels in serum samples from CHB patients and E-cadherin in HepG2.2.15 cells. It is interesting to note that sE-cadherin and E-cadherin were expressed to a lesser extent in serum samples from HCs (versus samples from CHB patients) and in HBV-free cells (versus HepG2.2.15 cells), respectively. We then confirmed that the presence of sE-cadherin abrogated the inhibitory effect mediated by HBV-specific CD8+ T cells on HBV replication via cell co-culture experiments. It is worth noting that overexpression of E-cadherin in lysozyme herpes simplex virus (OHSV)-infected cells may improve viral replication by protecting the cells from NK cell-mediated killing [63]. These findings suggest that E-cadherin−KLRG1 binding is critical in HBV-specific CD8+ T cell exhaustion. Overall, HBV infection promotes the expression of E-cadherin, which in turn increases the level of sE-cadherin and reduces T cell function via E-cadherin−KLRG1 binding, resulting in chronic HBV infection. Results that we previously published, which showed that E-cadherin expression decreased with the level of HBc protein [64], also support this notion. Remarkably, it has been shown that the function of exhausted CD8+ T cells can be restored by an ex vivo antibody blockade of KLRG1 [65], providing fresh motivation for future research on immunotherapeutic approaches for treating chronic HBV infection.

## Conclusions

Using multiple experimental techniques, we showed that KLRG1-expressing CD8+ T cells were not completely exhausted but eventually differentiated into functional subpopulations of effector memory cells, unlike HBV-specific CD8+ T cells. KLRG1-expressing CD8+ T cells exhibited upregulation of inhibitory markers; however, their antiviral function was unaffected, presumably because the inhibitory signaling was insufficient. In cHBV infection, upregulation of E-cadherin in infected liver cells and sE-cadherin in the plasma activates the inhibitory KLRG1 pathway in HBV-specific CD8+ T cells, which results in persistent infection. The findings of this study provide insight into the role KLRG1 plays in HBV infection and indicate that its expression and binding to E-cadherin may serve as potential targets in future immunotherapeutic treatments for HBV infection.

## Supporting information

S1 Fig Gating strategy used to detect KLRG1+ CD8+ T cells in patients with chronic HBV. PBMCs from patients with chronic HBV were stained with anti-CD3, anti-CD8, anti-CD45RA, anti-CD127, anti-CCR7, anti-PD-1, anti-KLRG1, anti-CD69, anti-TIM-3, and anti-Lag-3 antibodies.
(TIF)

S2 Fig Distribution of T cells and CD8+ T cells in patients with chronic hepatitis B. The %T cells and %CD8+ T cells values are shown according to a single indicator **(a)** or different phases of CHB **(b).**
(TIF)

S3 Fig Distribution of surface markers and transcription factors in patients with chronic hepatitis B. Expression of PD-1, Lag-3, TIM-3, CD69, T-bet, EOMES, Helios, TOX, and TCF-1 across the four phases of CHB in the KLRG1+ and KLRG1− subgroups.
(TIF)

S4 Fig Distribution of the expression of transcription factors at the mRNA level in patients with chronic hepatitis B. Expression of T-bet, EOMES, Helios, TOX, and TCF-1 was assessed in KLRG1+ and KLRG1− CD8+ T cells at the mRNA level.
(TIF)

S5 Fig Serum sE-cadherin levels in a healthy control and patients with CHB. Serum sE-cadherin levels are shown according to CHB phase and in a healthy control.
(TIF)

S1 Table List of antibodies used in this study. (DOCX)

S2 Table Genes upregulated in KLRG1+ versus KLRG1− CD8 T cells from CHB patients. (DOCX)

S3 Table Genes downregulated in KLRG1+ versus KLRG1− CD8 T cells from CHB patients. (DOCX)

S4 Table Specific primers used in this study. (DOCX)

S1 File Supplementary materials and methods. (DOCX)

S1 Raw images (PDF)
