# Supplementary figures and images for: KLRG1-expressing CD8+ T cells are exhausted and polyfunctional in patients with chronic hepatitis B
Source: PLoS One. 2024 May 22;19(5):e0303945. doi: 10.1371/journal.pone.0303945 (PMC11111010; doi:10.1371/journal.pone.0303945)

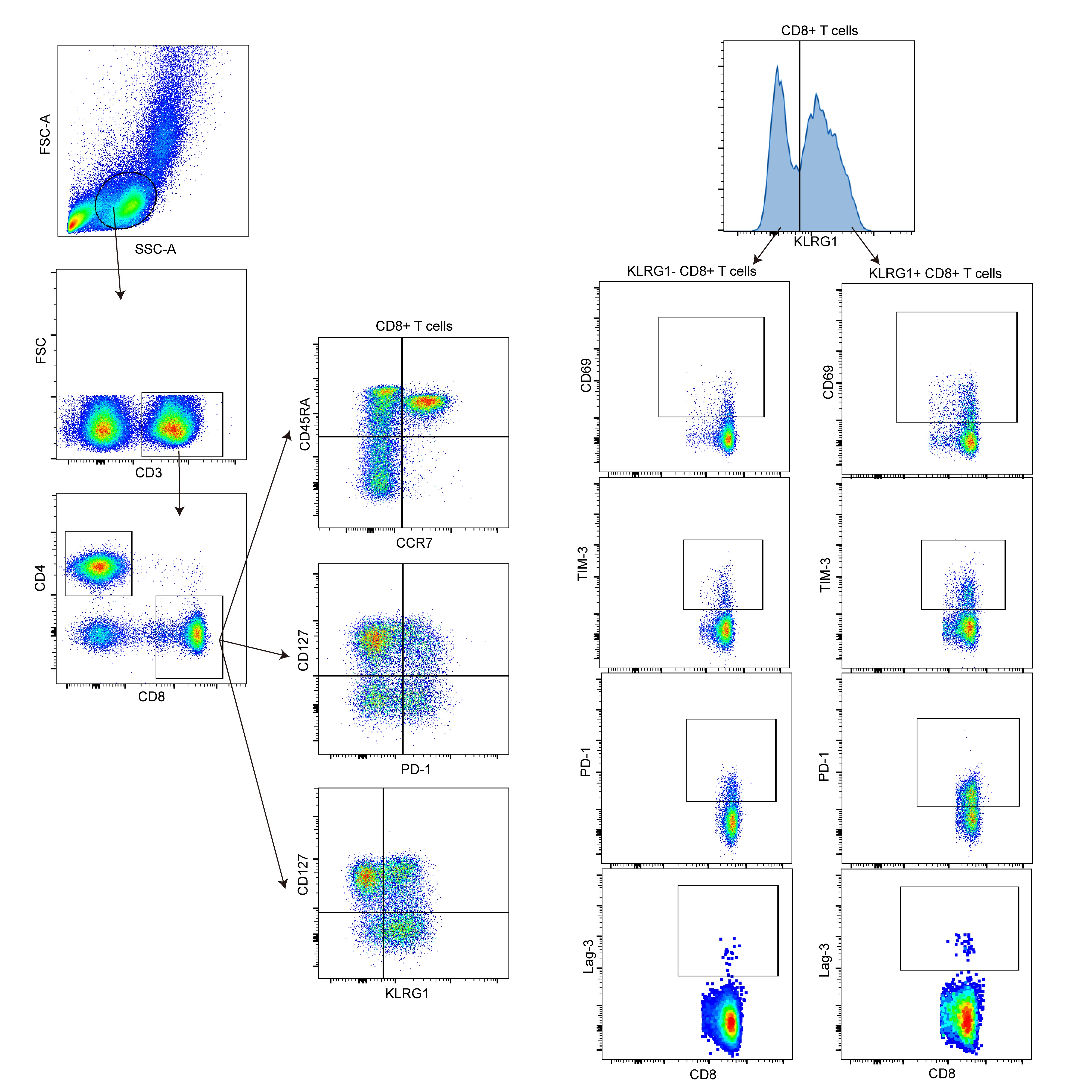

Supplement: S1 Fig — PBMCs from patients with chronic HBV were stained with anti-CD3, anti-CD8, anti-CD45RA, anti-CD127, anti-CCR7, anti-PD-1, anti-KLRG1, anti-CD69, anti-TIM-3, and anti-Lag-3 antibodies. (TIF) [file pone.0303945.s001.tif]

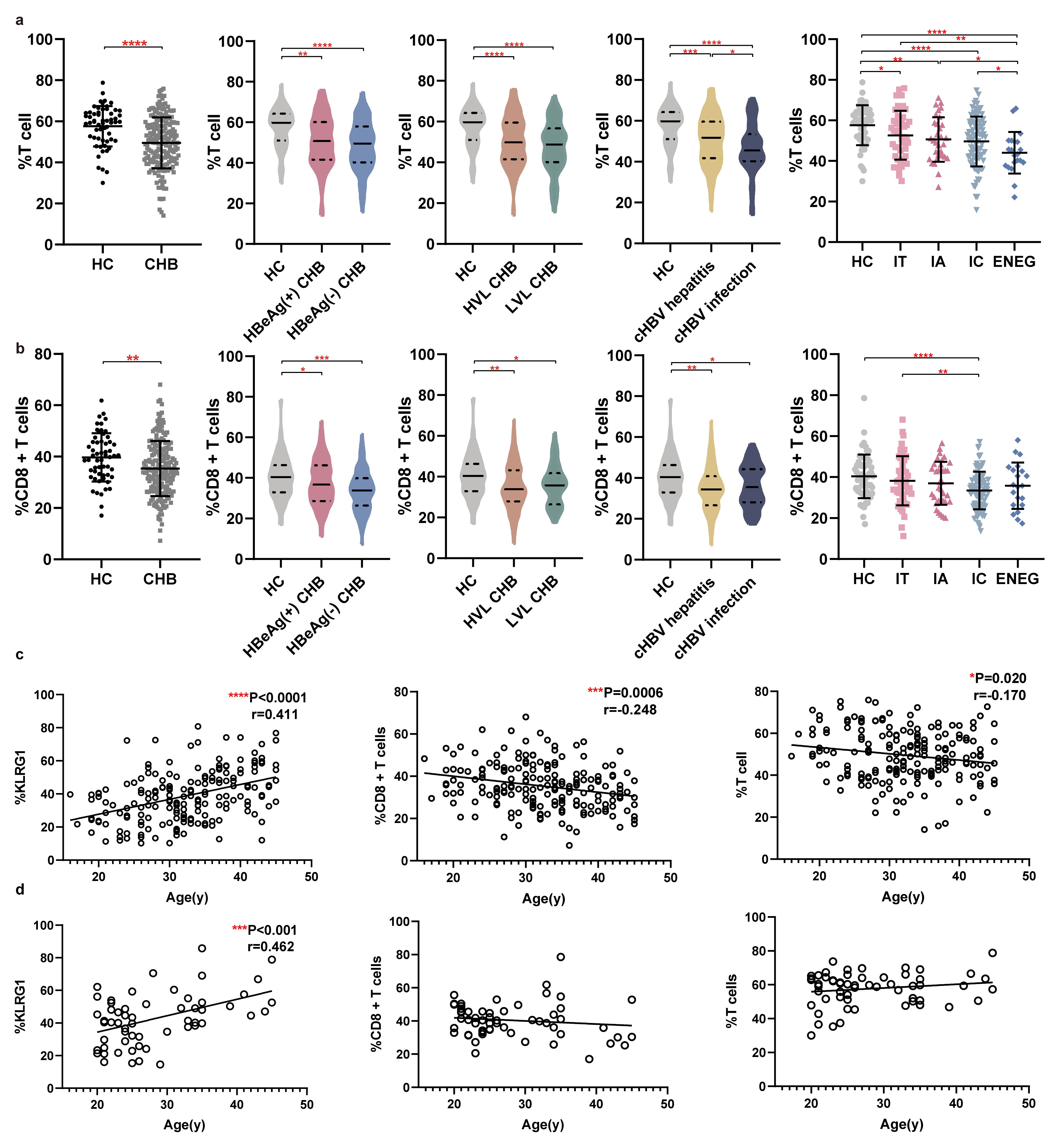

Supplement: S2 Fig — The %T cells and %CD8+ T cells values are shown according to a single indicator (a) or different phases of CHB (b). (TIF) [file pone.0303945.s002.tif]

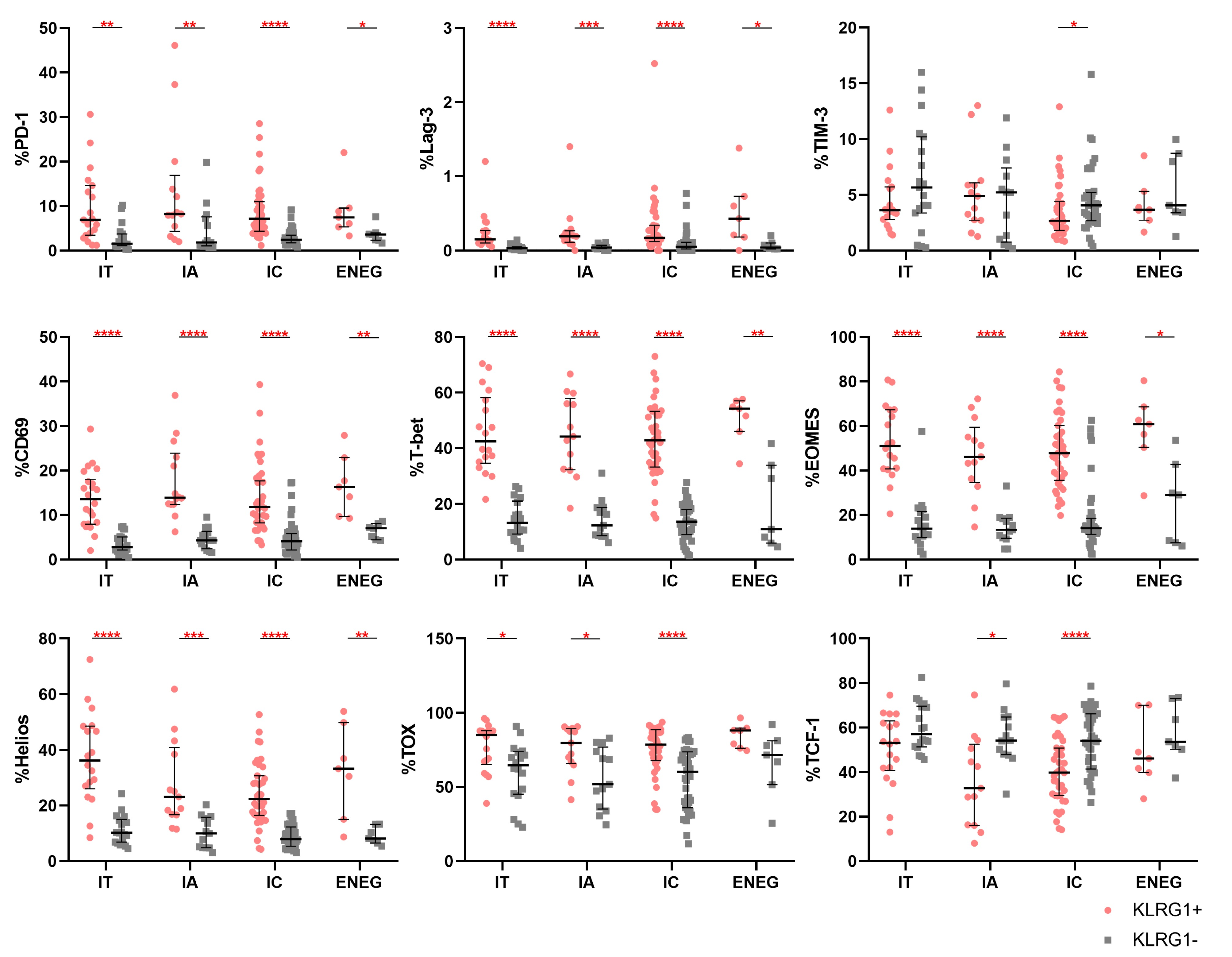

Supplement: S3 Fig — Expression of PD-1, Lag-3, TIM-3, CD69, T-bet, EOMES, Helios, TOX, and TCF-1 across the four phases of CHB in the KLRG1+ and KLRG1− subgroups. (TIF) [file pone.0303945.s003.tif]

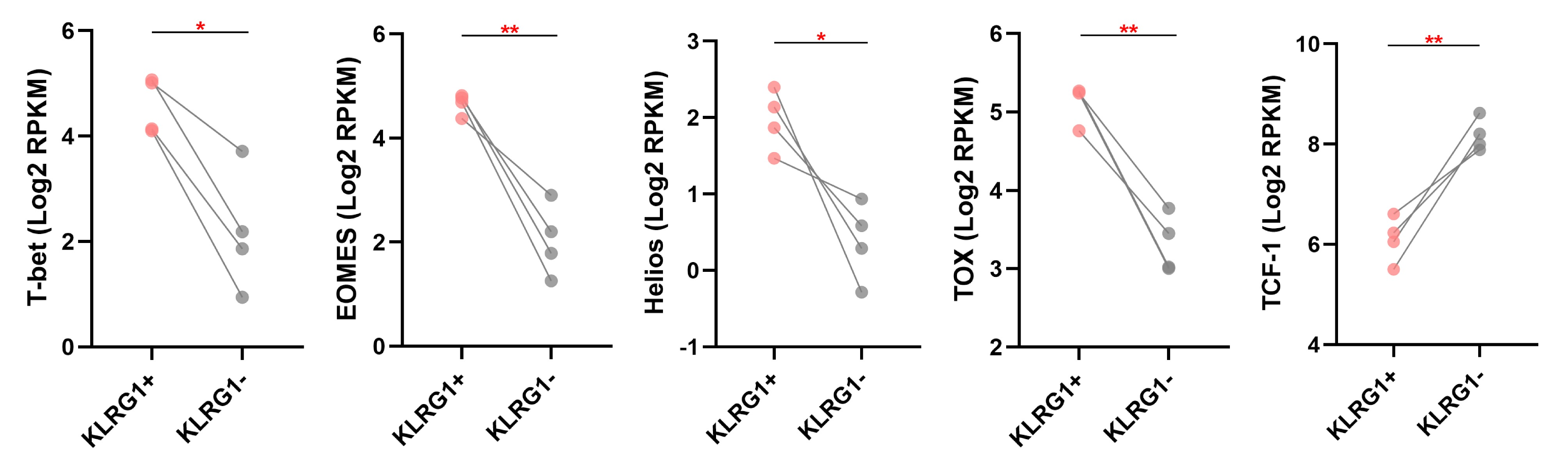

Supplement: S4 Fig — Expression of T-bet, EOMES, Helios, TOX, and TCF-1 was assessed in KLRG1+ and KLRG1− CD8+ T cells at the mRNA level. (TIF) [file pone.0303945.s004.tif]

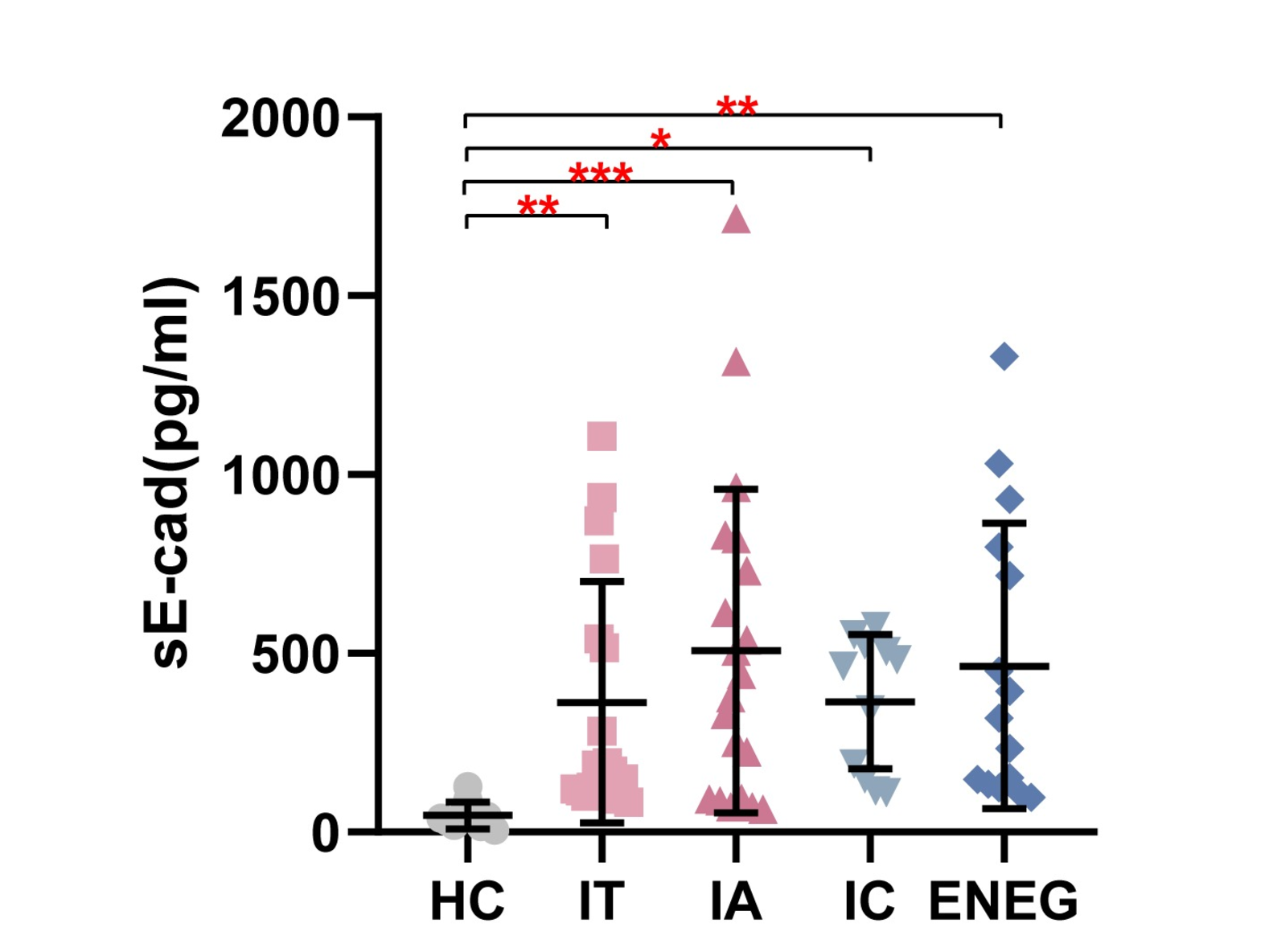

Supplement: S5 Fig — Serum sE-cadherin levels are shown according to CHB phase and in a healthy control. (TIF) [file pone.0303945.s005.tif]

**Fig 7A**

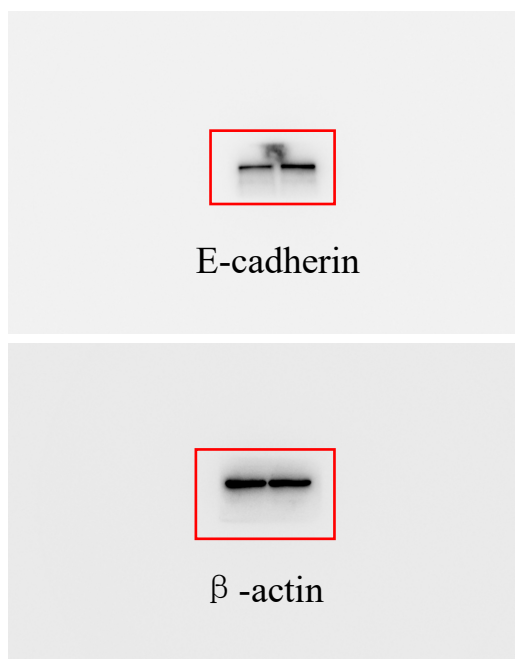

**Fig 7D**

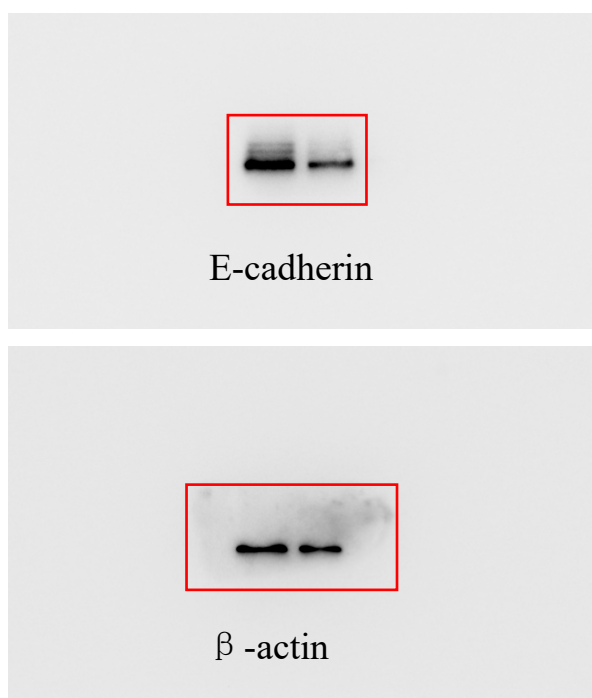

**Fig 7E**

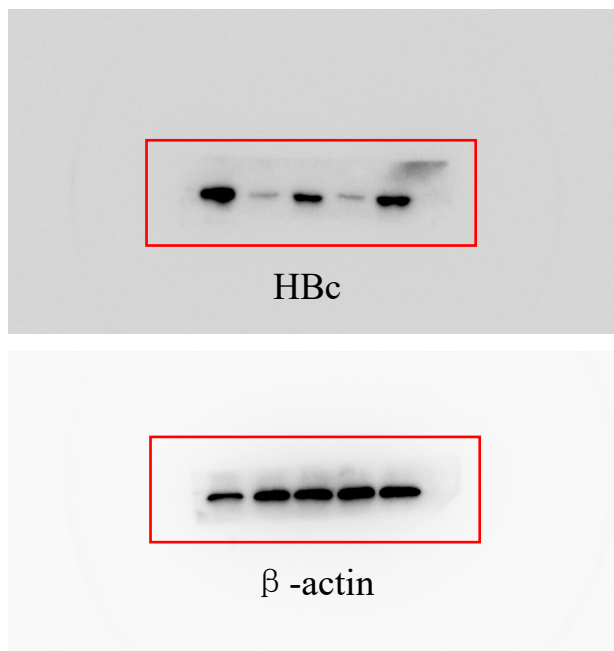

Supplement: S1 Raw images — (PDF) [file pone.0303945.s011.pdf]
