# Supplementary material for: KLRG1-expressing CD8+ T cells are exhausted and polyfunctional in patients with chronic hepatitis B
Source: PLoS One. 2024 May 22;19(5):e0303945. doi: 10.1371/journal.pone.0303945 (PMC11111010; doi:10.1371/journal.pone.0303945)
Supplement: S1 Table — (DOCX) [file pone.0303945.s006.docx]

**KLRG1-expressing CD8+ T cells are exhausted and polyfunctional in patients with chronic hepatitis B.**

S1 Table. List of antibodies used in this study.

| Antigen | Fluorochrome | Manufacturer | Clone | Catolog number |
| --- | --- | --- | --- | --- |
| CD8 | FITC | Biolegend | HIT8a | 300906 |
| CCR7 | PE | BD Biosciences | 3D12 | 561008 |
| CD127 | PE-CF594 | BD Biosciences | HIL-7R-M21 | 562397 |
| CD3 | Percp-cy5.5 | Biolegend | UCHT1 | 300430 |
| CD45RA | PE-Cy7 | BD Biosciences | HI100 | 560675 |
| KLRG1 | APC | Biolegend | 14C2A07 | 368606 |
| CD4 | APC-Cy7 | Biolegend | RPA-T4 | 300518 |
| CD69 | BV421 | BD Biosciences | FN50 | 562884 |
| PD-1 | BV605 | BD Biosciences | EH12.1 | 563245 |
| TIM-3 | PE-CF594 | BD Biosciences | 7D3 | 565561 |
| KLRG1 | PE-Cy7 | Biolegend | 14C2A07 | 368614 |
| Lag-3 | BV421 | Biolegend | 11C3C65 | 369314 |
| TCF-1 | PE | Biolegend | 7F11A10 | 655207 |
| Helios | PE-CF594 | Biolegend | 22F6 | 137231 |
| Eomes | PE-Cy7 | eBioscience | WD1928 | 25-4877-42 |
| TOX | APC | eBioscience | TXRX10 | 50-6502-82 |
| T-bet | BV421 | Biolegend | 4B10 | 644815 |
| KLRG1 | BV785 | Biolegend | 2F1/KLRG1 | 138429 |
| IL-2 | PE-CF594 | Biolegend | MQ1-17H12 | 500343 |
| TNF-α | PE-Cy7 | BD Biosciences | MAb11 | 960923 |
| IFN-γ | BV421 | BD Biosciences | B27 | 562988 |
| Perforin | PE-CF594 | BD Biosciences | G9 | 563763 |
| Granzyme B | BV421 | BD Biosciences | GB11 | 563389 |
| HBc18-27 | PE | Immudex | FLPSDFFPSV | WB03289 |
