# Supplementary material for: KLRG1-expressing CD8+ T cells are exhausted and polyfunctional in patients with chronic hepatitis B
Source: PLoS One. 2024 May 22;19(5):e0303945. doi: 10.1371/journal.pone.0303945 (PMC11111010; doi:10.1371/journal.pone.0303945)
Supplement: S2 Table — (DOCX) [file pone.0303945.s007.docx]

**KLRG1-expressing CD8+ T cells are exhausted and polyfunctional in patients with chronic hepatitis B.**

S2 Table. Genes upregulated in KLRG1+ vs. KLRG1- CD8 T cells from CHB patients.

| Gene ID | Gene Symbol | log2FoldChange | Pvalue | Qvalue |
| --- | --- | --- | --- | --- |
| 100 | ADA | 1.08574684 | 0.001438604 | 0.011648561 |
| 1000 | CDH2 | 4.792303736 | 2.27E-04 | 0.00250315 |
| 10019 | SH2B3 | 1.07710777 | 5.81E-12 | 3.65E-10 |
| 100191040 | C2CD4D | 4.329529164 | 0.016506232 | 0.080802708 |
| 100505753 | KRTAP16-1 | 1.800405938 | 7.32E-07 | 1.66E-05 |
| 100526771 | SMIM35 | 1.161871613 | 6.82E-04 | 0.006219411 |
| 10071 | MUC12 | 2.78401308 | 3.33E-05 | 4.78E-04 |
| 10076 | PTPRU | 3.622714312 | 0.036723636 | 0.148429448 |
| 10079 | ATP9A | 2.697038244 | 2.72E-07 | 6.87E-06 |
| 100820829 | MYZAP | 5.132198575 | 0.012574534 | 0.06541531 |
| 10090 | UST | 1.017423707 | 0.003903197 | 0.026046452 |
| 10098 | TSPAN5 | 1.231827424 | 0.002191449 | 0.016541432 |
| 101 | ADAM8 | 1.694483191 | 1.57E-09 | 6.47E-08 |
| 10100 | TSPAN2 | 1.476845511 | 1.32E-04 | 0.001583311 |
| 10129 | FRY | 1.129439919 | 0.04288456 | 0.165486142 |
| 10130 | PDIA6 | 1.294165128 | 1.06E-11 | 6.25E-10 |
| 10133 | OPTN | 1.090074495 | 5.83E-12 | 3.65E-10 |
| 101410538 | MMP24OS | 1.276698701 | 3.90E-04 | 0.003935669 |
| 10219 | KLRG1 | 2.971276929 | 4.87E-19 | 1.01E-16 |
| 10253 | SPRY2 | 1.931800325 | 2.88E-04 | 0.003044184 |
| 10267 | RAMP1 | 2.961323788 | 0.001360695 | 0.011118802 |
| 102724485 | LOC102724485 | 1.21037182 | 2.04E-04 | 0.002290315 |
| 1028 | CDKN1C | 1.963316107 | 0.037351265 | 0.150427287 |
| 1029 | CDKN2A | 2.004525454 | 2.08E-07 | 5.40E-06 |
| 1030 | CDKN2B | 2.023564251 | 0.005487435 | 0.03416625 |
| 10312 | TCIRG1 | 1.064886652 | 3.24E-05 | 4.66E-04 |
| 10316 | NMUR1 | 2.112352564 | 1.85E-05 | 2.90E-04 |
| 10361 | NPM2 | 3.037377835 | 0.001359854 | 0.011117303 |
| 10382 | TUBB4A | 1.503557928 | 6.61E-06 | 1.15E-04 |
| 10417 | SPON2 | 1.813688674 | 0.002094222 | 0.015949831 |
| 10418 | SPON1 | 1.760718877 | 6.61E-08 | 1.96E-06 |
| 10451 | VAV3 | 1.996057024 | 1.89E-21 | 4.91E-19 |
| 10459 | MAD2L2 | 1.074218863 | 7.84E-07 | 1.76E-05 |
| 10477 | UBE2E3 | 1.008226201 | 8.92E-10 | 3.84E-08 |
| 1050 | CEBPA | 2.142157804 | 2.84E-05 | 4.15E-04 |
| 10509 | SEMA4B | 1.137238373 | 0.001426134 | 0.011564194 |
| 1052 | CEBPD | 4.923712796 | 1.44E-06 | 2.98E-05 |
| 105369914 | LOC105369914 | 2.206832549 | 0.015815011 | 0.078250227 |
| 10538 | BATF | 1.367925912 | 4.53E-04 | 0.004468218 |
| 10578 | GNLY | 2.176618385 | 2.48E-04 | 0.002678307 |
| 10602 | CDC42EP3 | 1.410618592 | 1.68E-09 | 6.83E-08 |
| 10608 | MXD4 | 1.156741656 | 1.41E-05 | 2.27E-04 |
| 10612 | TRIM3 | 1.135085208 | 2.53E-04 | 0.002713985 |
| 10663 | CXCR6 | 2.981938386 | 3.11E-19 | 6.51E-17 |
| 10673 | TNFSF13B | 1.267046795 | 0.00463909 | 0.029873409 |
| 10678 | B3GNT2 | 1.160815017 | 4.72E-07 | 1.13E-05 |
| 106821730 | BUB1B-PAK6 | 3.568009211 | 1.97E-11 | 1.12E-09 |
| 107080644 | CNPY3-GNMT | 2.124672083 | 0.005034967 | 0.031936562 |
| 107181291 | PYDC5 | 2.295904404 | 0.028376217 | 0.122565268 |
| 10745 | PHTF1 | 1.353197596 | 2.00E-05 | 3.10E-04 |
| 1075 | CTSC | 1.572557429 | 1.11E-11 | 6.53E-10 |
| 10783 | NEK6 | 1.295160392 | 5.36E-05 | 7.24E-04 |
| 10786 | SLC17A3 | 2.555626746 | 2.05E-05 | 3.15E-04 |
| 10788 | IQGAP2 | 1.100836495 | 2.01E-07 | 5.25E-06 |
| 107987158 | LOC107987158 | 1.119813345 | 0.002866806 | 0.020398334 |
| 107987423 | LOC107987423 | 1.976788791 | 1.65E-04 | 0.001900878 |
| 10859 | LILRB1 | 2.361901492 | 7.79E-05 | 0.001000472 |
| 10871 | CD300C | 4.801559448 | 0.015704683 | 0.077911056 |
| 10875 | FGL2 | 1.274703053 | 0.003446743 | 0.023557848 |
| 10950 | BTG3 | 1.067183604 | 3.04E-05 | 4.41E-04 |
| 10979 | FERMT2 | 3.421649603 | 0.008267242 | 0.047265722 |
| 1102 | RCBTB2 | 1.432967287 | 2.36E-05 | 3.56E-04 |
| 11021 | RAB35 | 1.029782736 | 7.78E-07 | 1.75E-05 |
| 11033 | ADAP1 | 1.623029873 | 1.09E-08 | 3.77E-07 |
| 11086 | ADAM29 | 3.469075256 | 0.019364825 | 0.091535265 |
| 11098 | PRSS23 | 2.257083409 | 3.57E-08 | 1.12E-06 |
| 11126 | CD160 | 2.614045582 | 0.015543036 | 0.077307816 |
| 11145 | PLAAT3 | 1.760982298 | 3.45E-12 | 2.25E-10 |
| 11186 | RASSF1 | 1.327950902 | 1.50E-11 | 8.68E-10 |
| 11221 | DUSP10 | 1.020502352 | 0.014839053 | 0.074472506 |
| 112268092 | LOC112268092 | 1.176245467 | 0.041551286 | 0.161834849 |
| 1124 | CHN2 | 2.041234093 | 1.78E-04 | 0.002027715 |
| 11270 | NRM | 1.588105951 | 9.35E-05 | 0.001169728 |
| 1130 | LYST | 1.018867536 | 3.54E-11 | 1.91E-09 |
| 113000 | RPUSD1 | 1.349961467 | 0.002313312 | 0.017245844 |
| 11314 | CD300A | 1.884281609 | 4.71E-09 | 1.76E-07 |
| 113230 | MISP3 | 2.148968757 | 0.006280297 | 0.038102351 |
| 113540 | CMTM1 | 2.089857811 | 0.002874737 | 0.020423667 |
| 114118903 | ARHGAP11A-SCG5 | 2.376591512 | 0.030614647 | 0.129606391 |
| 1145 | CHRNE | 1.639312943 | 0.019015386 | 0.090362641 |
| 114787 | GPRIN1 | 3.27920479 | 0.023548955 | 0.106385203 |
| 114801 | TMEM200A | 1.261241138 | 0.022835896 | 0.103912844 |
| 114827 | FHAD1 | 2.761574248 | 4.01E-04 | 0.004022693 |
| 114879 | OSBPL5 | 1.312864368 | 5.22E-05 | 7.10E-04 |
| 114881 | OSBPL7 | 1.105940381 | 2.02E-06 | 4.02E-05 |
| 115 | ADCY9 | 2.081311942 | 9.93E-12 | 5.89E-10 |
| 115004 | CGAS | 1.31059792 | 3.42E-08 | 1.08E-06 |
| 115352 | FCRL3 | 2.21285819 | 0.002203793 | 0.016582833 |
| 115362 | GBP5 | 1.414367298 | 2.90E-13 | 2.35E-11 |
| 115548 | FCHO2 | 1.962535631 | 2.57E-06 | 4.97E-05 |
| 116039 | OSR2 | 2.184011931 | 0.009923837 | 0.054617297 |
| 116113 | FOXP4 | 1.756255185 | 8.34E-08 | 2.42E-06 |
| 116173 | CMTM5 | 4.610312272 | 0.003397772 | 0.023287996 |
| 116211 | TM4SF19 | 2.325167809 | 1.35E-04 | 0.001605211 |
| 116236 | ABHD15 | 1.263014092 | 4.67E-07 | 1.13E-05 |
| 116496 | NIBAN1 | 1.690518359 | 5.99E-14 | 5.60E-12 |
| 116832 | RPL39L | 1.486662987 | 3.10E-05 | 4.50E-04 |
| 116984 | ARAP2 | 1.277576507 | 4.65E-09 | 1.74E-07 |
| 116987 | AGAP1 | 2.051031932 | 1.23E-14 | 1.27E-12 |
| 117144 | CATSPER1 | 3.97622622 | 0.036554827 | 0.147787405 |
| 117157 | SH2D1B | 1.885122745 | 3.23E-04 | 0.003371262 |
| 117178 | SSX2IP | 1.045238595 | 4.71E-04 | 0.004597215 |
| 118429 | ANTXR2 | 1.002528668 | 0.001278892 | 0.010569153 |
| 1186 | CLCN7 | 1.170751711 | 0.007534756 | 0.043938898 |
| 118788 | PIK3AP1 | 2.244037742 | 1.08E-14 | 1.14E-12 |
| 118881 | COMTD1 | 1.018579068 | 0.048809283 | 0.182219581 |
| 1192 | CLIC1 | 1.567192969 | 1.24E-06 | 2.65E-05 |
| 121260 | SLC15A4 | 1.788169028 | 1.01E-08 | 3.53E-07 |
| 121268 | RHEBL1 | 2.105588873 | 7.65E-09 | 2.73E-07 |
| 1230 | CCR1 | 5.140848722 | 4.32E-05 | 6.00E-04 |
| 1234 | CCR5 | 2.315809173 | 1.05E-10 | 5.28E-09 |
| 1235 | CCR6 | 2.008661624 | 1.18E-05 | 1.94E-04 |
| 1240 | CMKLR1 | 2.695134593 | 8.82E-04 | 0.007694719 |
| 124599 | CD300LB | 3.845661443 | 0.004247796 | 0.027938383 |
| 124602 | KIF19 | 2.157627461 | 5.73E-06 | 1.01E-04 |
| 124995 | MRPL10 | 1.533561788 | 5.78E-08 | 1.74E-06 |
| 125875 | CLDND2 | 1.945135056 | 1.25E-04 | 0.001502174 |
| 126014 | OSCAR | 2.567149701 | 0.004507892 | 0.029217564 |
| 126410 | CYP4F22 | 2.049667737 | 3.57E-05 | 5.07E-04 |
| 126969 | SLC44A3 | 4.67691148 | 0.005548946 | 0.03448579 |
| 127435 | PODN | 2.074514964 | 0.013728353 | 0.069788201 |
| 127544 | RNF19B | 1.0976513 | 1.10E-04 | 0.001347459 |
| 127687 | C1orf122 | 1.200079827 | 0.003716723 | 0.025068991 |
| 127703 | C1orf216 | 1.268649069 | 4.34E-04 | 0.004296153 |
| 127733 | UBXN10 | 2.314330566 | 4.41E-10 | 2.00E-08 |
| 127829 | ARL8A | 1.094580739 | 0.002363502 | 0.017527407 |
| 127943 | FCRLB | 1.808286657 | 0.009774944 | 0.053971088 |
| 128178 | EDARADD | 1.576011825 | 1.13E-06 | 2.44E-05 |
| 1285 | COL4A3 | 2.048972267 | 0.017633043 | 0.08535583 |
| 1289 | COL5A1 | 3.597124306 | 1.40E-07 | 3.84E-06 |
| 1292 | COL6A2 | 1.304659934 | 1.85E-06 | 3.74E-05 |
| 129807 | NEU4 | 1.964033123 | 0.002983769 | 0.020995563 |
| 130589 | GALM | 1.158574347 | 9.33E-06 | 1.56E-04 |
| 130612 | TMEM198 | 3.5281328 | 0.026160989 | 0.115322327 |
| 131540 | ZDHHC19 | 4.011275984 | 0.049613527 | 0.184327051 |
| 131583 | FAM43A | 2.620210392 | 1.65E-05 | 2.62E-04 |
| 132014 | IL17RE | 2.714510667 | 0.014906373 | 0.07476602 |
| 132332 | SMIM43 | 3.269898271 | 0.039613106 | 0.156498253 |
| 1326 | MAP3K8 | 2.172977789 | 1.52E-10 | 7.50E-09 |
| 132864 | CPEB2 | 1.254540689 | 1.54E-05 | 2.47E-04 |
| 132884 | EVC2 | 1.924980842 | 0.01116918 | 0.059664151 |
| 134285 | TMEM171 | 3.881743618 | 9.08E-09 | 3.20E-07 |
| 135458 | HUS1B | 1.112590678 | 0.049365535 | 0.183591449 |
| 1362 | CPD | 1.163994569 | 9.39E-08 | 2.68E-06 |
| 138151 | NACC2 | 1.000968184 | 0.001890037 | 0.014672065 |
| 138428 | PTRH1 | 1.408123184 | 0.001250499 | 0.01037871 |
| 139105 | BEND2 | 1.698366791 | 0.024368791 | 0.109243194 |
| 139189 | DGKK | 2.935723104 | 1.62E-04 | 0.00187394 |
| 1396 | CRIP1 | 1.058227692 | 1.13E-04 | 0.001379911 |
| 139716 | GAB3 | 1.888409751 | 8.70E-11 | 4.45E-09 |
| 139886 | SPIN4 | 1.336125432 | 1.27E-06 | 2.70E-05 |
| 140469 | MYO3B | 2.614046597 | 1.46E-04 | 0.001722458 |
| 140578 | CHODL | 2.308650295 | 1.48E-04 | 0.001747922 |
| 140766 | ADAMTS14 | 4.554258723 | 0.009013444 | 0.050671039 |
| 140809 | SRXN1 | 2.026971456 | 2.80E-08 | 8.87E-07 |
| 143689 | PIWIL4 | 1.27356867 | 0.049292627 | 0.183457033 |
| 144097 | SPINDOC | 3.508640042 | 0.003938764 | 0.026252738 |
| 144165 | PRICKLE1 | 1.725272685 | 0.045161762 | 0.171901148 |
| 144402 | CPNE8 | 1.64411175 | 1.91E-07 | 4.98E-06 |
| 144811 | LACC1 | 1.548446576 | 0.028734731 | 0.123734824 |
| 145376 | PPP1R36 | 4.391115697 | 0.004861904 | 0.031053806 |
| 146850 | PIK3R6 | 1.674789988 | 2.76E-05 | 4.06E-04 |
| 147040 | KCTD11 | 1.749075006 | 1.23E-04 | 0.001486358 |
| 1471 | CST3 | 2.488494568 | 7.04E-05 | 9.18E-04 |
| 147646 | C19orf84 | 3.982493149 | 0.008406143 | 0.047914168 |
| 147746 | HIPK4 | 2.900596244 | 0.027564506 | 0.119885413 |
| 147798 | TMC4 | 2.019684438 | 3.43E-05 | 4.91E-04 |
| 147912 | SIX5 | 2.509725313 | 0.009723288 | 0.053773596 |
| 147968 | CAPN12 | 1.111920095 | 0.011211592 | 0.059815186 |
| 148741 | ANKRD35 | 1.990142651 | 0.002623868 | 0.019041044 |
| 1488 | CTBP2 | 1.949363896 | 3.30E-17 | 4.99E-15 |
| 149175 | MANEAL | 1.542414343 | 0.019388274 | 0.091599039 |
| 149233 | IL23R | 3.461977641 | 1.62E-12 | 1.14E-10 |
| 149345 | SHISA4 | 1.989764798 | 0.038616718 | 0.15380613 |
| 149473 | CCDC24 | 1.548000339 | 0.001960105 | 0.015082798 |
| 1495 | CTNNA1 | 2.132285573 | 5.90E-09 | 2.15E-07 |
| 149628 | PYHIN1 | 1.975603058 | 1.29E-12 | 9.27E-11 |
| 150771 | ITPRIPL1 | 1.717628095 | 1.01E-08 | 3.52E-07 |
| 1512 | CTSH | 1.920721631 | 2.77E-08 | 8.82E-07 |
| 151742 | PPM1L | 1.519613354 | 7.07E-04 | 0.006407016 |
| 152007 | GLIPR2 | 1.192416574 | 2.44E-05 | 3.66E-04 |
| 1521 | CTSW | 1.57940449 | 2.92E-17 | 4.45E-15 |
| 152100 | CMC1 | 1.887254387 | 8.74E-17 | 1.22E-14 |
| 1524 | CX3CR1 | 2.019711055 | 0.022166058 | 0.101602439 |
| 152789 | JAKMIP1 | 1.96362659 | 6.64E-07 | 1.53E-05 |
| 153090 | DAB2IP | 4.552734232 | 5.41E-06 | 9.62E-05 |
| 1534 | CYB561 | 1.282300822 | 1.45E-07 | 3.97E-06 |
| 154 | ADRB2 | 2.440958739 | 1.05E-12 | 7.64E-11 |
| 154075 | SAMD3 | 1.23954781 | 6.89E-08 | 2.04E-06 |
| 154141 | MBOAT1 | 1.133817398 | 6.26E-04 | 0.005800415 |
| 1548 | CYP2A6 | 3.674590345 | 0.034880988 | 0.142754767 |
| 157313 | CDCA2 | 1.32261797 | 0.042089058 | 0.16351541 |
| 157506 | RDH10 | 1.131037353 | 0.002623269 | 0.019041044 |
| 158248 | TTC16 | 1.158694191 | 4.15E-06 | 7.67E-05 |
| 1601 | DAB2 | 1.293982094 | 0.010537631 | 0.057046808 |
| 1608 | DGKG | 3.709297117 | 0.022986541 | 0.104401834 |
| 1609 | DGKQ | 1.188446891 | 1.64E-05 | 2.60E-04 |
| 161835 | FSIP1 | 1.751765792 | 5.04E-04 | 0.004841044 |
| 162466 | PHOSPHO1 | 1.435794427 | 0.046629017 | 0.175730574 |
| 162514 | TRPV3 | 2.207294844 | 8.14E-04 | 0.007193744 |
| 162966 | ZNF600 | 1.043867535 | 6.64E-04 | 0.006078471 |
| 162979 | ZNF296 | 1.218735541 | 5.59E-04 | 0.005282704 |
| 164668 | APOBEC3H | 2.067225163 | 6.37E-05 | 8.41E-04 |
| 165186 | TOGARAM2 | 2.303000769 | 3.36E-10 | 1.55E-08 |
| 165679 | SPTSSB | 6.015170821 | 1.74E-04 | 0.001993157 |
| 166793 | ZBTB49 | 1.077930614 | 8.98E-04 | 0.007793774 |
| 167681 | PRSS35 | 6.449630587 | 6.53E-08 | 1.94E-06 |
| 169200 | TMEM64 | 1.216657495 | 2.59E-08 | 8.34E-07 |
| 170371 | TMEM273 | 2.545442059 | 3.99E-08 | 1.24E-06 |
| 170384 | FUT11 | 1.396002455 | 1.87E-06 | 3.77E-05 |
| 170394 | PWWP2B | 1.088649235 | 0.001910856 | 0.014814838 |
| 170463 | SSBP4 | 1.495730733 | 8.52E-08 | 2.46E-06 |
| 170954 | PPP1R18 | 1.075272772 | 0.001542033 | 0.012338448 |
| 1741 | DLG3 | 1.095008678 | 0.002740228 | 0.019691243 |
| 1760 | DMPK | 1.037397871 | 0.012821 | 0.066311057 |
| 1762 | DMWD | 1.123198519 | 2.87E-04 | 0.003036093 |
| 1808 | DPYSL2 | 1.31183703 | 1.47E-09 | 6.12E-08 |
| 1844 | DUSP2 | 1.259365809 | 1.38E-06 | 2.88E-05 |
| 1847 | DUSP5 | 2.077793603 | 7.50E-15 | 8.03E-13 |
| 1850 | DUSP8 | 1.07608174 | 5.68E-04 | 0.005346081 |
| 1908 | EDN3 | 2.631279011 | 0.028867138 | 0.124115491 |
| 192670 | AGO4 | 1.715662555 | 2.79E-13 | 2.27E-11 |
| 1946 | EFNA5 | 2.492337439 | 7.91E-05 | 0.001013536 |
| 196385 | DNAH10 | 2.102922051 | 0.002105076 | 0.016010875 |
| 197135 | PATL2 | 1.378483649 | 1.61E-04 | 0.001866638 |
| 199675 | MCEMP1 | 2.397815252 | 0.010977968 | 0.058977826 |
| 199713 | NLRP7 | 2.399863536 | 0.008112805 | 0.046587374 |
| 2 | A2M | 2.73651829 | 1.34E-23 | 4.37E-21 |
| 20 | ABCA2 | 1.154364862 | 5.17E-05 | 7.04E-04 |
| 201633 | TIGIT | 1.709000115 | 1.57E-08 | 5.28E-07 |
| 203260 | CCDC107 | 1.087624396 | 4.67E-07 | 1.13E-05 |
| 2034 | EPAS1 | 1.982548444 | 0.041982301 | 0.163213067 |
| 203859 | ANO5 | 3.251137113 | 0.04499474 | 0.171496845 |
| 2040 | STOM | 1.656809846 | 1.09E-10 | 5.45E-09 |
| 2043 | EPHA4 | 1.15789267 | 9.15E-04 | 0.007939308 |
| 2060 | EPS15 | 1.008714669 | 2.90E-06 | 5.54E-05 |
| 2081 | ERN1 | 1.872236604 | 2.05E-19 | 4.39E-17 |
| 2100 | ESR2 | 1.033839699 | 0.011569899 | 0.061167574 |
| 2121 | EVC | 4.44898876 | 3.17E-06 | 6.01E-05 |
| 2149 | F2R | 2.337285015 | 2.62E-12 | 1.77E-10 |
| 2157 | F8 | 1.417360283 | 7.07E-05 | 9.20E-04 |
| 2175 | FANCA | 1.305167907 | 2.19E-07 | 5.63E-06 |
| 2180 | ACSL1 | 1.05804376 | 1.52E-07 | 4.10E-06 |
| 219972 | MPEG1 | 1.107522795 | 0.032471984 | 0.135417297 |
| 221002 | RASGEF1A | 1.793100331 | 1.10E-07 | 3.10E-06 |
| 221188 | ADGRG5 | 2.153684718 | 8.54E-04 | 0.007478657 |
| 2213 | FCGR2B | 1.369735763 | 5.45E-06 | 9.68E-05 |
| 2214 | FCGR3A | 2.497953639 | 6.95E-14 | 6.39E-12 |
| 2215 | FCGR3B | 2.265762387 | 0.002023544 | 0.015495219 |
| 221662 | RBM24 | 5.970937315 | 1.28E-06 | 2.71E-05 |
| 221895 | JAZF1 | 1.519193741 | 1.09E-10 | 5.46E-09 |
| 222487 | ADGRG3 | 2.761380913 | 2.55E-05 | 3.81E-04 |
| 2256 | FGF11 | 1.985914108 | 3.94E-04 | 0.003968838 |
| 2263 | FGFR2 | 1.317334001 | 0.002867555 | 0.020398334 |
| 2268 | FGR | 2.288792599 | 3.44E-04 | 0.003551037 |
| 22800 | RRAS2 | 1.117758305 | 8.02E-04 | 0.007113896 |
| 22807 | IKZF2 | 1.622190542 | 7.87E-09 | 2.80E-07 |
| 22822 | PHLDA1 | 1.647615998 | 0.003362398 | 0.023093289 |
| 22829 | NLGN4Y | 2.875471356 | 0.005825995 | 0.035878206 |
| 22836 | RHOBTB3 | 1.900251656 | 2.37E-04 | 0.00258746 |
| 22856 | CHSY1 | 1.209104701 | 4.27E-08 | 1.31E-06 |
| 22875 | ENPP4 | 1.262733937 | 2.73E-06 | 5.26E-05 |
| 22891 | ZNF365 | 1.99354733 | 3.87E-09 | 1.48E-07 |
| 22898 | DENND3 | 2.133114017 | 7.34E-08 | 2.16E-06 |
| 23022 | PALLD | 2.435487635 | 2.39E-09 | 9.43E-08 |
| 23092 | ARHGAP26 | 1.036590758 | 1.82E-07 | 4.79E-06 |
| 23108 | RAP1GAP2 | 1.933452957 | 2.18E-14 | 2.12E-12 |
| 23127 | COLGALT2 | 2.027583871 | 0.020161244 | 0.094204175 |
| 23129 | PLXND1 | 2.93977109 | 2.46E-27 | 1.34E-24 |
| 23130 | ATG2A | 1.363269591 | 7.82E-07 | 1.76E-05 |
| 23139 | MAST2 | 1.263837514 | 1.33E-04 | 0.001591412 |
| 23150 | FRMD4B | 1.56514264 | 2.38E-04 | 0.002600119 |
| 23151 | GRAMD4 | 1.481170138 | 2.27E-04 | 0.00250315 |
| 2316 | FLNA | 1.223041867 | 6.32E-13 | 4.80E-11 |
| 23176 | SEPTIN8 | 1.53381367 | 4.84E-07 | 1.16E-05 |
| 23208 | SYT11 | 1.320965064 | 2.71E-05 | 4.00E-04 |
| 23209 | MLC1 | 2.006702758 | 2.08E-04 | 0.002320278 |
| 23218 | NBEAL2 | 1.647687322 | 1.29E-06 | 2.72E-05 |
| 23224 | SYNE2 | 1.155427317 | 6.73E-14 | 6.23E-12 |
| 23236 | PLCB1 | 1.987881528 | 1.84E-12 | 1.28E-10 |
| 23333 | DPY19L1 | 1.427771886 | 3.63E-08 | 1.13E-06 |
| 23336 | SYNM | 2.791878714 | 0.049656976 | 0.184447983 |
| 23345 | SYNE1 | 1.160844083 | 2.94E-07 | 7.39E-06 |
| 23365 | ARHGEF12 | 1.283764349 | 9.30E-05 | 0.001164385 |
| 23413 | NCS1 | 4.747459841 | 1.69E-04 | 0.001946116 |
| 23415 | KCNH4 | 2.705587928 | 0.039771761 | 0.156889861 |
| 23475 | QPRT | 1.107297036 | 0.015445515 | 0.076900397 |
| 23529 | CLCF1 | 2.525879937 | 2.40E-17 | 3.72E-15 |
| 2355 | FOSL2 | 1.465376064 | 1.83E-07 | 4.81E-06 |
| 23555 | TSPAN15 | 2.726772322 | 3.58E-07 | 8.90E-06 |
| 23564 | DDAH2 | 1.170138108 | 5.91E-05 | 7.85E-04 |
| 23603 | CORO1C | 1.146678198 | 3.58E-04 | 0.003666847 |
| 23705 | CADM1 | 2.364806503 | 0.014118902 | 0.071494366 |
| 23753 | SDF2L1 | 1.110241251 | 5.93E-07 | 1.39E-05 |
| 23764 | MAFF | 1.353447847 | 1.03E-06 | 2.25E-05 |
| 241 | ALOX5AP | 1.182818796 | 3.06E-07 | 7.66E-06 |
| 2519 | FUCA2 | 1.088350454 | 9.06E-06 | 1.52E-04 |
| 253152 | EPHX4 | 2.290737831 | 3.33E-04 | 0.003459976 |
| 2534 | FYN | 1.495198321 | 8.13E-12 | 4.89E-10 |
| 253582 | TMEM244 | 2.746939678 | 0.011875581 | 0.062451975 |
| 254050 | LRRC43 | 2.233284977 | 0.018150564 | 0.087311537 |
| 254263 | CNIH2 | 2.493640405 | 0.01585005 | 0.078315445 |
| 254778 | VXN | 1.458947908 | 5.90E-04 | 0.005522201 |
| 2549 | GAB1 | 2.255667533 | 0.01891901 | 0.090035549 |
| 255231 | MCOLN2 | 1.553230474 | 4.17E-17 | 6.08E-15 |
| 256356 | GK5 | 1.057668189 | 1.40E-06 | 2.91E-05 |
| 256536 | TCERG1L | 3.352806977 | 0.007378745 | 0.043118269 |
| 256949 | KANK3 | 2.058938093 | 0.027129909 | 0.118482437 |
| 256957 | HEATR9 | 1.960385062 | 7.78E-08 | 2.28E-06 |
| 2581 | GALC | 1.273922652 | 1.34E-08 | 4.54E-07 |
| 25819 | NOCT | 1.099991974 | 0.025463584 | 0.112983807 |
| 25861 | WHRN | 1.897690091 | 2.47E-05 | 3.70E-04 |
| 2591 | GALNT3 | 1.933775213 | 2.34E-12 | 1.58E-10 |
| 259197 | NCR3 | 1.902537244 | 2.44E-09 | 9.57E-08 |
| 259307 | IL4I1 | 3.91965934 | 3.48E-12 | 2.27E-10 |
| 25984 | KRT23 | 5.572194346 | 1.60E-04 | 0.00186215 |
| 26000 | TBC1D10B | 1.064903679 | 0.004517225 | 0.029255645 |
| 26030 | PLEKHG3 | 2.151476108 | 0.001540796 | 0.012338448 |
| 26031 | OSBPL3 | 1.360149479 | 1.59E-07 | 4.26E-06 |
| 26040 | SETBP1 | 1.864832947 | 8.32E-09 | 2.95E-07 |
| 26045 | LRRTM2 | 2.043619581 | 0.047098411 | 0.17715505 |
| 26051 | PPP1R16B | 1.338703985 | 1.01E-08 | 3.52E-07 |
| 26053 | AUTS2 | 2.324669149 | 1.48E-10 | 7.33E-09 |
| 26056 | RAB11FIP5 | 1.776996657 | 3.63E-05 | 5.15E-04 |
| 26191 | PTPN22 | 1.43617713 | 8.20E-06 | 1.39E-04 |
| 26262 | TSPAN17 | 1.155132445 | 0.015948218 | 0.078639771 |
| 26267 | FBXO10 | 1.169632491 | 0.004862766 | 0.031053806 |
| 2627 | GATA6 | 1.507489038 | 0.033746403 | 0.139256372 |
| 26469 | PTPN18 | 1.375024197 | 6.78E-04 | 0.006192471 |
| 26472 | PPP1R14B | 1.400796969 | 0.006117928 | 0.037282567 |
| 26524 | LATS2 | 2.731055431 | 1.14E-06 | 2.47E-05 |
| 2672 | GFI1 | 1.70772339 | 5.60E-07 | 1.32E-05 |
| 2678 | GGT1 | 1.07816815 | 1.56E-04 | 0.001817407 |
| 2683 | B4GALT1 | 1.023325319 | 1.55E-04 | 0.001806124 |
| 27036 | SIGLEC7 | 3.655575007 | 1.83E-07 | 4.81E-06 |
| 27077 | B9D1 | 1.645543479 | 0.027158833 | 0.118578157 |
| 27087 | B3GAT1 | 2.3746428 | 0.013154309 | 0.067478138 |
| 27122 | DKK3 | 1.348212127 | 0.022659539 | 0.103431074 |
| 27123 | DKK2 | 2.682041416 | 0.001803052 | 0.014132954 |
| 27132 | CPNE7 | 2.153309826 | 0.001386462 | 0.011285758 |
| 27166 | PRELID1 | 1.054473567 | 5.93E-04 | 0.005547803 |
| 27180 | SIGLEC9 | 2.170293954 | 2.67E-04 | 0.002853665 |
| 27189 | IL17C | 2.14184239 | 2.43E-04 | 0.002632723 |
| 27289 | RND1 | 1.011498749 | 0.025736777 | 0.114016615 |
| 273 | AMPH | 1.378662847 | 0.036186735 | 0.146683958 |
| 27333 | GOLIM4 | 1.708353582 | 1.83E-04 | 0.002079587 |
| 27350 | APOBEC3C | 1.354528109 | 8.74E-06 | 1.47E-04 |
| 2737 | GLI3 | 2.665840912 | 5.30E-04 | 0.005046633 |
| 2752 | GLUL | 1.818313902 | 2.29E-10 | 1.10E-08 |
| 2769 | GNA15 | 2.28127727 | 8.12E-04 | 0.007184692 |
| 2774 | GNAL | 1.113589644 | 4.55E-04 | 0.004478582 |
| 2775 | GNAO1 | 2.435513953 | 1.63E-09 | 6.68E-08 |
| 2793 | GNGT2 | 1.880177154 | 3.00E-07 | 7.52E-06 |
| 28232 | SLCO3A1 | 1.170385134 | 1.38E-10 | 6.83E-09 |
| 283229 | CRACR2B | 2.526047129 | 0.005263255 | 0.033094481 |
| 283643 | TEDC1 | 1.561834659 | 0.003227658 | 0.022349286 |
| 284018 | C17orf58 | 1.435816416 | 0.01051635 | 0.056986295 |
| 284106 | CISD3 | 1.223487597 | 0.001141284 | 0.00962329 |
| 284207 | METRNL | 2.544155714 | 1.71E-21 | 4.53E-19 |
| 2850 | GPR27 | 1.788369405 | 0.011301135 | 0.060160145 |
| 285141 | ERICH2 | 3.56837501 | 0.049163309 | 0.183015986 |
| 285386 | TPRG1 | 2.091792132 | 1.29E-13 | 1.11E-11 |
| 2859 | GPR35 | 1.040012313 | 0.002054405 | 0.015717313 |
| 286144 | TRIQK | 2.954226365 | 7.98E-04 | 0.00708495 |
| 286207 | CFAP157 | 1.092197897 | 0.025343408 | 0.11268694 |
| 2889 | RAPGEF1 | 1.21553447 | 1.25E-10 | 6.22E-09 |
| 2909 | ARHGAP35 | 1.409811079 | 6.06E-08 | 1.82E-06 |
| 29091 | STXBP6 | 2.375171259 | 0.001533301 | 0.01228601 |
| 29109 | FHOD1 | 1.163424953 | 2.42E-04 | 0.002629591 |
| 29110 | TBK1 | 1.421767865 | 2.20E-07 | 5.66E-06 |
| 29126 | CD274 | 1.234302079 | 2.18E-05 | 3.32E-04 |
| 2931 | GSK3A | 1.140449526 | 0.009839729 | 0.054293361 |
| 2975 | GTF3C1 | 1.335787631 | 2.10E-14 | 2.05E-12 |
| 29799 | YPEL1 | 1.661137263 | 1.83E-06 | 3.71E-05 |
| 29893 | PSMC3IP | 1.157017517 | 0.026197989 | 0.115400478 |
| 2992 | GYG1 | 1.533779805 | 7.13E-15 | 7.68E-13 |
| 29941 | PKN3 | 1.155393302 | 0.008686433 | 0.049262902 |
| 29946 | SERTAD3 | 2.058772176 | 3.04E-11 | 1.67E-09 |
| 29950 | SERTAD1 | 1.638405295 | 4.89E-11 | 2.59E-09 |
| 29957 | SLC25A24 | 1.074156016 | 1.87E-04 | 0.002119775 |
| 29967 | LRP12 | 1.546089387 | 0.044410543 | 0.169728913 |
| 29988 | SLC2A8 | 1.932527547 | 4.73E-05 | 6.48E-04 |
| 2999 | GZMH | 2.177259688 | 2.09E-04 | 0.002336292 |
| 29993 | PACSIN1 | 1.531398975 | 0.014828709 | 0.074442668 |
| 30009 | TBX21 | 2.200696082 | 1.30E-06 | 2.73E-05 |
| 3001 | GZMA | 2.43823114 | 1.13E-12 | 8.21E-11 |
| 3002 | GZMB | 2.186049146 | 6.11E-08 | 1.83E-06 |
| 3003 | GZMK | 2.282499383 | 1.33E-09 | 5.59E-08 |
| 301 | ANXA1 | 1.051945766 | 0.002690634 | 0.019400765 |
| 302 | ANXA2 | 1.873896162 | 1.84E-10 | 8.98E-09 |
| 307 | ANXA4 | 1.487434484 | 2.51E-06 | 4.88E-05 |
| 3075 | CFH | 2.125207715 | 8.23E-23 | 2.53E-20 |
| 308 | ANXA5 | 1.097868451 | 5.50E-06 | 9.77E-05 |
| 3108 | HLA-DMA | 1.31092103 | 7.34E-10 | 3.17E-08 |
| 3109 | HLA-DMB | 1.066100293 | 9.48E-07 | 2.09E-05 |
| 3113 | HLA-DPA1 | 1.693914199 | 1.60E-10 | 7.87E-09 |
| 3115 | HLA-DPB1 | 1.348223687 | 3.60E-15 | 4.04E-13 |
| 3117 | HLA-DQA1 | 2.215306435 | 3.43E-08 | 1.08E-06 |
| 3119 | HLA-DQB1 | 1.811456453 | 3.10E-10 | 1.45E-08 |
| 3122 | HLA-DRA | 1.474655091 | 7.53E-19 | 1.52E-16 |
| 3123 | HLA-DRB1 | 1.868653487 | 1.98E-06 | 3.95E-05 |
| 3131 | HLF | 4.506467047 | 0.002244861 | 0.01683944 |
| 3162 | HMOX1 | 1.873477633 | 0.045220293 | 0.17208523 |
| 317 | APAF1 | 1.118519272 | 1.23E-04 | 0.001488644 |
| 3198 | HOXA1 | 2.202768299 | 0.001399755 | 0.011377546 |
| 3310 | HSPA6 | 1.956361225 | 0.039686597 | 0.156626932 |
| 333929 | SNAI3 | 1.244517313 | 3.26E-06 | 6.16E-05 |
| 334 | APLP2 | 1.178580592 | 2.35E-11 | 1.33E-09 |
| 3340 | NDST1 | 1.793334089 | 0.028325402 | 0.122465232 |
| 3383 | ICAM1 | 2.026163627 | 0.002325802 | 0.017308511 |
| 338596 | ST8SIA6 | 2.699718134 | 0.004310775 | 0.028210068 |
| 339745 | SPOPL | 1.321311952 | 2.47E-07 | 6.31E-06 |
| 340527 | NHSL2 | 1.700144258 | 0.010525415 | 0.057017158 |
| 3433 | IFIT2 | 1.400274004 | 0.002119192 | 0.016096536 |
| 343413 | FCRL6 | 2.250239411 | 7.20E-04 | 0.006493206 |
| 3437 | IFIT3 | 1.128159225 | 0.008684193 | 0.049262902 |
| 3458 | IFNG | 2.177902398 | 2.00E-05 | 3.10E-04 |
| 3459 | IFNGR1 | 1.536614403 | 1.65E-07 | 4.39E-06 |
| 347404 | LANCL3 | 1.449493084 | 0.02022628 | 0.094372578 |
| 348094 | ANKDD1A | 1.157645197 | 0.001056562 | 0.008982599 |
| 3486 | IGFBP3 | 1.159442076 | 0.019094612 | 0.09064183 |
| 3487 | IGFBP4 | 2.975572666 | 3.97E-11 | 2.14E-09 |
| 353189 | SLCO4C1 | 1.794150745 | 2.28E-05 | 3.46E-04 |
| 353345 | GPR141 | 2.196865925 | 5.70E-07 | 1.34E-05 |
| 355 | FAS | 1.472698922 | 1.61E-12 | 1.14E-10 |
| 356 | FASLG | 2.279242873 | 5.19E-06 | 9.30E-05 |
| 3560 | IL2RB | 1.255333394 | 1.02E-05 | 1.69E-04 |
| 3574 | IL7 | 1.875631625 | 2.25E-04 | 0.0024808 |
| 3579 | CXCR2 | 2.440337432 | 2.42E-06 | 4.73E-05 |
| 3592 | IL12A | 1.15346335 | 3.02E-05 | 4.39E-04 |
| 3594 | IL12RB1 | 1.3058554 | 1.88E-08 | 6.23E-07 |
| 3595 | IL12RB2 | 1.972375326 | 5.49E-07 | 1.30E-05 |
| 3600 | IL15 | 1.827361226 | 1.50E-07 | 4.07E-06 |
| 3601 | IL15RA | 1.503203599 | 4.69E-05 | 6.44E-04 |
| 3604 | TNFRSF9 | 1.729810475 | 6.79E-04 | 0.006201015 |
| 3628 | INPP1 | 1.039537529 | 0.001642607 | 0.013020062 |
| 3663 | IRF5 | 1.597403887 | 6.54E-04 | 0.006007615 |
| 3681 | ITGAD | 3.845638501 | 7.78E-04 | 0.006941071 |
| 3683 | ITGAL | 1.255446652 | 4.19E-11 | 2.25E-09 |
| 3684 | ITGAM | 2.192485084 | 5.69E-07 | 1.34E-05 |
| 3685 | ITGAV | 1.002882098 | 6.92E-05 | 9.05E-04 |
| 3688 | ITGB1 | 1.09048888 | 1.93E-04 | 0.002170546 |
| 3689 | ITGB2 | 1.071896892 | 1.32E-05 | 2.15E-04 |
| 3742 | KCNA6 | 2.205013914 | 0.007624423 | 0.044370053 |
| 374907 | B3GNT8 | 2.894535498 | 6.40E-09 | 2.31E-07 |
| 375033 | PEAR1 | 2.915742231 | 0.041192164 | 0.160955904 |
| 375387 | NRROS | 2.183147945 | 2.49E-04 | 0.002680154 |
| 375616 | KCP | 1.945091836 | 0.007643466 | 0.0444503 |
| 3786 | KCNQ3 | 5.201266063 | 0.002286005 | 0.01707994 |
| 3797 | KIF3C | 1.074896036 | 3.81E-04 | 0.003859655 |
| 3800 | KIF5C | 1.552656224 | 4.39E-05 | 6.09E-04 |
| 3801 | KIFC3 | 3.173005755 | 1.06E-09 | 4.51E-08 |
| 3802 | KIR2DL1 | 2.215781376 | 0.012203229 | 0.063778641 |
| 3804 | KIR2DL3 | 2.031233417 | 1.91E-04 | 0.002160987 |
| 3806 | KIR2DS1 | 3.223218137 | 0.043802076 | 0.168048634 |
| 3809 | KIR2DS4 | 2.915376066 | 6.05E-12 | 3.78E-10 |
| 3811 | KIR3DL1 | 2.422936174 | 0.013519188 | 0.06900654 |
| 3812 | KIR3DL2 | 2.697185897 | 5.85E-10 | 2.59E-08 |
| 3820 | KLRB1 | 3.265762076 | 1.25E-15 | 1.49E-13 |
| 3822 | KLRC2 | 1.309540358 | 8.39E-04 | 0.007378254 |
| 3824 | KLRD1 | 2.044543587 | 0.001250419 | 0.01037871 |
| 387509 | GPR153 | 4.847780318 | 4.54E-10 | 2.05E-08 |
| 387522 | PEDS1-UBE2V1 | 1.38675238 | 0.003158022 | 0.021974999 |
| 387882 | C12orf75 | 1.808377595 | 6.20E-10 | 2.72E-08 |
| 388372 | CCL4L1 | 2.681167934 | 3.55E-25 | 1.47E-22 |
| 388677 | NOTCH2NLA | 1.322826553 | 0.001725344 | 0.013580461 |
| 389 | RHOC | 2.428268123 | 2.64E-16 | 3.54E-14 |
| 389197 | C4orf50 | 2.536901634 | 4.91E-06 | 8.87E-05 |
| 3892 | KRT86 | 3.718553186 | 6.95E-07 | 1.59E-05 |
| 389813 | AJM1 | 2.188143523 | 2.17E-05 | 3.31E-04 |
| 3902 | LAG3 | 2.294341187 | 6.15E-12 | 3.81E-10 |
| 3904 | LAIR2 | 2.630264439 | 9.09E-09 | 3.20E-07 |
| 3908 | LAMA2 | 3.937674494 | 0.012843971 | 0.066389285 |
| 391013 | PLA2G2C | 3.893009654 | 0.048764524 | 0.182132431 |
| 3914 | LAMB3 | 3.995728766 | 0.013321906 | 0.068229673 |
| 3949 | LDLR | 1.130410168 | 2.40E-04 | 0.002616809 |
| 3956 | LGALS1 | 1.661158628 | 5.55E-06 | 9.82E-05 |
| 3958 | LGALS3 | 1.372492396 | 0.005743293 | 0.035498084 |
| 3982 | LIM2 | 2.618595055 | 0.022624076 | 0.103389284 |
| 3984 | LIMK1 | 1.331057748 | 4.21E-04 | 0.004189861 |
| 3992 | FADS1 | 1.611539525 | 2.44E-05 | 3.66E-04 |
| 3993 | LLGL2 | 1.588163241 | 1.42E-13 | 1.21E-11 |
| 3995 | FADS3 | 1.53273148 | 0.002422947 | 0.017874252 |
| 400668 | PRSS57 | 4.14284463 | 0.01124591 | 0.05990385 |
| 401115 | C4orf48 | 1.088434784 | 0.016311253 | 0.079978473 |
| 401124 | DTHD1 | 2.811155618 | 6.06E-17 | 8.62E-15 |
| 401190 | RGS7BP | 4.657638711 | 0.010198798 | 0.055711509 |
| 4018 | LPA | 3.202348785 | 0.025383782 | 0.112743789 |
| 4052 | LTBP1 | 2.905671582 | 0.026273277 | 0.115666667 |
| 4053 | LTBP2 | 1.089072219 | 0.004056493 | 0.0268997 |
| 4058 | LTK | 4.174936381 | 3.73E-18 | 6.87E-16 |
| 4067 | LYN | 1.4719855 | 2.66E-08 | 8.54E-07 |
| 4068 | SH2D1A | 1.071650018 | 1.20E-06 | 2.56E-05 |
| 4088 | SMAD3 | 1.688109478 | 1.79E-09 | 7.19E-08 |
| 4092 | SMAD7 | 2.045688436 | 6.16E-08 | 1.84E-06 |
| 4094 | MAF | 2.30139866 | 2.90E-16 | 3.80E-14 |
| 4121 | MAN1A1 | 1.952342709 | 4.16E-09 | 1.59E-07 |
| 4137 | MAPT | 2.167007042 | 0.003329998 | 0.022917264 |
| 414301 | DDI1 | 3.885688381 | 2.68E-04 | 0.002857633 |
| 4145 | MATK | 2.341926336 | 8.48E-08 | 2.45E-06 |
| 4147 | MATN2 | 2.353445175 | 0.018215845 | 0.087575778 |
| 415116 | PIM3 | 1.110619011 | 6.63E-05 | 8.71E-04 |
| 4199 | ME1 | 3.775746936 | 1.47E-14 | 1.51E-12 |
| 4211 | MEIS1 | 2.254402779 | 0.037731558 | 0.151382447 |
| 4217 | MAP3K5 | 1.05199949 | 3.14E-05 | 4.55E-04 |
| 4241 | MELTF | 3.088884347 | 0.018598732 | 0.088936518 |
| 4261 | CIITA | 1.316527296 | 4.27E-08 | 1.31E-06 |
| 4267 | CD99 | 1.302807621 | 4.62E-10 | 2.09E-08 |
| 4301 | AFDN | 2.177973711 | 2.00E-05 | 3.09E-04 |
| 431705 | ASTL | 1.918026166 | 9.41E-05 | 0.00117503 |
| 4332 | MNDA | 1.841531891 | 0.021532472 | 0.099235845 |
| 435 | ASL | 1.066886706 | 1.09E-08 | 3.76E-07 |
| 4357 | MPST | 1.823324572 | 5.15E-06 | 9.25E-05 |
| 439921 | MXRA7 | 1.47628617 | 1.70E-07 | 4.50E-06 |
| 4439 | MSH5 | 1.136901446 | 1.37E-05 | 2.21E-04 |
| 445 | ASS1 | 1.180806889 | 0.003024306 | 0.021245469 |
| 445347 | TARP | 1.996054678 | 5.06E-18 | 9.11E-16 |
| 445815 | PALM2AKAP2 | 1.735087337 | 2.25E-08 | 7.34E-07 |
| 4493 | MT1E | 1.250346835 | 3.50E-04 | 0.003597901 |
| 4542 | MYO1F | 1.978744174 | 3.99E-13 | 3.14E-11 |
| 4597 | MVD | 1.158393593 | 6.66E-09 | 2.39E-07 |
| 4603 | MYBL1 | 2.390916229 | 9.10E-13 | 6.76E-11 |
| 4642 | MYO1D | 1.307187999 | 0.010971557 | 0.058962101 |
| 4644 | MYO5A | 1.09482575 | 1.79E-09 | 7.19E-08 |
| 4646 | MYO6 | 1.574328399 | 0.025097116 | 0.111885799 |
| 4674 | NAP1L2 | 1.003297244 | 0.038616271 | 0.15380613 |
| 4684 | NCAM1 | 3.2388431 | 5.88E-10 | 2.59E-08 |
| 4752 | NEK3 | 1.373412351 | 0.003579818 | 0.024329826 |
| 4756 | NEO1 | 1.683272837 | 7.23E-08 | 2.13E-06 |
| 4758 | NEU1 | 1.050461978 | 9.24E-04 | 0.007996493 |
| 478 | ATP1A3 | 1.06646652 | 0.001163817 | 0.009760922 |
| 4783 | NFIL3 | 1.727305947 | 9.19E-06 | 1.54E-04 |
| 4792 | NFKBIA | 1.059572158 | 2.28E-04 | 0.002507821 |
| 4793 | NFKBIB | 1.16867264 | 9.36E-08 | 2.68E-06 |
| 4794 | NFKBIE | 1.372186706 | 1.10E-04 | 0.001343028 |
| 4804 | NGFR | 2.095185651 | 0.033289899 | 0.137978194 |
| 4815 | NINJ2 | 1.745264811 | 1.41E-06 | 2.93E-05 |
| 4818 | NKG7 | 2.321504464 | 3.65E-05 | 5.17E-04 |
| 4828 | NMB | 1.278770005 | 0.030265108 | 0.128493834 |
| 4837 | NNMT | 4.519273566 | 0.016733084 | 0.081700515 |
| 4864 | NPC1 | 1.330130303 | 1.58E-09 | 6.49E-08 |
| 4920 | ROR2 | 2.532783243 | 0.002159264 | 0.016334923 |
| 4921 | DDR2 | 1.840758307 | 0.002265245 | 0.01694728 |
| 493 | ATP2B4 | 1.630417166 | 6.56E-08 | 1.94E-06 |
| 4938 | OAS1 | 1.300826303 | 8.74E-04 | 0.007625327 |
| 5045 | FURIN | 1.359424482 | 7.69E-06 | 1.31E-04 |
| 50509 | COL5A3 | 3.644498774 | 0.00763922 | 0.044440881 |
| 50515 | CHST11 | 1.536039968 | 6.21E-11 | 3.26E-09 |
| 50651 | SLC45A1 | 1.097573254 | 0.033812945 | 0.139462948 |
| 5066 | PAM | 1.440895206 | 2.43E-06 | 4.74E-05 |
| 5097 | PCDH1 | 3.89523041 | 0.007962679 | 0.045943546 |
| 51043 | ZBTB7B | 1.271188278 | 0.004658805 | 0.029977553 |
| 51063 | CALHM2 | 1.505685127 | 8.66E-04 | 0.007581508 |
| 51148 | CERCAM | 1.555729034 | 5.51E-05 | 7.41E-04 |
| 5118 | PCOLCE | 1.032372647 | 0.01765573 | 0.085441211 |
| 51225 | ABI3 | 1.519749541 | 1.45E-05 | 2.33E-04 |
| 51228 | GLTP | 1.054837095 | 4.01E-10 | 1.84E-08 |
| 51232 | CRIM1 | 1.542197342 | 1.77E-04 | 0.002019782 |
| 51237 | MZB1 | 1.410272958 | 0.007201289 | 0.042300388 |
| 51278 | IER5 | 1.626452219 | 7.69E-07 | 1.73E-05 |
| 5128 | CDK17 | 1.088468555 | 1.55E-05 | 2.48E-04 |
| 51314 | NME8 | 2.943007651 | 2.34E-08 | 7.62E-07 |
| 5133 | PDCD1 | 1.815420999 | 2.05E-07 | 5.33E-06 |
| 51330 | TNFRSF12A | 1.997311811 | 0.009329274 | 0.051951072 |
| 51332 | SPTBN5 | 1.566991648 | 4.05E-07 | 9.95E-06 |
| 51348 | KLRF1 | 3.243002708 | 7.09E-12 | 4.33E-10 |
| 5141 | PDE4A | 1.556970393 | 3.80E-04 | 0.003852957 |
| 5144 | PDE4D | 1.127620042 | 1.07E-04 | 0.001312503 |
| 51523 | CXXC5 | 1.794263077 | 7.18E-05 | 9.31E-04 |
| 5155 | PDGFB | 1.40367492 | 0.007719875 | 0.044802277 |
| 5159 | PDGFRB | 1.539869763 | 0.002143149 | 0.016242054 |
| 51655 | RASD1 | 1.583876547 | 0.011002588 | 0.059016416 |
| 51676 | ASB2 | 1.76369405 | 0.035473678 | 0.144412089 |
| 51726 | DNAJB11 | 1.137919811 | 5.19E-06 | 9.30E-05 |
| 51744 | CD244 | 1.961007979 | 3.14E-10 | 1.47E-08 |
| 51760 | SYT17 | 3.610287613 | 0.042645948 | 0.164997113 |
| 51776 | MAP3K20 | 1.630995366 | 0.007036744 | 0.041463376 |
| 5295 | PIK3R1 | 1.390064502 | 2.46E-04 | 0.002661664 |
| 53346 | TM6SF1 | 6.330003836 | 1.18E-07 | 3.31E-06 |
| 5336 | PLCG2 | 2.167684831 | 1.54E-17 | 2.55E-15 |
| 5337 | PLD1 | 4.109327213 | 6.15E-12 | 3.81E-10 |
| 53405 | CLIC5 | 1.052386457 | 0.008843731 | 0.04987086 |
| 5341 | PLEK | 2.314337026 | 5.61E-08 | 1.69E-06 |
| 5351 | PLOD1 | 1.650974192 | 7.44E-08 | 2.19E-06 |
| 53637 | S1PR5 | 2.681441572 | 3.27E-11 | 1.79E-09 |
| 5364 | PLXNB1 | 2.622606876 | 5.31E-05 | 7.18E-04 |
| 5366 | PMAIP1 | 1.578492318 | 2.43E-04 | 0.00263406 |
| 54103 | GSAP | 1.088411127 | 8.50E-13 | 6.36E-11 |
| 54206 | ERRFI1 | 1.296161257 | 9.90E-05 | 0.001229816 |
| 54331 | GNG2 | 1.153424315 | 4.55E-05 | 6.28E-04 |
| 54434 | SSH1 | 1.131625422 | 4.20E-07 | 1.03E-05 |
| 54438 | GFOD1 | 1.63016499 | 1.45E-07 | 3.98E-06 |
| 54457 | TAF7L | 3.409767883 | 0.047562385 | 0.178509258 |
| 54477 | PLEKHA5 | 1.391762491 | 1.11E-04 | 0.001360537 |
| 54620 | FBXL19 | 1.590065501 | 0.034269797 | 0.140832387 |
| 54733 | SLC35F2 | 1.024278853 | 0.001814819 | 0.014212021 |
| 54762 | GRAMD1C | 3.922545601 | 0.001385524 | 0.011283553 |
| 54796 | BNC2 | 3.280772384 | 1.90E-10 | 9.26E-09 |
| 54836 | BSPRY | 4.008692167 | 0.010990403 | 0.058996647 |
| 54843 | SYTL2 | 1.82381291 | 1.02E-18 | 1.98E-16 |
| 54875 | CNTLN | 2.166409149 | 0.002328756 | 0.017315266 |
| 54897 | CASZ1 | 1.806657774 | 3.99E-14 | 3.77E-12 |
| 54997 | TESC | 2.469967785 | 7.07E-05 | 9.20E-04 |
| 55001 | TTC22 | 1.448722953 | 6.54E-04 | 0.006007615 |
| 55020 | TTC38 | 2.194716998 | 5.12E-10 | 2.30E-08 |
| 55026 | TMEM255A | 2.294155697 | 4.02E-04 | 0.004026122 |
| 55038 | CDCA4 | 1.139616301 | 1.51E-07 | 4.09E-06 |
| 55062 | WIPI1 | 1.608291475 | 4.85E-09 | 1.80E-07 |
| 55106 | SLFN12 | 1.031283467 | 4.06E-05 | 5.67E-04 |
| 55120 | FANCL | 1.01372042 | 0.003088883 | 0.021600482 |
| 55143 | CDCA8 | 1.123984263 | 0.008613461 | 0.048935224 |
| 55172 | DNAAF2 | 1.205009979 | 7.22E-04 | 0.006506175 |
| 55205 | ZNF532 | 2.293143694 | 7.97E-14 | 7.21E-12 |
| 5521 | PPP2R2B | 2.158605921 | 2.29E-13 | 1.92E-11 |
| 55224 | ETNK2 | 1.449109254 | 0.001541456 | 0.012338448 |
| 55313 | CPPED1 | 1.059962155 | 3.44E-05 | 4.91E-04 |
| 553158 | PRR5-ARHGAP8 | 1.218905766 | 0.005986725 | 0.036628107 |
| 55356 | SLC22A15 | 1.585908976 | 0.015763471 | 0.078115731 |
| 55366 | LGR4 | 1.979917665 | 0.045683396 | 0.173263137 |
| 55450 | CAMK2N1 | 2.03051942 | 1.28E-11 | 7.50E-10 |
| 55501 | CHST12 | 1.341076517 | 9.18E-11 | 4.68E-09 |
| 55502 | HES6 | 3.031946677 | 0.004277673 | 0.028080317 |
| 5551 | PRF1 | 2.112234461 | 1.78E-10 | 8.70E-09 |
| 55512 | SMPD3 | 1.059393901 | 0.005372221 | 0.033587784 |
| 55521 | TRIM36 | 2.538802674 | 0.025132497 | 0.112014023 |
| 55605 | KIF21A | 1.433630499 | 2.48E-11 | 1.39E-09 |
| 55646 | LYAR | 1.242553247 | 6.31E-09 | 2.28E-07 |
| 55784 | MCTP2 | 1.680516698 | 7.75E-11 | 3.99E-09 |
| 5580 | PRKCD | 1.40989766 | 2.56E-05 | 3.82E-04 |
| 55884 | WSB2 | 1.091550824 | 4.33E-06 | 7.96E-05 |
| 55911 | APOBR | 1.235139108 | 2.80E-05 | 4.11E-04 |
| 55969 | RAB5IF | 1.807025735 | 2.47E-10 | 1.18E-08 |
| 55973 | BCAP29 | 1.040599418 | 3.75E-07 | 9.30E-06 |
| 5598 | MAPK7 | 1.243758358 | 3.29E-06 | 6.21E-05 |
| 55997 | CFC1 | 4.973670635 | 0.010802124 | 0.058273617 |
| 5606 | MAP2K3 | 1.06586658 | 8.31E-08 | 2.42E-06 |
| 56062 | KLHL4 | 6.034002565 | 1.05E-05 | 1.75E-04 |
| 56100 | PCDHGB6 | 2.561296685 | 2.46E-04 | 0.002661412 |
| 56101 | PCDHGB5 | 1.628384959 | 0.041497105 | 0.161735492 |
| 56103 | PCDHGB2 | 2.57281921 | 0.001075883 | 0.009121826 |
| 56106 | PCDHGA10 | 1.298275983 | 0.017299472 | 0.084108525 |
| 56243 | KIAA1217 | 5.459157926 | 4.15E-05 | 5.78E-04 |
| 56548 | CHST7 | 2.001280705 | 1.50E-04 | 0.001767041 |
| 56895 | AGPAT4 | 1.21976042 | 2.98E-06 | 5.67E-05 |
| 57060 | PCBP4 | 1.226710475 | 6.14E-06 | 1.07E-04 |
| 57085 | AGTRAP | 1.013529921 | 0.005848124 | 0.035983112 |
| 57094 | CPA6 | 4.000772441 | 0.04407621 | 0.168794231 |
| 57136 | APMAP | 1.750200009 | 1.89E-17 | 3.05E-15 |
| 57168 | ASPHD2 | 1.463613379 | 0.021082991 | 0.097537143 |
| 57282 | SLC4A10 | 4.34340061 | 3.36E-29 | 2.10E-26 |
| 5729 | PTGDR | 2.279232018 | 0.011462201 | 0.060731003 |
| 5732 | PTGER2 | 1.089112441 | 0.008280879 | 0.047311719 |
| 5743 | PTGS2 | 4.674389188 | 0.039307454 | 0.15553146 |
| 57458 | TMCC3 | 2.358117141 | 3.29E-10 | 1.52E-08 |
| 5746 | PTH2R | 4.330225973 | 0.020934232 | 0.096941156 |
| 57476 | GRAMD1B | 1.340601725 | 6.15E-06 | 1.07E-04 |
| 57480 | PLEKHG1 | 2.136844531 | 5.34E-04 | 0.005072912 |
| 57493 | HEG1 | 1.296673536 | 4.35E-06 | 8.00E-05 |
| 57497 | LRFN2 | 3.894134999 | 2.48E-07 | 6.32E-06 |
| 57514 | ARHGAP31 | 1.259312418 | 0.00132031 | 0.010846407 |
| 57568 | SIPA1L2 | 3.552875931 | 6.91E-12 | 4.24E-10 |
| 57572 | DOCK6 | 4.460707986 | 0.001089298 | 0.009226326 |
| 57582 | KCNT1 | 2.316798038 | 0.036207714 | 0.146729681 |
| 57595 | PDZD4 | 2.179576785 | 2.85E-04 | 0.003023141 |
| 576 | ADGRB2 | 1.617482992 | 0.024670912 | 0.110422069 |
| 5763 | PTMS | 2.740073179 | 2.08E-05 | 3.19E-04 |
| 57646 | USP28 | 1.73973856 | 6.79E-07 | 1.56E-05 |
| 5778 | PTPN7 | 1.120355503 | 4.46E-07 | 1.08E-05 |
| 57787 | MARK4 | 1.384069372 | 3.95E-04 | 0.003980677 |
| 5782 | PTPN12 | 1.705455877 | 1.19E-09 | 5.02E-08 |
| 57823 | SLAMF7 | 2.467189236 | 5.85E-11 | 3.08E-09 |
| 5783 | PTPN13 | 2.564585938 | 2.83E-06 | 5.43E-05 |
| 5791 | PTPRE | 1.300024055 | 1.16E-06 | 2.50E-05 |
| 5795 | PTPRJ | 1.508738268 | 1.30E-06 | 2.73E-05 |
| 5797 | PTPRM | 2.552428192 | 5.78E-10 | 2.57E-08 |
| 5805 | PTS | 1.105033667 | 7.32E-04 | 0.006585951 |
| 58480 | RHOU | 1.584296531 | 0.024816302 | 0.110926118 |
| 58489 | ABHD17C | 4.193321168 | 2.49E-07 | 6.33E-06 |
| 5858 | PZP | 2.72282567 | 5.28E-20 | 1.21E-17 |
| 5873 | RAB27A | 1.462595609 | 3.28E-16 | 4.26E-14 |
| 5874 | RAB27B | 2.197643485 | 3.64E-07 | 9.04E-06 |
| 58985 | IL22RA1 | 3.637829579 | 0.008738707 | 0.04949302 |
| 5899 | RALB | 1.05720298 | 1.39E-04 | 0.001649785 |
| 59 | ACTA2 | 1.749225687 | 8.15E-04 | 0.007200597 |
| 5900 | RALGDS | 1.249850462 | 2.78E-08 | 8.84E-07 |
| 59084 | ENPP5 | 1.340311611 | 7.51E-04 | 0.006728467 |
| 5911 | RAP2A | 1.672164619 | 2.72E-08 | 8.68E-07 |
| 5916 | RARG | 1.07179025 | 1.12E-05 | 1.85E-04 |
| 59269 | HIVEP3 | 1.536186138 | 1.95E-05 | 3.04E-04 |
| 59277 | NTN4 | 4.006054055 | 3.98E-07 | 9.83E-06 |
| 5932 | RBBP8 | 1.177203624 | 0.037579072 | 0.151055656 |
| 59339 | PLEKHA2 | 1.005415459 | 7.47E-06 | 1.28E-04 |
| 59352 | LGR6 | 2.62577902 | 2.41E-10 | 1.15E-08 |
| 5962 | RDX | 1.023921702 | 4.54E-07 | 1.10E-05 |
| 5971 | RELB | 1.177708373 | 0.010511528 | 0.056978405 |
| 598 | BCL2L1 | 1.170709225 | 2.65E-04 | 0.002829595 |
| 5998 | RGS3 | 1.385595268 | 3.55E-04 | 0.003646576 |
| 6004 | RGS16 | 1.889880401 | 0.04027754 | 0.158478682 |
| 602 | BCL3 | 1.089719081 | 5.45E-04 | 0.005162823 |
| 60489 | APOBEC3G | 1.79314022 | 5.39E-07 | 1.28E-05 |
| 60675 | PROK2 | 2.231315927 | 9.17E-06 | 1.54E-04 |
| 6096 | RORB | 1.830265117 | 0.003118226 | 0.02176072 |
| 6097 | RORC | 3.442535205 | 6.53E-10 | 2.84E-08 |
| 6238 | RRBP1 | 1.050547445 | 7.42E-07 | 1.68E-05 |
| 6256 | RXRA | 1.236760078 | 6.86E-04 | 0.006241061 |
| 6262 | RYR2 | 2.771633968 | 0.019066377 | 0.090557405 |
| 6275 | S100A4 | 1.645312642 | 2.23E-12 | 1.52E-10 |
| 6277 | S100A6 | 1.028333706 | 2.35E-06 | 4.58E-05 |
| 6282 | S100A11 | 1.167004376 | 0.002905282 | 0.020622341 |
| 631 | BFSP1 | 2.130764149 | 4.23E-04 | 0.004198905 |
| 6310 | ATXN1 | 1.341860997 | 7.96E-12 | 4.81E-10 |
| 6348 | CCL3 | 2.466933052 | 6.50E-07 | 1.50E-05 |
| 6349 | CCL3L1 | 3.589329267 | 0.002949935 | 0.020844153 |
| 6351 | CCL4 | 2.387016165 | 1.47E-09 | 6.13E-08 |
| 6352 | CCL5 | 2.061974124 | 5.35E-18 | 9.52E-16 |
| 6364 | CCL20 | 4.895327856 | 3.43E-08 | 1.08E-06 |
| 6375 | XCL1 | 2.18619203 | 1.76E-05 | 2.77E-04 |
| 6385 | SDC4 | 2.100285655 | 0.00255926 | 0.018684365 |
| 639 | PRDM1 | 2.063017858 | 7.02E-16 | 8.49E-14 |
| 640 | BLK | 2.321396814 | 0.010419874 | 0.056645172 |
| 64005 | MYO1G | 1.000808492 | 1.34E-05 | 2.18E-04 |
| 64065 | PERP | 1.957658712 | 1.12E-14 | 1.17E-12 |
| 64127 | NOD2 | 1.200532045 | 0.00854293 | 0.048628323 |
| 64215 | DNAJC1 | 1.420538993 | 1.66E-07 | 4.42E-06 |
| 64218 | SEMA4A | 2.2242978 | 4.67E-10 | 2.10E-08 |
| 64283 | ARHGEF28 | 2.612459954 | 6.81E-05 | 8.93E-04 |
| 64388 | GREM2 | 2.203753809 | 0.047426706 | 0.17811854 |
| 64407 | RGS18 | 1.347290295 | 0.006532357 | 0.039201093 |
| 644100 | ARL14EPL | 4.987005664 | 0.010718182 | 0.05791694 |
| 64411 | ARAP3 | 2.387523413 | 1.88E-08 | 6.23E-07 |
| 64420 | SUSD1 | 1.908124733 | 3.02E-12 | 2.01E-10 |
| 6444 | SGCD | 2.92929711 | 1.11E-06 | 2.40E-05 |
| 644815 | FAM83G | 1.545792711 | 2.42E-04 | 0.002626931 |
| 6452 | SH3BP2 | 1.27946947 | 1.89E-05 | 2.96E-04 |
| 64764 | CREB3L2 | 1.184757484 | 2.40E-08 | 7.79E-07 |
| 6484 | ST3GAL4 | 2.179773058 | 3.99E-07 | 9.83E-06 |
| 649 | BMP1 | 1.449674697 | 0.024639349 | 0.110309976 |
| 6494 | SIPA1 | 1.083293239 | 7.32E-07 | 1.66E-05 |
| 6504 | SLAMF1 | 1.402745428 | 9.98E-07 | 2.19E-05 |
| 6509 | SLC1A4 | 1.866549264 | 4.61E-05 | 6.35E-04 |
| 6510 | SLC1A5 | 1.734049721 | 2.14E-07 | 5.54E-06 |
| 6512 | SLC1A7 | 2.091106802 | 4.64E-04 | 0.004548121 |
| 651746 | ANKRD33B | 1.29975163 | 0.015757679 | 0.078109902 |
| 6569 | SLC34A1 | 4.213073506 | 0.023646171 | 0.106681993 |
| 6675 | UAP1 | 1.340194622 | 1.89E-05 | 2.96E-04 |
| 6693 | SPN | 1.242480625 | 7.45E-04 | 0.006677061 |
| 6697 | SPR | 2.741250559 | 0.028954687 | 0.124428689 |
| 6714 | SRC | 2.325583367 | 0.037605819 | 0.151055656 |
| 6764 | DENND2B | 2.191125858 | 0.048456442 | 0.181301871 |
| 6785 | ELOVL4 | 1.510464478 | 6.01E-04 | 0.005609843 |
| 6836 | SURF4 | 1.162006903 | 4.46E-09 | 1.68E-07 |
| 6846 | XCL2 | 2.680072717 | 0.001293702 | 0.010657809 |
| 6884 | TAF13 | 1.165840357 | 0.001508751 | 0.012112235 |
| 6916 | TBXAS1 | 1.054431031 | 6.64E-04 | 0.006078471 |
| 7036 | TFR2 | 1.238014859 | 5.31E-05 | 7.18E-04 |
| 7039 | TGFA | 2.796589613 | 1.46E-04 | 0.001722458 |
| 7040 | TGFB1 | 1.563204147 | 2.69E-12 | 1.81E-10 |
| 7045 | TGFBI | 1.774913205 | 0.033416132 | 0.138264353 |
| 7047 | TGM4 | 2.792345441 | 0.010529506 | 0.057021068 |
| 7049 | TGFBR3 | 2.224205822 | 3.36E-15 | 3.79E-13 |
| 7057 | THBS1 | 1.909114196 | 2.30E-11 | 1.30E-09 |
| 7071 | KLF10 | 1.206733987 | 1.48E-04 | 0.001745347 |
| 7088 | TLE1 | 2.669502566 | 1.42E-05 | 2.29E-04 |
| 7098 | TLR3 | 1.315389964 | 8.08E-05 | 0.00103075 |
| 7107 | GPR137B | 1.160649499 | 2.61E-06 | 5.05E-05 |
| 7124 | TNF | 2.86526966 | 1.28E-10 | 6.37E-09 |
| 7133 | TNFRSF1B | 1.827302574 | 6.38E-10 | 2.78E-08 |
| 7162 | TPBG | 4.706534989 | 0.008125966 | 0.046631306 |
| 719 | C3AR1 | 2.104089474 | 4.41E-06 | 8.09E-05 |
| 7204 | TRIO | 1.536379123 | 3.46E-07 | 8.61E-06 |
| 728047 | GOLGA8O | 1.841639924 | 0.049887976 | 0.185143469 |
| 729230 | CCR2 | 2.586744727 | 2.57E-05 | 3.83E-04 |
| 7295 | TXN | 1.029408235 | 4.21E-07 | 1.03E-05 |
| 7305 | TYROBP | 2.972561907 | 7.28E-18 | 1.27E-15 |
| 7371 | UCK2 | 1.458549655 | 9.60E-07 | 2.12E-05 |
| 7412 | VCAM1 | 3.158517743 | 0.012098496 | 0.063348653 |
| 7414 | VCL | 1.488573059 | 1.62E-05 | 2.57E-04 |
| 7434 | VIPR2 | 1.951040264 | 1.45E-06 | 2.99E-05 |
| 745 | MYRF | 1.730494471 | 9.35E-04 | 0.008072429 |
| 7461 | CLIP2 | 1.576315191 | 7.34E-04 | 0.006599879 |
| 7462 | LAT2 | 1.203008724 | 5.54E-05 | 7.44E-04 |
| 7465 | WEE1 | 1.480381373 | 8.54E-07 | 1.90E-05 |
| 7471 | WNT1 | 1.451201367 | 5.24E-04 | 0.005001815 |
| 7481 | WNT11 | 4.494941958 | 4.69E-07 | 1.13E-05 |
| 7504 | XK | 3.491585913 | 0.013588354 | 0.069279177 |
| 7533 | YWHAH | 1.284486432 | 1.32E-07 | 3.65E-06 |
| 7584 | ZNF35 | 1.553475525 | 0.001303091 | 0.010720565 |
| 760 | CA2 | 2.430123276 | 2.13E-06 | 4.20E-05 |
| 7634 | ZNF80 | 1.02238776 | 0.028337097 | 0.122465232 |
| 7704 | ZBTB16 | 2.342230712 | 9.94E-05 | 0.001234695 |
| 7746 | ZSCAN9 | 1.596595674 | 5.06E-05 | 6.90E-04 |
| 782 | CACNB1 | 1.210101744 | 2.24E-06 | 4.39E-05 |
| 784 | CACNB3 | 1.54298215 | 0.004376851 | 0.028554146 |
| 7855 | FZD5 | 1.439835355 | 0.043472524 | 0.167075241 |
| 788 | SLC25A20 | 1.039476278 | 0.002374555 | 0.017578564 |
| 78995 | HROB | 1.213299418 | 0.015554755 | 0.077307816 |
| 79026 | AHNAK | 1.41324128 | 2.52E-13 | 2.08E-11 |
| 7905 | REEP5 | 1.09115714 | 2.67E-07 | 6.77E-06 |
| 79071 | ELOVL6 | 1.482844168 | 2.56E-13 | 2.10E-11 |
| 79156 | PLEKHF1 | 1.751460977 | 8.22E-07 | 1.84E-05 |
| 79174 | CRELD2 | 1.052414437 | 0.003387386 | 0.023227203 |
| 79180 | EFHD2 | 2.089689507 | 7.65E-11 | 3.95E-09 |
| 79187 | FSD1 | 1.440328639 | 0.023014542 | 0.104500965 |
| 79414 | LRFN3 | 1.126180561 | 0.040801627 | 0.159871713 |
| 7942 | TFEB | 1.473675962 | 2.30E-04 | 0.002529296 |
| 79586 | CHPF | 1.085110175 | 0.011725511 | 0.061893579 |
| 79642 | ARSJ | 3.896683897 | 0.004145456 | 0.027414441 |
| 79651 | RHBDF2 | 1.451893251 | 3.97E-12 | 2.56E-10 |
| 79682 | CENPU | 1.002803132 | 0.03282945 | 0.136504369 |
| 79683 | ZDHHC14 | 1.998413049 | 1.73E-06 | 3.52E-05 |
| 79734 | KCTD17 | 1.228645725 | 0.015114142 | 0.075606451 |
| 79815 | NIPAL2 | 1.542553655 | 1.08E-05 | 1.79E-04 |
| 7982 | ST7 | 1.191951502 | 2.91E-06 | 5.55E-05 |
| 79850 | TLCD3A | 1.11128821 | 0.028936728 | 0.124383095 |
| 79888 | LPCAT1 | 2.153183106 | 1.33E-08 | 4.54E-07 |
| 79895 | ATP8B4 | 3.777896479 | 0.002265092 | 0.01694728 |
| 79899 | PRR5L | 1.793044011 | 1.59E-05 | 2.54E-04 |
| 79931 | TNIP3 | 1.861334807 | 4.47E-12 | 2.84E-10 |
| 80005 | DOCK5 | 1.305039167 | 0.023386258 | 0.105791405 |
| 80021 | TMEM62 | 1.059759843 | 5.12E-04 | 0.00490292 |
| 80119 | PIF1 | 1.846648158 | 1.91E-04 | 0.002159724 |
| 80149 | ZC3H12A | 1.190245852 | 1.05E-04 | 0.001292581 |
| 80218 | NAA50 | 1.261872304 | 1.05E-04 | 0.001290375 |
| 80256 | FAM214B | 1.063271981 | 0.002614883 | 0.019016619 |
| 80301 | PLEKHO2 | 1.543042944 | 3.31E-04 | 0.003444923 |
| 80310 | PDGFD | 2.555555522 | 3.32E-04 | 0.003446514 |
| 8038 | ADAM12 | 2.986229021 | 2.93E-07 | 7.39E-06 |
| 80380 | PDCD1LG2 | 2.365790162 | 0.005410606 | 0.033762424 |
| 80759 | KHDC1 | 1.849182634 | 4.05E-08 | 1.25E-06 |
| 80790 | CMIP | 1.026309688 | 1.03E-04 | 0.001266218 |
| 80854 | SETD7 | 1.070824615 | 0.026734833 | 0.117119746 |
| 81 | ACTN4 | 2.017830986 | 3.82E-09 | 1.47E-07 |
| 81030 | ZBP1 | 1.045693239 | 1.17E-08 | 4.02E-07 |
| 8111 | GPR68 | 1.358925453 | 0.00250601 | 0.018390811 |
| 8140 | SLC7A5 | 1.805022489 | 5.18E-13 | 3.95E-11 |
| 81532 | MOB2 | 1.160146412 | 2.20E-04 | 0.002441876 |
| 81544 | GDPD5 | 1.082043938 | 4.78E-06 | 8.68E-05 |
| 81553 | CYRIA | 2.300433731 | 5.21E-07 | 1.24E-05 |
| 81563 | C1orf21 | 2.607263466 | 5.85E-10 | 2.59E-08 |
| 81567 | TXNDC5 | 1.510231648 | 2.35E-04 | 0.002569423 |
| 81839 | VANGL1 | 1.690440395 | 2.03E-04 | 0.002275227 |
| 81873 | ARPC5L | 1.596032924 | 1.36E-13 | 1.17E-11 |
| 81926 | ABHD17A | 1.03546772 | 1.35E-04 | 0.001605826 |
| 824 | CAPN2 | 1.453605477 | 2.38E-07 | 6.09E-06 |
| 8292 | COLQ | 2.357891201 | 2.38E-08 | 7.74E-07 |
| 8320 | EOMES | 2.58064388 | 1.40E-18 | 2.70E-16 |
| 8322 | FZD4 | 2.261346312 | 2.79E-06 | 5.36E-05 |
| 834 | CASP1 | 1.149170332 | 2.61E-08 | 8.38E-07 |
| 83856 | FSD1L | 2.34812489 | 6.30E-04 | 0.005828464 |
| 83872 | HMCN1 | 4.019589062 | 0.042802028 | 0.165374138 |
| 83888 | FGFBP2 | 2.144049981 | 2.03E-08 | 6.70E-07 |
| 83930 | STARD3NL | 1.081907756 | 4.85E-04 | 0.004700952 |
| 83937 | RASSF4 | 1.824004659 | 4.55E-06 | 8.30E-05 |
| 84056 | KATNAL1 | 1.08878764 | 0.001600301 | 0.012726451 |
| 84069 | PLEKHN1 | 1.630636971 | 0.049096793 | 0.182929331 |
| 84131 | CEP78 | 1.36201568 | 1.99E-10 | 9.62E-09 |
| 84282 | RNF135 | 1.152427645 | 8.57E-06 | 1.45E-04 |
| 84443 | FRMPD3 | 2.287760518 | 2.46E-04 | 0.002661664 |
| 84448 | ABLIM2 | 1.141302905 | 0.007829011 | 0.045326842 |
| 84501 | SPIRE2 | 1.330142458 | 0.045597951 | 0.17305542 |
| 84525 | HOPX | 2.148504106 | 4.58E-04 | 0.004496275 |
| 84542 | SANBR | 1.081803546 | 0.0108834 | 0.058581352 |
| 84557 | MAP1LC3A | 1.209414929 | 9.76E-04 | 0.008387876 |
| 84628 | NTNG2 | 1.353927121 | 0.010067489 | 0.05522597 |
| 84687 | PPP1R9B | 1.09461935 | 0.01044047 | 0.056720409 |
| 84695 | LOXL3 | 1.810354034 | 0.015395076 | 0.076716984 |
| 8477 | GPR65 | 1.234466788 | 3.28E-12 | 2.17E-10 |
| 84790 | TUBA1C | 1.057339299 | 1.42E-05 | 2.28E-04 |
| 8482 | SEMA7A | 2.311460519 | 0.004492008 | 0.029148105 |
| 84876 | ORAI1 | 1.241311266 | 1.24E-07 | 3.46E-06 |
| 84940 | CORO6 | 1.071316874 | 0.008187316 | 0.046900694 |
| 84951 | TNS4 | 2.871858043 | 0.00847388 | 0.048267747 |
| 84957 | RELT | 1.183560521 | 4.17E-04 | 0.004163155 |
| 84971 | ATG4D | 1.053273158 | 9.51E-04 | 0.008197358 |
| 84975 | MFSD5 | 1.330799059 | 2.18E-04 | 0.002414752 |
| 8503 | PIK3R3 | 2.357342714 | 6.57E-05 | 8.64E-04 |
| 8507 | ENC1 | 2.291193856 | 4.18E-18 | 7.61E-16 |
| 8510 | MMP23B | 1.329629348 | 0.007221042 | 0.042372296 |
| 8522 | GAS7 | 2.391473948 | 2.98E-11 | 1.64E-09 |
| 8530 | CST7 | 2.472096501 | 2.16E-18 | 4.06E-16 |
| 85379 | KIAA1671 | 1.824497468 | 0.025906371 | 0.114522942 |
| 8542 | APOL1 | 1.020334925 | 8.76E-05 | 0.001107379 |
| 85461 | TANC1 | 2.600233951 | 0.006568688 | 0.039377224 |
| 8553 | BHLHE40 | 2.352089073 | 8.49E-05 | 0.001075685 |
| 8612 | PLPP2 | 2.463042779 | 7.01E-04 | 0.006359739 |
| 8638 | OASL | 1.915853962 | 4.13E-09 | 1.58E-07 |
| 864 | RUNX3 | 1.398269271 | 1.43E-13 | 1.21E-11 |
| 8644 | AKR1C3 | 1.699870565 | 7.84E-04 | 0.006981221 |
| 8671 | SLC4A4 | 2.72188936 | 5.28E-09 | 1.95E-07 |
| 8672 | EIF4G3 | 1.081727239 | 1.28E-04 | 0.001543966 |
| 8676 | STX11 | 2.56045306 | 9.05E-12 | 5.41E-10 |
| 8682 | PEA15 | 1.280882934 | 7.96E-06 | 1.35E-04 |
| 8692 | HYAL2 | 1.490108021 | 0.030507994 | 0.129312267 |
| 8707 | B3GALT2 | 1.045496991 | 4.54E-04 | 0.004468877 |
| 8740 | TNFSF14 | 1.957738962 | 2.57E-10 | 1.22E-08 |
| 8744 | TNFSF9 | 2.254307439 | 2.06E-04 | 0.002301364 |
| 8751 | ADAM15 | 1.073493015 | 9.66E-05 | 0.001202752 |
| 8784 | TNFRSF18 | 1.702315442 | 0.029933579 | 0.12766279 |
| 8787 | RGS9 | 2.199940761 | 4.45E-06 | 8.14E-05 |
| 8807 | IL18RAP | 3.226024321 | 2.05E-17 | 3.27E-15 |
| 8809 | IL18R1 | 2.288504005 | 5.35E-27 | 2.66E-24 |
| 8819 | SAP30 | 1.629126812 | 0.008359673 | 0.047681409 |
| 8826 | IQGAP1 | 1.173171819 | 2.72E-11 | 1.50E-09 |
| 8870 | IER3 | 1.153447056 | 0.002927585 | 0.020729507 |
| 8877 | SPHK1 | 2.054022881 | 0.010858193 | 0.058501496 |
| 89 | ACTN3 | 5.453149902 | 6.55E-05 | 8.62E-04 |
| 8934 | RAB29 | 1.02629808 | 1.12E-09 | 4.75E-08 |
| 8935 | SKAP2 | 1.816999635 | 4.39E-13 | 3.41E-11 |
| 8972 | MGAM | 6.650749246 | 3.29E-05 | 4.73E-04 |
| 89839 | ARHGAP11B | 1.247050335 | 0.002747106 | 0.019715554 |
| 89853 | MVB12B | 1.166227038 | 1.85E-07 | 4.84E-06 |
| 8986 | RPS6KA4 | 1.395869571 | 6.43E-04 | 0.005932526 |
| 8989 | TRPA1 | 1.986946802 | 0.011999378 | 0.062888037 |
| 89944 | GLB1L2 | 1.640714464 | 3.05E-04 | 0.003203722 |
| 90102 | PHLDB2 | 1.997486564 | 4.10E-17 | 6.03E-15 |
| 9022 | CLIC3 | 1.429359644 | 1.13E-05 | 1.86E-04 |
| 90313 | TP53I13 | 1.2212504 | 0.004751033 | 0.030491011 |
| 9034 | CCRL2 | 1.085203382 | 8.46E-05 | 0.001072756 |
| 90427 | BMF | 2.120948973 | 1.78E-04 | 0.002030972 |
| 9046 | DOK2 | 1.669358675 | 2.49E-10 | 1.18E-08 |
| 9047 | SH2D2A | 1.513924923 | 2.10E-08 | 6.89E-07 |
| 90557 | CCDC74A | 2.376471434 | 0.019014888 | 0.090362641 |
| 9079 | LDB2 | 3.555983039 | 0.001576548 | 0.012573007 |
| 9088 | PKMYT1 | 2.505671065 | 0.008614196 | 0.048935224 |
| 9124 | PDLIM1 | 1.895975269 | 8.20E-16 | 9.84E-14 |
| 9134 | CCNE2 | 2.187194351 | 0.004791198 | 0.030701041 |
| 9143 | SYNGR3 | 3.046456943 | 5.53E-06 | 9.81E-05 |
| 91461 | PKDCC | 4.762408178 | 0.020169046 | 0.094204175 |
| 9149 | DYRK1B | 1.397634106 | 2.10E-14 | 2.05E-12 |
| 92211 | CDHR1 | 3.00726641 | 2.20E-12 | 1.51E-10 |
| 92293 | TMEM132C | 4.788404172 | 0.015593615 | 0.07745546 |
| 92304 | SCGB3A1 | 4.134576495 | 0.027033077 | 0.118120519 |
| 9231 | DLG5 | 2.992954453 | 2.45E-23 | 7.83E-21 |
| 9232 | PTTG1 | 1.136433379 | 2.05E-06 | 4.07E-05 |
| 9242 | MSC | 2.164754812 | 6.13E-07 | 1.42E-05 |
| 92521 | SPECC1 | 1.212674842 | 3.00E-05 | 4.36E-04 |
| 9254 | CACNA2D2 | 1.948826527 | 4.70E-09 | 1.76E-07 |
| 9256 | TSPOAP1 | 2.025450982 | 4.20E-23 | 1.32E-20 |
| 9261 | MAPKAPK2 | 1.255321943 | 1.05E-04 | 0.001290375 |
| 9265 | CYTH3 | 1.71942363 | 1.47E-07 | 4.02E-06 |
| 9289 | ADGRG1 | 2.102526133 | 0.001980327 | 0.015212473 |
| 92906 | HNRNPLL | 1.629299147 | 3.36E-11 | 1.83E-09 |
| 9294 | S1PR2 | 1.36218192 | 1.26E-06 | 2.69E-05 |
| 93010 | B3GNT7 | 1.909720958 | 6.45E-09 | 2.32E-07 |
| 93099 | DMKN | 1.790839415 | 6.00E-04 | 0.005605129 |
| 933 | CD22 | 1.000224154 | 0.039195019 | 0.155157263 |
| 9331 | B4GALT6 | 3.50181556 | 5.54E-05 | 7.44E-04 |
| 9334 | B4GALT5 | 2.02219573 | 4.23E-12 | 2.71E-10 |
| 93589 | CACNA2D4 | 1.282751975 | 5.90E-05 | 7.85E-04 |
| 93663 | ARHGAP18 | 1.830388333 | 2.14E-06 | 4.23E-05 |
| 9368 | SLC9A3R1 | 1.394289479 | 5.28E-04 | 0.005035065 |
| 9381 | OTOF | 1.364353997 | 0.025457758 | 0.112983807 |
| 94015 | TTYH2 | 1.053438782 | 7.10E-04 | 0.006421649 |
| 94059 | LENG9 | 1.20791804 | 0.038675583 | 0.15389397 |
| 94120 | SYTL3 | 1.229993167 | 2.85E-06 | 5.46E-05 |
| 9415 | FADS2 | 2.108360548 | 0.001593359 | 0.012683168 |
| 94241 | TP53INP1 | 1.138498001 | 2.16E-07 | 5.57E-06 |
| 9437 | NCR1 | 3.040020795 | 7.25E-14 | 6.60E-12 |
| 9447 | AIM2 | 1.228200969 | 0.032720173 | 0.136150355 |
| 945 | CD33 | 1.709695897 | 0.014704291 | 0.073949694 |
| 9467 | SH3BP5 | 1.60754996 | 7.67E-05 | 9.88E-04 |
| 9510 | ADAMTS1 | 2.609802243 | 8.26E-04 | 0.007288482 |
| 9537 | TP53I11 | 2.433748147 | 4.31E-09 | 1.63E-07 |
| 9560 | CCL4L2 | 2.779668503 | 6.08E-16 | 7.40E-14 |
| 9580 | SOX13 | 2.407078351 | 0.002323861 | 0.017308511 |
| 959 | CD40LG | 1.297574041 | 0.013004052 | 0.067003845 |
| 9592 | IER2 | 1.635663951 | 1.78E-08 | 5.95E-07 |
| 9628 | RGS6 | 3.966803544 | 0.034728676 | 0.142428781 |
| 9638 | FEZ1 | 2.803876024 | 1.25E-21 | 3.52E-19 |
| 9645 | MICAL2 | 1.821982505 | 5.18E-11 | 2.73E-09 |
| 965 | CD58 | 1.768826916 | 3.86E-05 | 5.42E-04 |
| 967 | CD63 | 1.562540732 | 2.82E-10 | 1.33E-08 |
| 9679 | FAM53B | 1.455593927 | 8.75E-06 | 1.47E-04 |
| 9693 | RAPGEF2 | 1.0276558 | 2.39E-04 | 0.002605428 |
| 971 | CD72 | 1.242287151 | 0.004711265 | 0.030269072 |
| 9715 | FAM131B | 2.744826617 | 0.003666605 | 0.024780337 |
| 972 | CD74 | 1.330268401 | 1.21E-16 | 1.66E-14 |
| 9734 | HDAC9 | 2.013454727 | 3.21E-10 | 1.49E-08 |
| 9749 | PHACTR2 | 2.131346066 | 1.24E-16 | 1.69E-14 |
| 975 | CD81 | 1.763154743 | 5.67E-09 | 2.08E-07 |
| 9755 | TBKBP1 | 1.99183467 | 4.57E-07 | 1.11E-05 |
| 9760 | TOX | 1.879850498 | 3.78E-17 | 5.62E-15 |
| 9780 | PIEZO1 | 1.286108525 | 3.08E-07 | 7.69E-06 |
| 9788 | MTSS1 | 1.026366149 | 1.74E-09 | 7.04E-08 |
| 9811 | CTIF | 2.542148013 | 0.025911975 | 0.114522942 |
| 9817 | KEAP1 | 1.321830902 | 5.71E-06 | 1.01E-04 |
| 9824 | ARHGAP11A | 1.025466268 | 5.72E-05 | 7.65E-04 |
| 9832 | JAKMIP2 | 1.980813873 | 4.02E-15 | 4.42E-13 |
| 9839 | ZEB2 | 2.343102247 | 1.04E-10 | 5.24E-09 |
| 9858 | PPP1R26 | 1.917919494 | 9.84E-04 | 0.008452499 |
| 9880 | ZBTB39 | 1.23105548 | 5.00E-06 | 9.00E-05 |
| 9891 | NUAK1 | 3.058868553 | 9.64E-05 | 0.001200756 |
| 9934 | P2RY14 | 3.882949164 | 3.97E-04 | 0.003995949 |
| 9945 | GFPT2 | 2.331491355 | 0.00335595 | 0.02305836 |
| 9982 | FGFBP1 | 5.995018604 | 9.01E-07 | 2.00E-05 |
| 9989 | PPP4R1 | 1.193508281 | 1.16E-11 | 6.83E-10 |
