# Supplementary material for: KLRG1-expressing CD8+ T cells are exhausted and polyfunctional in patients with chronic hepatitis B
Source: PLoS One. 2024 May 22;19(5):e0303945. doi: 10.1371/journal.pone.0303945 (PMC11111010; doi:10.1371/journal.pone.0303945)
Supplement: S3 Table — (DOCX) [file pone.0303945.s008.docx]

**KLRG1-expressing CD8+ T cells are exhausted and polyfunctional in patients with chronic hepatitis B.**

S3 Table. Genes downregulated in KLRG1+ vs. KLRG1- CD8 T cells from CHB patients.

| Gene Symbol | Gene ID | log2FoldChange | Pvalue | Qvalue |
| --- | --- | --- | --- | --- |
| A1BG | 1 | -1.547876084 | 3.36E-09 | 1.30E-07 |
| MUC8 | 100129528 | -1.886723342 | 0.039390684 | 0.155822475 |
| C17orf107 | 100130311 | -1.218317481 | 0.022432697 | 0.102660355 |
| LOC100131107 | 100131107 | -2.158308136 | 7.50E-05 | 9.67E-04 |
| ARMCX4 | 100131755 | -2.41615681 | 1.53E-11 | 8.82E-10 |
| ZNF717 | 100131827 | -1.87173634 | 2.26E-06 | 4.42E-05 |
| SRRM5 | 100170229 | -1.564135919 | 0.014487698 | 0.073055817 |
| CDH4 | 1002 | -2.709449187 | 0.001479439 | 0.011930286 |
| RASA4B | 100271927 | -1.348036993 | 1.19E-07 | 3.34E-06 |
| TTC34 | 100287898 | -1.176580165 | 0.030015149 | 0.127899944 |
| TMEM238L | 100289255 | -2.815786604 | 0.023209309 | 0.105187772 |
| LOC100505502 | 100505502 | -2.425028059 | 1.13E-07 | 3.19E-06 |
| SPATA1 | 100505741 | -1.036811219 | 0.001269049 | 0.010500293 |
| KRBOX1 | 100506243 | -4.272972194 | 0.022653046 | 0.103431074 |
| FPGT-TNNI3K | 100526835 | -1.144778314 | 0.01314531 | 0.06747288 |
| ISY1-RAB43 | 100534599 | -1.383292111 | 2.42E-13 | 2.01E-11 |
| ABCB6 | 10058 | -1.581775413 | 1.06E-06 | 2.31E-05 |
| CEP295NL | 100653515 | -2.19760218 | 0.048629294 | 0.181828005 |
| FSBP | 100861412 | -2.024814401 | 2.69E-08 | 8.59E-07 |
| ZNF891 | 101060200 | -1.857303759 | 3.83E-15 | 4.27E-13 |
| RASA4 | 10156 | -1.403376151 | 1.51E-07 | 4.08E-06 |
| ZNF256 | 10172 | -1.036851794 | 0.012476544 | 0.064986318 |
| CFAP97D2 | 101929355 | -2.736193525 | 4.67E-08 | 1.43E-06 |
| CALCRL | 10203 | -5.50921798 | 3.44E-05 | 4.91E-04 |
| MPZL2 | 10205 | -2.146258604 | 3.26E-04 | 0.003392526 |
| PATJ | 10207 | -1.240506915 | 6.49E-07 | 1.50E-05 |
| FSTL3 | 10272 | -1.174069537 | 8.93E-04 | 0.007766254 |
| LOC102723713 | 102723713 | -4.063555981 | 0.035133042 | 0.143447211 |
| LOC102723996 | 102723996 | -1.512966313 | 0.034832682 | 0.142695103 |
| NET1 | 10276 | -1.853413668 | 8.67E-06 | 1.46E-04 |
| SPEG | 10290 | -3.106832383 | 2.04E-07 | 5.30E-06 |
| LAMC3 | 10319 | -3.713144296 | 3.41E-11 | 1.85E-09 |
| SIRPB1 | 10326 | -1.011715317 | 1.03E-04 | 0.001266583 |
| SEMA3A | 10371 | -4.091610972 | 3.46E-06 | 6.50E-05 |
| LRRN2 | 10446 | -1.700571619 | 0.005824328 | 0.035878206 |
| TACC3 | 10460 | -1.455098547 | 2.86E-16 | 3.78E-14 |
| FST | 10468 | -4.068746469 | 0.040683799 | 0.15959479 |
| ZBTB18 | 10472 | -1.703148135 | 2.47E-06 | 4.80E-05 |
| FBLN5 | 10516 | -1.742664038 | 1.78E-07 | 4.71E-06 |
| CEBPE | 1053 | -5.375655131 | 4.54E-05 | 6.27E-04 |
| LOC105371932 | 105371932 | -1.484468021 | 1.74E-05 | 2.74E-04 |
| AKAP3 | 10566 | -1.393710263 | 0.014828487 | 0.074442668 |
| TRAPPC2B | 10597 | -1.053106498 | 0.007473468 | 0.04359652 |
| PAICS | 10606 | -1.337274308 | 3.95E-13 | 3.12E-11 |
| SPINT2 | 10653 | -2.935424412 | 9.49E-11 | 4.82E-09 |
| DLL3 | 10683 | -1.456024358 | 0.036878856 | 0.148985647 |
| RAI2 | 10742 | -4.846077438 | 6.23E-09 | 2.26E-07 |
| GRAP | 10750 | -2.052999586 | 4.38E-06 | 8.04E-05 |
| PLK2 | 10769 | -1.431652216 | 0.040714422 | 0.159677907 |
| LOC107985876 | 107985876 | -1.542618205 | 5.07E-05 | 6.91E-04 |
| LOC107986860 | 107986860 | -3.543074254 | 9.53E-05 | 0.001188782 |
| CCR9 | 10803 | -1.524617336 | 0.003819344 | 0.025587787 |
| GJB6 | 10804 | -3.623470308 | 4.77E-04 | 0.004639015 |
| PPP1R13L | 10848 | -1.326790547 | 0.016789165 | 0.081927057 |
| PROKR1 | 10887 | -4.360964668 | 0.022234858 | 0.101835046 |
| EDAR | 10913 | -6.526413423 | 8.74E-08 | 2.51E-06 |
| MSL3 | 10943 | -1.025553276 | 2.38E-09 | 9.39E-08 |
| IL24 | 11009 | -1.120713879 | 0.002563293 | 0.018705739 |
| ZNF660-ZNF197 | 110354863 | -5.573604529 | 0.037030432 | 0.149419647 |
| PIM2 | 11040 | -1.239992907 | 5.66E-09 | 2.08E-07 |
| RAPGEF4 | 11069 | -4.813789125 | 7.10E-04 | 0.006421649 |
| HSF2BP | 11077 | -2.315248333 | 1.73E-04 | 0.001980674 |
| ADCY5 | 111 | -1.776623653 | 0.020203575 | 0.094292638 |
| PRDM5 | 11107 | -2.102360256 | 0.001854739 | 0.01445431 |
| RCAN3 | 11123 | -2.742860928 | 5.89E-16 | 7.22E-14 |
| ZWINT | 11130 | -1.317056233 | 0.001040822 | 0.008860079 |
| PKIG | 11142 | -1.929678803 | 2.60E-08 | 8.38E-07 |
| ADAMTS7 | 11173 | -2.593263965 | 3.19E-05 | 4.60E-04 |
| ADAMTS6 | 11174 | -1.006639927 | 0.015425736 | 0.076843446 |
| CHKA | 1119 | -1.934913541 | 4.29E-09 | 1.63E-07 |
| CHEK2 | 11200 | -1.179255826 | 2.50E-04 | 0.002692598 |
| LOC112268384 | 112268384 | -1.715916268 | 3.75E-04 | 0.003811741 |
| FILIP1L | 11259 | -1.7334823 | 1.05E-06 | 2.27E-05 |
| WDR89 | 112840 | -1.041985172 | 8.38E-04 | 0.007372457 |
| CHRM5 | 1133 | -2.339512201 | 0.003197063 | 0.022173731 |
| PIK3IP1 | 113791 | -1.449430706 | 1.08E-12 | 7.81E-11 |
| FAM83F | 113828 | -1.61696462 | 0.003992366 | 0.026557711 |
| TRIM9 | 114088 | -1.63682292 | 0.013420603 | 0.068636104 |
| TXNRD3 | 114112 | -5.982792788 | 4.86E-07 | 1.16E-05 |
| CYGB | 114757 | -2.633678022 | 3.47E-05 | 4.95E-04 |
| FMNL2 | 114793 | -1.883265576 | 0.034585888 | 0.141924582 |
| RNF157 | 114804 | -1.591333662 | 2.02E-06 | 4.02E-05 |
| ZBED9 | 114821 | -5.462073001 | 0.002013329 | 0.01542398 |
| RHPN1 | 114822 | -1.748047486 | 5.74E-04 | 0.0053861 |
| OSBPL1A | 114876 | -1.544824429 | 4.98E-04 | 0.004798113 |
| TMEM123 | 114908 | -1.251543017 | 5.60E-17 | 8.04E-15 |
| GPRASP2 | 114928 | -7.253484603 | 3.11E-09 | 1.21E-07 |
| MARCHF3 | 115123 | -1.5455755 | 2.98E-04 | 0.003137511 |
| KCTD12 | 115207 | -2.855394591 | 5.25E-05 | 7.13E-04 |
| GPR146 | 115330 | -1.255736783 | 9.38E-04 | 0.008086326 |
| RMI2 | 116028 | -3.58959718 | 3.69E-04 | 0.00377371 |
| TOP1MT | 116447 | -1.114385648 | 6.87E-06 | 1.19E-04 |
| THEM4 | 117145 | -1.156453705 | 5.70E-09 | 2.09E-07 |
| SLC16A10 | 117247 | -5.986248892 | 2.62E-97 | 2.22E-93 |
| RLN3 | 117579 | -2.198842415 | 0.027787038 | 0.120698161 |
| PARD3B | 117583 | -3.534773011 | 8.06E-05 | 0.001029673 |
| CFAP70 | 118491 | -1.254075414 | 2.19E-06 | 4.30E-05 |
| ADD2 | 119 | -2.943137139 | 1.37E-13 | 1.17E-11 |
| TMEM45B | 120224 | -1.476162769 | 5.64E-04 | 0.005311243 |
| CLN5 | 1203 | -1.366143436 | 2.25E-08 | 7.34E-07 |
| H4-16 | 121504 | -1.121974157 | 0.037435523 | 0.150659062 |
| BTBD11 | 121551 | -1.348769386 | 2.97E-04 | 0.003133031 |
| SPIC | 121599 | -4.654383263 | 4.66E-04 | 0.004560088 |
| CCR7 | 1236 | -4.227944779 | 2.85E-29 | 1.86E-26 |
| HYKK | 123688 | -1.06044024 | 0.019137868 | 0.090792158 |
| HSF5 | 124535 | -1.73621498 | 5.33E-06 | 9.50E-05 |
| SPATA32 | 124783 | -3.121258372 | 0.001622602 | 0.012879594 |
| TMEM132E | 124842 | -2.120498126 | 0.026927026 | 0.117748334 |
| EFCAB13 | 124989 | -1.469409303 | 0.002575413 | 0.018769906 |
| ACER1 | 125981 | -2.092226176 | 1.59E-07 | 4.26E-06 |
| TMIGD2 | 126259 | -1.236754354 | 0.001636514 | 0.012983935 |
| GIPC3 | 126326 | -2.828079078 | 6.61E-04 | 0.00606517 |
| CNN3 | 1266 | -2.919442812 | 1.46E-19 | 3.30E-17 |
| KDF1 | 126695 | -2.284548046 | 3.69E-13 | 2.94E-11 |
| KLHDC9 | 126823 | -2.267738472 | 0.013266656 | 0.067992614 |
| GOLT1A | 127845 | -4.943040267 | 0.013608727 | 0.069325852 |
| FITM2 | 128486 | -1.090848131 | 1.78E-06 | 3.62E-05 |
| TSHZ2 | 128553 | -4.278488102 | 2.12E-17 | 3.36E-15 |
| GAB4 | 128954 | -5.414806295 | 8.70E-04 | 0.007602563 |
| COL5A2 | 1290 | -2.972762853 | 7.98E-07 | 1.79E-05 |
| COL6A1 | 1291 | -1.163370593 | 0.004378894 | 0.028556466 |
| TRABD2A | 129293 | -3.476190226 | 2.68E-30 | 2.05E-27 |
| COL6A3 | 1293 | -3.519750588 | 1.77E-14 | 1.78E-12 |
| ICA1L | 130026 | -1.197745689 | 2.67E-11 | 1.49E-09 |
| COL11A2 | 1302 | -1.605852482 | 0.004164764 | 0.027531367 |
| PLEKHH2 | 130271 | -2.626449168 | 8.23E-08 | 2.40E-06 |
| ACVR1C | 130399 | -1.287985438 | 1.21E-06 | 2.59E-05 |
| MDH1B | 130752 | -3.091352591 | 0.009139396 | 0.05116308 |
| MTERF4 | 130916 | -1.037328097 | 1.59E-09 | 6.54E-08 |
| DCBLD2 | 131566 | -4.160144868 | 2.10E-06 | 4.15E-05 |
| PPARGC1B | 133522 | -1.200493184 | 4.29E-06 | 7.90E-05 |
| PACRG | 135138 | -2.198318946 | 0.002556012 | 0.018684365 |
| ADORA2B | 136 | -2.074889145 | 0.028856499 | 0.124101277 |
| CLDN7 | 1366 | -1.375296495 | 0.0034278 | 0.023456798 |
| CR2 | 1380 | -3.644330476 | 1.52E-20 | 3.69E-18 |
| ASB9 | 140462 | -3.627976861 | 6.54E-04 | 0.006007615 |
| C20orf96 | 140680 | -1.280396253 | 0.002508724 | 0.018402749 |
| ZBTB46 | 140685 | -1.104129822 | 0.023933146 | 0.107603783 |
| TLDC2 | 140711 | -1.553293224 | 0.001784731 | 0.014015316 |
| NEURL2 | 140825 | -3.043415006 | 0.003252793 | 0.022495718 |
| ISM1 | 140862 | -1.992640923 | 0.034964317 | 0.143026624 |
| ASB15 | 142685 | -1.058116759 | 0.007276858 | 0.042655442 |
| SYT9 | 143425 | -4.761601004 | 0.025684129 | 0.113813175 |
| UBQLNL | 143630 | -1.512988834 | 0.044689445 | 0.170447618 |
| SESN3 | 143686 | -1.592612706 | 1.67E-07 | 4.45E-06 |
| LAYN | 143903 | -2.164683907 | 0.00209415 | 0.015949831 |
| PTGR2 | 145482 | -1.464295929 | 3.02E-06 | 5.73E-05 |
| HAPLN3 | 145864 | -2.021315299 | 3.86E-12 | 2.50E-10 |
| FBXL16 | 146330 | -1.484278613 | 0.006752429 | 0.04023639 |
| ZNF534 | 147658 | -1.876193078 | 0.007178663 | 0.042196773 |
| C19orf18 | 147685 | -2.779909406 | 0.003657519 | 0.024738685 |
| ZNF418 | 147686 | -1.770115334 | 0.006490985 | 0.039022004 |
| ZNF563 | 147837 | -2.362388957 | 1.16E-15 | 1.39E-13 |
| CTH | 1491 | -1.074636551 | 0.025183253 | 0.112151631 |
| CNIH3 | 149111 | -1.406128495 | 0.035195735 | 0.143626097 |
| CLDN19 | 149461 | -1.79113448 | 0.01955303 | 0.091966626 |
| C22orf15 | 150248 | -1.663878482 | 0.004405454 | 0.028672607 |
| CHADL | 150356 | -2.518519331 | 0.018990646 | 0.090325662 |
| FAM117B | 150864 | -2.355807351 | 3.22E-24 | 1.18E-21 |
| SEPTIN10 | 151011 | -3.064549324 | 0.002744889 | 0.019707997 |
| KANSL1L | 151050 | -1.584337546 | 1.44E-06 | 2.98E-05 |
| C2CD6 | 151254 | -3.421234037 | 8.50E-04 | 0.007446561 |
| GPR155 | 151556 | -1.663890053 | 1.09E-14 | 1.15E-12 |
| BTLA | 151888 | -1.408286841 | 0.001269491 | 0.010500293 |
| IGSF11 | 152404 | -3.533006339 | 0.005121567 | 0.032340402 |
| GIMAP8 | 155038 | -1.473003422 | 6.64E-07 | 1.53E-05 |
| CRYGN | 155051 | -3.436867625 | 0.007755717 | 0.044979436 |
| SLC2A7 | 155184 | -4.869170186 | 4.96E-04 | 0.004790284 |
| METTL27 | 155368 | -2.852059276 | 0.004195161 | 0.027689041 |
| CYP2B6 | 1555 | -3.251517909 | 0.015629253 | 0.077599117 |
| PRAG1 | 157285 | -1.657361952 | 1.91E-14 | 1.90E-12 |
| CYP2J2 | 1573 | -5.416744731 | 2.62E-10 | 1.24E-08 |
| CLVS1 | 157807 | -2.286969279 | 0.007010163 | 0.041335539 |
| SAXO1 | 158297 | -2.01446184 | 0.047603951 | 0.178625647 |
| SHOC1 | 158401 | -1.39466841 | 0.010671084 | 0.057695451 |
| KIAA1958 | 158405 | -1.173723504 | 7.77E-06 | 1.32E-04 |
| FAAH2 | 158584 | -1.281053753 | 0.004829499 | 0.030876316 |
| ZDHHC15 | 158866 | -1.388795219 | 0.002620251 | 0.019031545 |
| USP51 | 158880 | -1.729178254 | 0.002659687 | 0.019234995 |
| C11orf65 | 160140 | -1.416875236 | 5.49E-04 | 0.005191148 |
| CD55 | 1604 | -1.577371828 | 2.48E-10 | 1.18E-08 |
| DGKA | 1606 | -1.628123256 | 2.01E-20 | 4.79E-18 |
| TMEM30B | 161291 | -1.592251746 | 4.61E-05 | 6.35E-04 |
| STRC | 161497 | -1.444588592 | 2.38E-04 | 0.002600119 |
| ADAD2 | 161931 | -4.269763285 | 0.020610538 | 0.095953818 |
| DBH | 1621 | -5.074140902 | 0.002678012 | 0.019350984 |
| ZNF610 | 162963 | -2.135840589 | 4.73E-06 | 8.60E-05 |
| ZNF497 | 162968 | -1.7629072 | 6.53E-13 | 4.93E-11 |
| ZNF550 | 162972 | -1.253592761 | 1.05E-08 | 3.64E-07 |
| ZNF781 | 163115 | -1.286164485 | 0.003135918 | 0.021866145 |
| DCT | 1638 | -2.278310918 | 2.29E-04 | 0.002520626 |
| TTC24 | 164118 | -1.560726025 | 2.18E-21 | 5.58E-19 |
| AKR1C1 | 1645 | -5.209755768 | 3.02E-04 | 0.003178952 |
| AEBP1 | 165 | -3.548777042 | 1.57E-12 | 1.12E-10 |
| ADGRA3 | 166647 | -1.550158872 | 6.22E-04 | 0.005772544 |
| GSDME | 1687 | -2.022273103 | 0.03547243 | 0.144412089 |
| GLIS3 | 169792 | -3.401245943 | 2.82E-09 | 1.10E-07 |
| SPIN3 | 169981 | -2.41280111 | 1.05E-07 | 2.98E-06 |
| ZCCHC12 | 170261 | -4.688813204 | 0.021103694 | 0.097578639 |
| ADAMTS17 | 170691 | -1.253553535 | 0.003072473 | 0.021503502 |
| ZNF721 | 170960 | -1.01428782 | 2.76E-05 | 4.06E-04 |
| ANKRD24 | 170961 | -3.203523923 | 2.44E-05 | 3.66E-04 |
| NLRP6 | 171389 | -2.156981699 | 6.78E-11 | 3.53E-09 |
| DLG2 | 1740 | -1.968804121 | 1.89E-06 | 3.79E-05 |
| SARDH | 1757 | -2.019159027 | 0.009256317 | 0.051631588 |
| DNAH6 | 1768 | -3.735365953 | 6.26E-14 | 5.82E-12 |
| DNMT3A | 1788 | -1.618370731 | 2.23E-15 | 2.61E-13 |
| DOCK1 | 1793 | -4.338238142 | 0.019475764 | 0.091841505 |
| DSC1 | 1823 | -5.510316002 | 6.75E-29 | 3.94E-26 |
| EDA | 1896 | -1.95931038 | 2.22E-05 | 3.39E-04 |
| EDN1 | 1906 | -2.019543536 | 0.002845168 | 0.02028171 |
| EFNA1 | 1942 | -1.330863376 | 0.001873999 | 0.01456761 |
| MEGF6 | 1953 | -1.42861766 | 9.81E-04 | 0.008427356 |
| MYRFL | 196446 | -2.659749984 | 0.004969054 | 0.031613271 |
| METTL21C | 196541 | -1.971367684 | 0.004038389 | 0.026807759 |
| AIF1 | 199 | -3.165721714 | 1.09E-31 | 9.20E-29 |
| CELA1 | 1990 | -3.251466451 | 0.001832331 | 0.014309428 |
| KRTCAP3 | 200634 | -1.364151722 | 0.017807169 | 0.085854904 |
| FBXO15 | 201456 | -1.674248058 | 0.033033809 | 0.137152332 |
| ADGRE1 | 2015 | -1.589331376 | 0.028850068 | 0.124101277 |
| DNAH12 | 201625 | -3.102846897 | 0.045947839 | 0.173915292 |
| FAM153B | 202134 | -4.310187056 | 5.40E-12 | 3.41E-10 |
| SUSD3 | 203328 | -2.401515067 | 6.50E-15 | 7.06E-13 |
| EPHA1 | 2041 | -4.465950082 | 2.26E-09 | 8.98E-08 |
| EPHB4 | 2050 | -3.429662414 | 1.17E-13 | 1.02E-11 |
| EPHX1 | 2052 | -1.959324253 | 4.10E-04 | 0.004105188 |
| EPHX2 | 2053 | -3.0970046 | 4.89E-27 | 2.51E-24 |
| EPS8 | 2059 | -2.157285363 | 0.00744336 | 0.043450838 |
| ERBB3 | 2065 | -2.248380737 | 4.22E-04 | 0.004198905 |
| EVPL | 2125 | -3.721157412 | 8.13E-05 | 0.001036656 |
| F2RL1 | 2150 | -3.089096572 | 5.92E-09 | 2.15E-07 |
| F5 | 2153 | -1.454512104 | 9.30E-04 | 0.008037803 |
| FBLN2 | 2199 | -4.528681064 | 1.76E-19 | 3.91E-17 |
| FBP1 | 2203 | -1.483920118 | 1.91E-06 | 3.83E-05 |
| OAF | 220323 | -1.946437956 | 1.33E-04 | 0.001586417 |
| FCER1G | 2207 | -2.148997894 | 1.65E-08 | 5.54E-07 |
| FAM171A1 | 221061 | -1.156212023 | 6.08E-06 | 1.06E-04 |
| ARMC12 | 221481 | -1.880995887 | 0.013340146 | 0.068286538 |
| GPC2 | 221914 | -3.81260267 | 6.51E-04 | 0.005989433 |
| ZSCAN23 | 222696 | -2.592862159 | 4.77E-05 | 6.53E-04 |
| RSPH10B | 222967 | -3.537029069 | 0.034896168 | 0.142782362 |
| ABCD2 | 225 | -1.176717269 | 4.63E-07 | 1.12E-05 |
| FGF9 | 2254 | -1.182967529 | 5.54E-06 | 9.81E-05 |
| FGFR4 | 2264 | -2.701953568 | 0.00130536 | 0.010734014 |
| FHIT | 2272 | -3.094491539 | 8.31E-04 | 0.007330978 |
| ZFP30 | 22835 | -1.385074014 | 0.002009029 | 0.015404983 |
| CNKSR2 | 22866 | -3.189805359 | 4.04E-20 | 9.49E-18 |
| MLXIP | 22877 | -1.473398625 | 1.43E-17 | 2.40E-15 |
| FKBP5 | 2289 | -1.073547853 | 1.34E-08 | 4.54E-07 |
| BTBD3 | 22903 | -6.273357982 | 1.29E-05 | 2.09E-04 |
| ZP1 | 22917 | -2.362542242 | 0.006778332 | 0.04033783 |
| RAB3GAP1 | 22930 | -1.863629463 | 4.03E-26 | 1.84E-23 |
| DIP2C | 22982 | -1.180495047 | 1.11E-08 | 3.83E-07 |
| SORCS3 | 22986 | -7.120367624 | 1.31E-07 | 3.63E-06 |
| KDM4B | 23030 | -1.347686585 | 3.36E-04 | 0.003483335 |
| MYT1L | 23040 | -5.226600671 | 0.032149667 | 0.134537293 |
| CLUAP1 | 23059 | -1.21681375 | 4.37E-09 | 1.65E-07 |
| ZNF609 | 23060 | -1.134216528 | 0.00117569 | 0.009839866 |
| SPART | 23111 | -1.455229717 | 2.40E-05 | 3.61E-04 |
| FLNB | 2317 | -1.776672394 | 2.30E-12 | 1.56E-10 |
| CEP68 | 23177 | -1.283696168 | 3.71E-17 | 5.56E-15 |
| PASK | 23178 | -1.961854359 | 1.59E-25 | 6.91E-23 |
| RGL1 | 23179 | -1.933188311 | 9.37E-04 | 0.008082793 |
| KANK1 | 23189 | -1.268778327 | 0.007674286 | 0.044598882 |
| FLT1 | 2321 | -2.606975646 | 4.12E-05 | 5.75E-04 |
| TMEM131L | 23240 | -1.016103615 | 1.52E-09 | 6.31E-08 |
| DENND5A | 23258 | -2.609255142 | 1.28E-20 | 3.18E-18 |
| MCF2L | 23263 | -2.155523546 | 8.71E-04 | 0.00760608 |
| CAMSAP2 | 23271 | -3.171220568 | 2.02E-06 | 4.02E-05 |
| TRIM2 | 23321 | -4.690316279 | 1.06E-05 | 1.75E-04 |
| NEDD4L | 23327 | -1.79940892 | 6.69E-04 | 0.006119089 |
| TTC28 | 23331 | -2.371538105 | 2.59E-15 | 2.98E-13 |
| AFF2 | 2334 | -2.844522747 | 0.010039863 | 0.055128036 |
| ZNF629 | 23361 | -1.551318289 | 4.91E-04 | 0.004746419 |
| KIAA0895 | 23366 | -2.038094928 | 0.001150169 | 0.009674113 |
| CRTC1 | 23373 | -1.127999157 | 1.18E-06 | 2.52E-05 |
| KCNH3 | 23416 | -1.817979588 | 3.86E-05 | 5.42E-04 |
| CRB1 | 23418 | -3.244367882 | 0.00561958 | 0.034847983 |
| GRIP1 | 23426 | -1.844639319 | 0.00551115 | 0.034301285 |
| SLC7A8 | 23428 | -2.908412668 | 1.94E-08 | 6.42E-07 |
| ABCA6 | 23460 | -1.643988176 | 0.011202428 | 0.059786481 |
| GCAT | 23464 | -1.588717918 | 4.11E-04 | 0.004111237 |
| LEPROTL1 | 23484 | -1.06615374 | 2.98E-07 | 7.47E-06 |
| LRRC8B | 23507 | -1.121243609 | 2.63E-05 | 3.90E-04 |
| TTC9 | 23508 | -1.11283203 | 0.014524722 | 0.073178737 |
| SEC14L2 | 23541 | -1.123779986 | 3.95E-04 | 0.003978537 |
| PADI4 | 23569 | -3.657855595 | 8.03E-25 | 3.16E-22 |
| VSIG2 | 23584 | -2.753790556 | 0.001939674 | 0.014968129 |
| SSBP2 | 23635 | -1.131512916 | 1.99E-05 | 3.08E-04 |
| LDOC1 | 23641 | -1.909802526 | 0.001297451 | 0.010679364 |
| LY96 | 23643 | -1.797763009 | 4.35E-04 | 0.004305423 |
| PLXNB2 | 23654 | -3.020376487 | 0.004911119 | 0.031303527 |
| FUT8 | 2530 | -1.07602389 | 9.72E-09 | 3.41E-07 |
| LCLAT1 | 253558 | -1.140639882 | 3.98E-04 | 0.004003833 |
| CERS6 | 253782 | -2.827425336 | 9.15E-19 | 1.80E-16 |
| METAP1D | 254042 | -1.48484891 | 7.51E-11 | 3.89E-09 |
| SNX32 | 254122 | -1.693650227 | 0.001822573 | 0.014252959 |
| PHYHD1 | 254295 | -1.248525035 | 4.59E-05 | 6.33E-04 |
| NAALADL2 | 254827 | -2.671056117 | 0.030052376 | 0.127911558 |
| CFAP299 | 255119 | -4.946277779 | 0.014874594 | 0.074628744 |
| ANKK1 | 255239 | -1.296782714 | 0.031501939 | 0.132432345 |
| EIF2S3B | 255308 | -1.274141298 | 0.003627181 | 0.024602317 |
| TCP11L2 | 255394 | -1.101569724 | 4.41E-05 | 6.11E-04 |
| RNF144B | 255488 | -1.812177679 | 1.24E-09 | 5.23E-08 |
| ANKRD31 | 256006 | -2.705216466 | 7.46E-12 | 4.53E-10 |
| ST6GALNAC3 | 256435 | -2.592707921 | 1.37E-07 | 3.77E-06 |
| SERINC5 | 256987 | -2.890846667 | 3.58E-24 | 1.26E-21 |
| C9orf43 | 257169 | -1.413317464 | 0.044373332 | 0.169628104 |
| KLHL34 | 257240 | -3.879090575 | 0.002792246 | 0.019980202 |
| RAD54B | 25788 | -1.176248845 | 0.002640001 | 0.019125313 |
| GCA | 25801 | -1.307714846 | 9.26E-06 | 1.55E-04 |
| TECPR1 | 25851 | -1.146478476 | 9.93E-15 | 1.06E-12 |
| PLEKHG4 | 25894 | -1.796709922 | 1.37E-12 | 9.84E-11 |
| ALS2CL | 259173 | -2.242399884 | 2.11E-07 | 5.47E-06 |
| TMIE | 259236 | -2.54439144 | 1.14E-06 | 2.46E-05 |
| MYRIP | 25924 | -4.233210568 | 0.029368623 | 0.125760429 |
| TAS2R20 | 259295 | -1.318699363 | 0.00635018 | 0.038393747 |
| C2CD2 | 25966 | -1.278017208 | 1.46E-07 | 3.99E-06 |
| SNED1 | 25992 | -2.951958246 | 7.71E-34 | 7.67E-31 |
| CLIP3 | 25999 | -1.280524228 | 0.00737177 | 0.043107278 |
| PTPN20 | 26095 | -2.87083881 | 1.03E-08 | 3.57E-07 |
| LDLRAP1 | 26119 | -2.585303471 | 4.95E-13 | 3.79E-11 |
| ZNF658 | 26149 | -1.859093619 | 2.16E-04 | 0.002403198 |
| GAS6 | 2621 | -1.396113629 | 0.014344903 | 0.07249891 |
| PHGDH | 26227 | -2.803550755 | 1.34E-42 | 2.84E-39 |
| KLHL3 | 26249 | -1.763906562 | 2.51E-22 | 7.34E-20 |
| FBXO24 | 26261 | -1.712895464 | 0.011442038 | 0.060681168 |
| AK5 | 26289 | -3.436652543 | 1.95E-22 | 5.80E-20 |
| GAPDHS | 26330 | -2.139271041 | 0.011750835 | 0.061988586 |
| GCK | 2645 | -2.686485393 | 3.74E-04 | 0.003810099 |
| PLEK2 | 26499 | -4.507251017 | 0.013134109 | 0.067437214 |
| HEYL | 26508 | -4.874453358 | 0.042434078 | 0.164479143 |
| ITGB1BP2 | 26548 | -1.31095615 | 0.024989483 | 0.111464687 |
| BEST4 | 266675 | -1.178993278 | 9.24E-04 | 0.007996493 |
| OR4E1 | 26687 | -4.588609115 | 0.02918204 | 0.125113849 |
| GEM | 2669 | -3.602043491 | 2.12E-05 | 3.25E-04 |
| SH3YL1 | 26751 | -1.482223992 | 1.35E-11 | 7.86E-10 |
| HAVCR1 | 26762 | -3.984500133 | 1.98E-10 | 9.62E-09 |
| NBEA | 26960 | -3.451209333 | 3.83E-25 | 1.54E-22 |
| ZNF285 | 26974 | -1.679786513 | 0.03061906 | 0.129606391 |
| GPR160 | 26996 | -2.04532224 | 1.89E-06 | 3.79E-05 |
| BEX3 | 27018 | -3.391950175 | 5.52E-19 | 1.12E-16 |
| LYPD3 | 27076 | -3.769072839 | 1.09E-08 | 3.76E-07 |
| FOXP1 | 27086 | -1.287336736 | 1.15E-13 | 1.01E-11 |
| PDE7B | 27115 | -1.540354301 | 0.00814205 | 0.046707768 |
| CPAMD8 | 27151 | -2.890958026 | 0.005145206 | 0.032453275 |
| GLS2 | 27165 | -2.400899559 | 4.95E-08 | 1.51E-06 |
| IL36A | 27179 | -4.963982804 | 0.011163513 | 0.059652708 |
| DISC1 | 27185 | -1.941766645 | 5.12E-07 | 1.22E-05 |
| GPC3 | 2719 | -5.287549303 | 1.95E-09 | 7.78E-08 |
| GPR82 | 27197 | -1.062116721 | 7.21E-04 | 0.006500657 |
| RAB30 | 27314 | -1.166351278 | 7.38E-06 | 1.26E-04 |
| RPS6KA6 | 27330 | -1.306312214 | 0.028629555 | 0.1235019 |
| KCNMB4 | 27345 | -2.153517014 | 2.68E-05 | 3.96E-04 |
| GNA11 | 2767 | -1.76306513 | 0.001358899 | 0.011114857 |
| GNAI1 | 2770 | -3.885893239 | 3.40E-10 | 1.57E-08 |
| GNAQ | 2776 | -1.407997585 | 3.19E-07 | 7.96E-06 |
| GNG7 | 2788 | -1.296273386 | 5.83E-05 | 7.79E-04 |
| GP5 | 2814 | -3.088300966 | 3.00E-09 | 1.17E-07 |
| CCR10 | 2826 | -2.063425723 | 0.002617003 | 0.019023856 |
| ZNF311 | 282890 | -7.155884089 | 2.32E-09 | 9.18E-08 |
| RBM20 | 282996 | -2.886126002 | 8.23E-05 | 0.001048632 |
| MAJIN | 283129 | -4.572215146 | 0.002557339 | 0.018684365 |
| CCDC153 | 283152 | -2.905938182 | 0.002956134 | 0.020868569 |
| OLFML1 | 283298 | -3.391865643 | 3.92E-08 | 1.22E-06 |
| SIAH3 | 283514 | -5.127730525 | 0.001773076 | 0.013936724 |
| TMEM272 | 283521 | -7.409851255 | 6.13E-17 | 8.65E-15 |
| PROX2 | 283571 | -1.1841698 | 0.014410891 | 0.072733526 |
| TERB1 | 283847 | -4.478420265 | 0.033698394 | 0.13912611 |
| HID1 | 283987 | -1.684010868 | 4.17E-06 | 7.70E-05 |
| CCDC57 | 284001 | -1.420004184 | 2.08E-14 | 2.05E-12 |
| GPR18 | 2841 | -1.470662086 | 1.09E-06 | 2.36E-05 |
| ZIK1 | 284307 | -1.861863151 | 2.17E-06 | 4.27E-05 |
| ZNF776 | 284309 | -1.661887271 | 8.45E-08 | 2.45E-06 |
| NKPD1 | 284353 | -1.562308952 | 0.019750494 | 0.092715012 |
| ZNF844 | 284391 | -1.398499973 | 6.57E-05 | 8.64E-04 |
| OR2L13 | 284521 | -4.861184743 | 0.017714852 | 0.085629376 |
| NBPF15 | 284565 | -1.105190969 | 8.87E-05 | 0.001120593 |
| CCDC141 | 285025 | -2.560886134 | 1.96E-12 | 1.37E-10 |
| DLL1 | 28514 | -2.942868108 | 3.00E-12 | 2.00E-10 |
| RNF212 | 285498 | -1.857512365 | 6.40E-05 | 8.45E-04 |
| FAM153A | 285596 | -3.236746457 | 1.56E-21 | 4.32E-19 |
| RGMB | 285704 | -3.032173625 | 2.11E-12 | 1.46E-10 |
| ATG9B | 285973 | -2.370497866 | 1.94E-05 | 3.03E-04 |
| MICU3 | 286097 | -2.86910266 | 3.68E-05 | 5.19E-04 |
| GRB10 | 2887 | -3.226072398 | 1.37E-04 | 0.001626086 |
| GRIK1 | 2897 | -2.959533148 | 2.37E-05 | 3.57E-04 |
| N6AMT1 | 29104 | -1.299041528 | 8.26E-07 | 1.84E-05 |
| GSTM2 | 2946 | -2.764173808 | 5.40E-04 | 0.005135104 |
| GSTM3 | 2947 | -1.108374444 | 0.002634965 | 0.019097006 |
| TRPM5 | 29850 | -4.412873816 | 0.001839717 | 0.014347252 |
| FAM216A | 29902 | -1.264355237 | 9.75E-11 | 4.94E-09 |
| CCDC106 | 29903 | -2.647808661 | 2.10E-06 | 4.16E-05 |
| SLC40A1 | 30061 | -2.620364935 | 1.47E-07 | 4.02E-06 |
| SOX8 | 30812 | -3.047472108 | 0.002447823 | 0.01802633 |
| HIVEP1 | 3096 | -1.252636211 | 5.40E-05 | 7.28E-04 |
| HK2 | 3099 | -1.848060778 | 3.03E-05 | 4.39E-04 |
| HLA-DOA | 3111 | -1.785384582 | 1.75E-14 | 1.78E-12 |
| APBA2 | 321 | -1.873958582 | 3.31E-24 | 1.19E-21 |
| APBB1 | 322 | -1.206590481 | 6.62E-18 | 1.17E-15 |
| HRH2 | 3274 | -2.932030792 | 1.47E-06 | 3.02E-05 |
| HSF2 | 3298 | -1.085064533 | 1.61E-05 | 2.56E-04 |
| HSPG2 | 3339 | -5.161185636 | 4.35E-12 | 2.78E-10 |
| TAS2R60 | 338398 | -5.578685402 | 1.55E-05 | 2.48E-04 |
| RAB43 | 339122 | -1.463505536 | 1.80E-05 | 2.83E-04 |
| ZNF546 | 339327 | -1.727365093 | 1.89E-11 | 1.08E-09 |
| RXFP4 | 339403 | -1.98813875 | 0.018338373 | 0.087978274 |
| ARMH1 | 339541 | -3.348715682 | 3.66E-13 | 2.92E-11 |
| ID3 | 3399 | -2.32332634 | 0.004656136 | 0.029971772 |
| TSPAN33 | 340348 | -1.097286035 | 0.04311232 | 0.166004504 |
| AGBL3 | 340351 | -1.538486174 | 2.48E-06 | 4.83E-05 |
| VSIG1 | 340547 | -1.472738706 | 2.48E-11 | 1.39E-09 |
| ZC3H12B | 340554 | -1.120027328 | 8.22E-04 | 0.007256718 |
| GOLGA6A | 342096 | -3.20052141 | 0.022555475 | 0.103108132 |
| C16orf96 | 342346 | -2.824502723 | 0.043400532 | 0.166891973 |
| IDUA | 3425 | -1.154379423 | 0.026951042 | 0.117792479 |
| ZNF677 | 342926 | -1.186779509 | 1.23E-04 | 0.001484159 |
| SH3RF3 | 344558 | -5.695793649 | 1.26E-51 | 3.54E-48 |
| IFNGR2 | 3460 | -2.431757647 | 1.27E-11 | 7.44E-10 |
| ZNF391 | 346157 | -1.978736808 | 2.05E-08 | 6.77E-07 |
| OR2A1 | 346528 | -1.322149107 | 5.04E-04 | 0.004844609 |
| KLRG2 | 346689 | -4.004593609 | 0.030520338 | 0.129317899 |
| IGF1R | 3480 | -2.700116282 | 8.91E-28 | 5.03E-25 |
| FTCDNL1 | 348751 | -1.454678511 | 2.51E-04 | 0.002700094 |
| IGFBP6 | 3489 | -1.350029102 | 0.0063157 | 0.038239927 |
| NIPAL4 | 348938 | -4.872374816 | 0.041846366 | 0.162759378 |
| WDR86 | 349136 | -1.06345783 | 0.008725577 | 0.049451757 |
| NMNAT3 | 349565 | -2.62263971 | 4.61E-05 | 6.35E-04 |
| APP | 351 | -2.058648944 | 0.0113093 | 0.060165762 |
| IL3RA | 3563 | -1.323536784 | 0.021490968 | 0.0990799 |
| IL6R | 3570 | -2.531894964 | 0.001651891 | 0.013075279 |
| IL6ST | 3572 | -2.547533066 | 2.07E-29 | 1.40E-26 |
| IL9R | 3581 | -1.1483721 | 0.020716526 | 0.096274079 |
| IL11RA | 3590 | -1.009632046 | 4.66E-04 | 0.004560088 |
| ITGA6 | 3655 | -1.994799957 | 6.98E-14 | 6.39E-12 |
| IRS1 | 3667 | -2.729182426 | 3.91E-08 | 1.22E-06 |
| ITGA9 | 3680 | -4.30766608 | 0.014276535 | 0.072206156 |
| ITPKB | 3707 | -1.661893582 | 2.76E-09 | 1.08E-07 |
| AREG | 374 | -1.140763012 | 0.01086886 | 0.058540331 |
| CDRT1 | 374286 | -1.348059898 | 0.002810957 | 0.020088611 |
| DNAJB13 | 374407 | -1.707744985 | 0.005220594 | 0.032879835 |
| TEPP | 374739 | -1.707973191 | 0.008600521 | 0.048923233 |
| YJEFN3 | 374887 | -1.036973465 | 0.003399004 | 0.023287996 |
| ZNF773 | 374928 | -1.400326917 | 1.86E-04 | 0.002101487 |
| MAST4 | 375449 | -1.23896659 | 2.90E-08 | 9.18E-07 |
| KCNH2 | 3757 | -1.790820441 | 0.011196513 | 0.059786481 |
| PNPLA7 | 375775 | -1.061179362 | 0.012761861 | 0.066065763 |
| KCNJ12 | 3768 | -4.941130653 | 0.002555408 | 0.018684365 |
| KCNJ14 | 3770 | -1.281783279 | 0.002822177 | 0.020160281 |
| KCNN4 | 3783 | -1.497542841 | 3.92E-04 | 0.003952075 |
| KCNQ1 | 3784 | -2.24850523 | 9.33E-10 | 3.99E-08 |
| NHLRC1 | 378884 | -1.603894663 | 0.02356197 | 0.106415591 |
| KRT2 | 3849 | -1.523993199 | 0.002314324 | 0.017245844 |
| KRT17 | 3872 | -2.336699532 | 0.011978388 | 0.062816938 |
| RASL11A | 387496 | -1.176225565 | 0.041621833 | 0.162072314 |
| KRT18 | 3875 | -1.032480379 | 7.23E-05 | 9.36E-04 |
| SHISA2 | 387914 | -3.69450574 | 2.65E-04 | 0.002836245 |
| C17orf100 | 388327 | -1.163020168 | 8.42E-04 | 0.007392367 |
| TMEM220 | 388335 | -3.21584773 | 3.01E-27 | 1.59E-24 |
| LRRC75A | 388341 | -1.094289083 | 6.58E-13 | 4.95E-11 |
| YPEL2 | 388403 | -2.072304926 | 7.22E-26 | 3.22E-23 |
| LYSMD1 | 388695 | -1.022847329 | 0.017806263 | 0.085854904 |
| C2orf81 | 388963 | -2.037825666 | 2.39E-05 | 3.59E-04 |
| ZNF662 | 389114 | -2.672045158 | 1.00E-12 | 7.35E-11 |
| LEKR1 | 389170 | -1.126338579 | 0.02869057 | 0.123583534 |
| NUPR2 | 389493 | -3.015065891 | 0.003230868 | 0.022362366 |
| LDHB | 3945 | -1.44612162 | 8.97E-17 | 1.24E-14 |
| LGALS3BP | 3959 | -1.846762931 | 5.95E-07 | 1.39E-05 |
| ABLIM1 | 3983 | -1.922349041 | 1.81E-17 | 2.95E-15 |
| RHOH | 399 | -1.032109212 | 3.57E-05 | 5.07E-04 |
| FAM102A | 399665 | -1.94697812 | 6.70E-12 | 4.12E-10 |
| SMIM10L2A | 399668 | -1.76830191 | 0.01617317 | 0.079517302 |
| HEATR4 | 399671 | -1.41261701 | 0.006509249 | 0.039090139 |
| FAM174B | 400451 | -2.536763789 | 0.001213377 | 0.010105301 |
| GRAPL | 400581 | -1.24288882 | 1.28E-05 | 2.08E-04 |
| LMO7 | 4008 | -2.24690978 | 3.41E-32 | 3.04E-29 |
| NBPF9 | 400818 | -1.520050288 | 4.03E-07 | 9.92E-06 |
| PAIP2B | 400961 | -1.168801613 | 3.74E-04 | 0.003810099 |
| FSIP2 | 401024 | -3.669727207 | 2.68E-11 | 1.49E-09 |
| FOXL2NB | 401089 | -1.685752453 | 2.18E-04 | 0.002414752 |
| C5orf63 | 401207 | -1.075038628 | 1.96E-05 | 3.04E-04 |
| OR2A42 | 402317 | -2.635482668 | 5.39E-05 | 7.27E-04 |
| XKRX | 402415 | -1.112360125 | 9.14E-05 | 0.00115036 |
| PLPP6 | 403313 | -1.153283223 | 1.82E-08 | 6.05E-07 |
| LRP6 | 4040 | -4.819228512 | 3.30E-12 | 2.17E-10 |
| C16orf74 | 404550 | -2.555147907 | 3.27E-15 | 3.71E-13 |
| LTBP3 | 4054 | -1.215432412 | 3.67E-05 | 5.19E-04 |
| MAB21L1 | 4081 | -3.310975179 | 0.009043852 | 0.050762555 |
| FNDC9 | 408263 | -1.494864469 | 0.002349596 | 0.017447222 |
| MAFG | 4097 | -1.081789754 | 0.004871103 | 0.03108359 |
| ASIC1 | 41 | -3.806072258 | 3.38E-08 | 1.07E-06 |
| MAL | 4118 | -4.736623766 | 4.79E-29 | 2.89E-26 |
| LCN10 | 414332 | -2.111577535 | 0.003282834 | 0.022675674 |
| MEST | 4232 | -3.088872115 | 1.14E-23 | 3.78E-21 |
| MFGE8 | 4240 | -1.836583979 | 1.43E-06 | 2.96E-05 |
| MGP | 4256 | -2.201794999 | 0.001163951 | 0.009760922 |
| MLLT3 | 4300 | -1.048298791 | 6.47E-07 | 1.50E-05 |
| NR3C2 | 4306 | -1.536607305 | 2.21E-11 | 1.25E-09 |
| MME | 4311 | -4.799934983 | 0.022687239 | 0.103431074 |
| MMP19 | 4327 | -1.106340414 | 1.75E-07 | 4.63E-06 |
| MPP2 | 4355 | -1.713039048 | 7.12E-05 | 9.24E-04 |
| IQSEC3 | 440073 | -3.592140273 | 0.018861992 | 0.089792557 |
| GPR179 | 440435 | -5.530441891 | 0.019369366 | 0.091535265 |
| HSBP1L1 | 440498 | -2.40522446 | 1.35E-08 | 4.56E-07 |
| CDNF | 441549 | -1.484573008 | 0.009726619 | 0.053774445 |
| MSH2 | 4436 | -1.015917598 | 7.25E-11 | 3.76E-09 |
| MT3 | 4504 | -4.842143251 | 0.018118191 | 0.087180594 |
| MYB | 4602 | -4.970207507 | 4.61E-16 | 5.73E-14 |
| MYC | 4609 | -1.765728944 | 3.52E-09 | 1.36E-07 |
| MYH3 | 4621 | -1.397126721 | 1.29E-04 | 0.001549052 |
| MYO7A | 4647 | -6.014662711 | 7.38E-05 | 9.53E-04 |
| MYO10 | 4651 | -4.038534936 | 1.11E-13 | 9.82E-12 |
| NAP1L3 | 4675 | -2.017460519 | 4.45E-06 | 8.14E-05 |
| NELL2 | 4753 | -3.160371936 | 1.56E-14 | 1.59E-12 |
| NKX3-1 | 4824 | -1.841413815 | 1.97E-05 | 3.06E-04 |
| NOS3 | 4846 | -1.134332773 | 0.005660825 | 0.035026741 |
| FXYD2 | 486 | -1.956860627 | 7.19E-06 | 1.23E-04 |
| NPAS2 | 4862 | -3.446358832 | 3.16E-09 | 1.23E-07 |
| NRCAM | 4897 | -6.424250219 | 7.96E-41 | 1.35E-37 |
| NT5E | 4907 | -3.171384222 | 5.52E-36 | 6.68E-33 |
| NUCB2 | 4925 | -1.356796122 | 1.29E-15 | 1.52E-13 |
| PJVK | 494513 | -1.112816884 | 0.001037079 | 0.0088371 |
| ALDH7A1 | 501 | -3.162945303 | 1.65E-04 | 0.001902408 |
| OVGP1 | 5016 | -1.474482342 | 3.74E-04 | 0.003810099 |
| PCSK6 | 5046 | -4.108590867 | 1.42E-04 | 0.001680717 |
| UBQLN3 | 50613 | -3.547287479 | 0.044508156 | 0.169948813 |
| ARHGEF4 | 50649 | -2.612995292 | 1.99E-08 | 6.57E-07 |
| PAPPA | 5069 | -1.154359933 | 0.031966674 | 0.133970289 |
| PRKN | 5071 | -1.277110199 | 1.29E-06 | 2.73E-05 |
| PC | 5091 | -1.183174677 | 0.003308336 | 0.022814576 |
| SHANK1 | 50944 | -4.638306869 | 4.68E-06 | 8.52E-05 |
| NOSIP | 51070 | -1.529124595 | 1.40E-20 | 3.44E-18 |
| PLLP | 51090 | -4.224049824 | 2.63E-05 | 3.90E-04 |
| ZC2HC1A | 51101 | -1.001956574 | 0.044318117 | 0.169528819 |
| KCTD3 | 51133 | -3.615653624 | 3.21E-06 | 6.08E-05 |
| HSD17B14 | 51171 | -2.071763648 | 0.005147113 | 0.032453275 |
| LEF1 | 51176 | -3.884151434 | 3.35E-54 | 1.13E-50 |
| TCEAL9 | 51186 | -2.101227881 | 0.003300074 | 0.022766881 |
| PCSK5 | 5125 | -2.327883887 | 4.69E-13 | 3.61E-11 |
| FAM178B | 51252 | -1.158656151 | 0.024043723 | 0.108043526 |
| CDK18 | 5129 | -1.329366865 | 0.007448192 | 0.043464054 |
| SLC15A3 | 51296 | -1.913267434 | 0.009507567 | 0.052770272 |
| GCNT4 | 51301 | -2.781058395 | 6.25E-12 | 3.86E-10 |
| ARMCX1 | 51309 | -4.364119698 | 4.39E-11 | 2.34E-09 |
| SLC22A17 | 51310 | -3.580963313 | 1.26E-07 | 3.52E-06 |
| HOOK1 | 51361 | -2.489638005 | 2.75E-12 | 1.84E-10 |
| PDE3B | 5140 | -1.511344008 | 1.19E-04 | 0.00144597 |
| SNX9 | 51429 | -1.384363797 | 1.00E-03 | 0.008569955 |
| HPCAL4 | 51440 | -3.007088375 | 1.80E-12 | 1.26E-10 |
| RNF138 | 51444 | -1.03912999 | 0.001949545 | 0.01503059 |
| SFMBT1 | 51460 | -1.079831765 | 1.43E-05 | 2.30E-04 |
| PDE6G | 5148 | -2.203139096 | 2.07E-04 | 0.002314788 |
| PDE7A | 5150 | -1.13860219 | 3.46E-06 | 6.50E-05 |
| PDE9A | 5152 | -2.87787587 | 3.98E-07 | 9.83E-06 |
| LSR | 51599 | -1.715684034 | 1.04E-04 | 0.001278648 |
| PDK1 | 5163 | -2.425627366 | 2.78E-30 | 2.05E-27 |
| ENPP2 | 5168 | -4.589853223 | 1.68E-08 | 5.62E-07 |
| RAPGEF6 | 51735 | -1.216081999 | 3.47E-18 | 6.46E-16 |
| SERPINF1 | 5176 | -3.015662592 | 2.22E-06 | 4.36E-05 |
| ATP8A2 | 51761 | -1.695863752 | 4.84E-06 | 8.76E-05 |
| PEX12 | 5193 | -1.147131246 | 0.001877255 | 0.014586223 |
| CFP | 5199 | -2.733598145 | 5.08E-06 | 9.13E-05 |
| PFKFB2 | 5208 | -1.673612145 | 4.58E-04 | 0.00449725 |
| PFN2 | 5217 | -1.242251237 | 1.35E-05 | 2.19E-04 |
| SERPINE2 | 5270 | -5.04184931 | 3.34E-13 | 2.68E-11 |
| PLAG1 | 5324 | -4.664573003 | 1.85E-34 | 2.09E-31 |
| SPA17 | 53340 | -1.343947204 | 0.036252911 | 0.146877666 |
| TPCN1 | 53373 | -1.387489965 | 4.75E-11 | 2.52E-09 |
| PLXNB3 | 5365 | -4.582539099 | 0.031184477 | 0.131637543 |
| RBM11 | 54033 | -4.512329165 | 2.17E-24 | 8.16E-22 |
| RIPK4 | 54101 | -5.8102904 | 4.51E-09 | 1.70E-07 |
| TLR9 | 54106 | -1.477020166 | 1.25E-05 | 2.04E-04 |
| SNTG2 | 54221 | -4.308411758 | 1.21E-09 | 5.11E-08 |
| SIAE | 54414 | -1.689122618 | 4.49E-12 | 2.85E-10 |
| SEMA5B | 54437 | -5.856570721 | 7.02E-04 | 0.006366645 |
| RETREG1 | 54463 | -2.657794269 | 7.34E-10 | 3.17E-08 |
| NLE1 | 54475 | -1.083542249 | 1.03E-05 | 1.70E-04 |
| USP53 | 54532 | -1.32793029 | 6.74E-09 | 2.41E-07 |
| SDK2 | 54549 | -4.103793219 | 0.007761678 | 0.04499859 |
| SGTB | 54557 | -1.329811897 | 3.43E-05 | 4.91E-04 |
| SPATA6 | 54558 | -2.162200026 | 1.95E-05 | 3.04E-04 |
| MXRA8 | 54587 | -6.049970513 | 1.21E-33 | 1.13E-30 |
| MAP10 | 54627 | -1.162089427 | 0.012697034 | 0.065871214 |
| PPARD | 5467 | -1.054764018 | 9.55E-06 | 1.60E-04 |
| LRRN3 | 54674 | -5.984788476 | 5.94E-92 | 3.35E-88 |
| LY6K | 54742 | -3.707798272 | 0.010448719 | 0.056747006 |
| PPEF1 | 5475 | -2.766972249 | 0.011566679 | 0.061167574 |
| SPATA6L | 55064 | -1.405840776 | 1.40E-06 | 2.91E-05 |
| UACA | 55075 | -2.122285146 | 2.03E-05 | 3.13E-04 |
| RALGPS2 | 55103 | -1.292074684 | 1.24E-04 | 0.001497569 |
| TMEM38B | 55151 | -1.008018167 | 1.23E-04 | 0.001488644 |
| DPPA4 | 55211 | -4.994406289 | 9.36E-05 | 0.001169728 |
| HEMGN | 55363 | -2.923993217 | 5.77E-08 | 1.74E-06 |
| SIRPG | 55423 | -1.09353457 | 1.02E-04 | 0.001259075 |
| MAML3 | 55534 | -2.610509883 | 2.43E-09 | 9.56E-08 |
| CDCA7L | 55536 | -2.529617121 | 7.49E-18 | 1.29E-15 |
| CARMIL1 | 55604 | -3.188841206 | 4.36E-10 | 1.99E-08 |
| ASAP3 | 55616 | -2.45039436 | 8.42E-05 | 0.001069106 |
| DEPDC1 | 55635 | -1.801094012 | 0.026949808 | 0.117792479 |
| RAB20 | 55647 | -1.429313238 | 0.003867109 | 0.025826001 |
| BCAS4 | 55653 | -1.631387899 | 1.60E-12 | 1.14E-10 |
| LIMS2 | 55679 | -3.013665157 | 1.74E-09 | 7.04E-08 |
| PKIA | 5569 | -1.904326674 | 3.37E-04 | 0.003489013 |
| PKIB | 5570 | -4.407816342 | 0.004851108 | 0.031002757 |
| ZNF334 | 55713 | -4.409299603 | 0.011843901 | 0.062324706 |
| PRKAR1B | 5575 | -1.907901586 | 1.70E-17 | 2.80E-15 |
| DHX32 | 55760 | -1.089522246 | 0.013427182 | 0.068649003 |
| PRKCA | 5578 | -2.342766073 | 9.23E-13 | 6.82E-11 |
| ZNF415 | 55786 | -2.348122942 | 1.26E-05 | 2.06E-04 |
| CSGALNACT1 | 55790 | -2.597911408 | 3.30E-16 | 4.26E-14 |
| MINDY1 | 55793 | -1.984762006 | 2.80E-13 | 2.27E-11 |
| ADCY10 | 55811 | -1.240517979 | 0.015382375 | 0.07669886 |
| TSNAXIP1 | 55815 | -2.121686364 | 0.001639278 | 0.012999766 |
| ZNF302 | 55900 | -1.0306938 | 5.06E-08 | 1.54E-06 |
| ACSS2 | 55902 | -1.441368459 | 3.91E-06 | 7.26E-05 |
| MAPK11 | 5600 | -4.142938707 | 0.011792398 | 0.062122967 |
| PRL | 5617 | -7.093550421 | 5.13E-08 | 1.56E-06 |
| CPXM1 | 56265 | -4.210423937 | 0.001483137 | 0.01194628 |
| BEX4 | 56271 | -1.219246751 | 3.52E-05 | 5.01E-04 |
| LGMN | 5641 | -2.272677036 | 4.37E-13 | 3.41E-11 |
| PRSS1 | 5644 | -1.321575609 | 0.002254063 | 0.016900975 |
| TWNK | 56652 | -1.130971455 | 1.29E-06 | 2.73E-05 |
| KCNK12 | 56660 | -2.231762667 | 1.02E-04 | 0.00126484 |
| RETN | 56729 | -4.266952279 | 0.001423086 | 0.011545009 |
| SEMA3G | 56920 | -4.018077874 | 0.018457254 | 0.088409879 |
| PMEPA1 | 56937 | -2.215944334 | 9.10E-04 | 0.007900663 |
| C14orf132 | 56967 | -1.959590164 | 0.006506274 | 0.039086145 |
| ADPRM | 56985 | -2.043920473 | 1.09E-13 | 9.64E-12 |
| CASS4 | 57091 | -1.207679092 | 2.08E-04 | 0.002319692 |
| CD248 | 57124 | -4.47560745 | 2.27E-26 | 1.07E-23 |
| PLXDC1 | 57125 | -1.652322112 | 1.31E-06 | 2.74E-05 |
| MAN1C1 | 57134 | -2.532211867 | 1.12E-22 | 3.37E-20 |
| ANKRD50 | 57182 | -2.579608074 | 0.030858956 | 0.130556529 |
| VN1R1 | 57191 | -1.778927059 | 0.006705694 | 0.040038424 |
| ADGRG6 | 57211 | -5.117203072 | 0.001915949 | 0.014815022 |
| RCN3 | 57333 | -2.385961856 | 6.06E-09 | 2.20E-07 |
| PLEKHG5 | 57449 | -1.203663389 | 0.010070894 | 0.055226744 |
| AMIGO1 | 57463 | -2.279544403 | 3.34E-14 | 3.20E-12 |
| NLN | 57486 | -1.867290171 | 7.43E-07 | 1.68E-05 |
| MTA3 | 57504 | -1.000646915 | 1.02E-06 | 2.23E-05 |
| MTUS1 | 57509 | -2.288269068 | 8.01E-09 | 2.85E-07 |
| SRGAP1 | 57522 | -1.480063698 | 6.41E-09 | 2.31E-07 |
| CGN | 57530 | -1.253383885 | 0.01656904 | 0.081039845 |
| PTK7 | 5754 | -4.035154096 | 0.007976992 | 0.045994764 |
| NLGN2 | 57555 | -1.022070754 | 0.027917134 | 0.121045776 |
| PITPNM2 | 57605 | -1.382407377 | 1.04E-06 | 2.27E-05 |
| DIP2B | 57609 | -1.17344277 | 1.69E-10 | 8.27E-09 |
| GRAMD1A | 57655 | -1.460809328 | 1.84E-09 | 7.36E-08 |
| PLEKHA4 | 57664 | -1.618387601 | 5.46E-04 | 0.00517218 |
| CACHD1 | 57685 | -6.629409477 | 1.90E-19 | 4.17E-17 |
| WDR19 | 57728 | -1.173773837 | 9.74E-04 | 0.008374763 |
| SLC4A5 | 57835 | -1.010611269 | 2.91E-04 | 0.003068416 |
| PTPRK | 5796 | -3.755786688 | 7.49E-27 | 3.62E-24 |
| PTPRO | 5800 | -1.776291501 | 0.002981489 | 0.020988245 |
| PTPRS | 5802 | -2.226301755 | 4.92E-08 | 1.50E-06 |
| PVR | 5817 | -2.155583918 | 0.003116079 | 0.021754705 |
| CXCL16 | 58191 | -1.291276425 | 8.57E-04 | 0.007502011 |
| PLEKHB1 | 58473 | -1.70563348 | 3.37E-21 | 8.52E-19 |
| RAB3A | 5864 | -1.827952836 | 1.50E-07 | 4.07E-06 |
| RASGRF2 | 5924 | -1.495603796 | 1.28E-07 | 3.58E-06 |
| ALOXE3 | 59344 | -2.720717105 | 0.044599768 | 0.170221443 |
| RENBP | 5973 | -1.992864308 | 0.004945647 | 0.03148803 |
| RFX2 | 5990 | -4.220710727 | 8.71E-18 | 1.49E-15 |
| RGS10 | 6001 | -2.080293861 | 1.86E-24 | 7.15E-22 |
| CDH26 | 60437 | -1.484694696 | 0.010206007 | 0.055726531 |
| BACH2 | 60468 | -2.521173728 | 4.30E-24 | 1.49E-21 |
| BCL7A | 605 | -1.253701057 | 1.84E-05 | 2.89E-04 |
| BCL9 | 607 | -2.403345628 | 2.46E-25 | 1.04E-22 |
| ROBO1 | 6091 | -4.500380151 | 3.42E-04 | 0.00353967 |
| FAM229B | 619208 | -1.57734788 | 3.06E-04 | 0.003210645 |
| MBOAT4 | 619373 | -1.435246819 | 9.60E-06 | 1.60E-04 |
| BDNF | 627 | -2.769504277 | 0.001610767 | 0.012803668 |
| SORT1 | 6272 | -3.359130186 | 2.26E-05 | 3.44E-04 |
| S100B | 6285 | -3.408845556 | 0.012271967 | 0.064019263 |
| ACSM3 | 6296 | -1.584515802 | 2.79E-05 | 4.10E-04 |
| SLC22A23 | 63027 | -1.50052632 | 3.69E-06 | 6.89E-05 |
| SATB1 | 6304 | -1.59548142 | 1.54E-13 | 1.29E-11 |
| CLEC11A | 6320 | -4.562643764 | 2.69E-34 | 2.84E-31 |
| SCML1 | 6322 | -3.672383701 | 9.06E-30 | 6.39E-27 |
| SCNN1D | 6339 | -1.086476745 | 1.66E-04 | 0.001915242 |
| ZNF667 | 63934 | -3.982158528 | 3.76E-14 | 3.58E-12 |
| SELL | 6402 | -2.075815131 | 1.00E-13 | 8.97E-12 |
| CDH23 | 64072 | -1.851265117 | 5.21E-17 | 7.53E-15 |
| DPEP2 | 64174 | -1.171332678 | 5.08E-08 | 1.54E-06 |
| DPEP3 | 64180 | -1.610346573 | 0.011391347 | 0.060469185 |
| ROBO3 | 64221 | -1.085314051 | 0.010366551 | 0.056427515 |
| ABCG8 | 64241 | -1.791516339 | 0.007342798 | 0.042967558 |
| SFRP5 | 6425 | -5.380731782 | 2.48E-17 | 3.82E-15 |
| TMEM232 | 642987 | -2.310104976 | 4.68E-04 | 0.004570089 |
| ZFP62 | 643836 | -1.043853688 | 9.64E-14 | 8.67E-12 |
| AKTIP | 64400 | -1.263361635 | 1.44E-07 | 3.95E-06 |
| INF2 | 64423 | -2.371470748 | 3.18E-08 | 1.00E-06 |
| SGK1 | 6446 | -1.704719242 | 0.009874686 | 0.054450736 |
| SH3BGR | 6450 | -2.019438102 | 1.18E-04 | 0.001440507 |
| CPEB1 | 64506 | -1.880225266 | 0.016318955 | 0.079978473 |
| CCDC154 | 645811 | -1.925845613 | 0.028688131 | 0.123583534 |
| SERPINE3 | 647174 | -1.705993678 | 7.51E-04 | 0.006728467 |
| DEPTOR | 64798 | -1.111929975 | 9.28E-05 | 0.001163074 |
| NDRG4 | 65009 | -3.032653229 | 8.40E-05 | 0.001066803 |
| SLC2A3 | 6515 | -1.073690537 | 0.005625848 | 0.034863 |
| SLC3A1 | 6519 | -1.813576755 | 0.028850648 | 0.124101277 |
| SLC5A2 | 6524 | -2.545553524 | 6.17E-05 | 8.17E-04 |
| SLC5A5 | 6528 | -1.585707939 | 6.01E-04 | 0.005609364 |
| PHLDB3 | 653583 | -1.469031675 | 2.48E-07 | 6.33E-06 |
| OCM | 654231 | -2.961266587 | 1.02E-07 | 2.89E-06 |
| SLC7A4 | 6545 | -2.635721822 | 0.001122762 | 0.009500247 |
| SLC9A3 | 6550 | -1.01483155 | 7.17E-06 | 1.23E-04 |
| SLC18A2 | 6571 | -6.429909476 | 4.85E-06 | 8.78E-05 |
| SMARCA1 | 6594 | -5.094105243 | 4.02E-04 | 0.004030142 |
| STK33 | 65975 | -1.41237408 | 0.018370288 | 0.088048374 |
| ZSCAN18 | 65982 | -2.464815786 | 0.014575557 | 0.073389515 |
| ZBTB10 | 65986 | -1.316066377 | 4.87E-04 | 0.004718584 |
| ANTKMT | 65990 | -1.245915336 | 6.08E-06 | 1.06E-04 |
| TMEM108 | 66000 | -5.432307276 | 5.47E-04 | 0.005179988 |
| SMARCD3 | 6604 | -2.445313887 | 4.79E-05 | 6.55E-04 |
| SMPD1 | 6609 | -1.152778719 | 2.11E-04 | 0.002349085 |
| BNIP3 | 664 | -1.24168184 | 3.06E-13 | 2.47E-11 |
| SOX4 | 6659 | -4.465940542 | 8.01E-19 | 1.59E-16 |
| SPINK2 | 6691 | -4.373784996 | 9.28E-10 | 3.97E-08 |
| SREBF1 | 6720 | -1.268190893 | 3.94E-05 | 5.52E-04 |
| SSTR3 | 6753 | -3.425126107 | 1.89E-14 | 1.90E-12 |
| STXBP1 | 6812 | -1.258586265 | 5.74E-04 | 0.0053861 |
| TAF4B | 6875 | -1.981588008 | 5.11E-09 | 1.90E-07 |
| KLF5 | 688 | -1.379385681 | 0.012658276 | 0.065723142 |
| TBXA2R | 6915 | -1.74910058 | 5.06E-16 | 6.25E-14 |
| TCEA3 | 6920 | -3.596968915 | 2.09E-31 | 1.68E-28 |
| TCF7 | 6932 | -1.870669608 | 4.20E-15 | 4.58E-13 |
| BUB1 | 699 | -1.775619772 | 7.28E-06 | 1.25E-04 |
| TEC | 7006 | -1.366588114 | 8.88E-04 | 0.007735888 |
| TERT | 7015 | -5.159872189 | 0.001132511 | 0.009563614 |
| TFAP4 | 7023 | -1.561616973 | 0.027294615 | 0.118986802 |
| LEFTY2 | 7044 | -4.01825153 | 0.007743864 | 0.044926089 |
| TGFBR2 | 7048 | -1.832400664 | 8.67E-13 | 6.47E-11 |
| THBS4 | 7060 | -1.749208458 | 0.030552898 | 0.129423452 |
| TIAM1 | 7074 | -1.704786554 | 5.46E-04 | 0.00517218 |
| TIMP2 | 7077 | -3.167856017 | 1.92E-06 | 3.84E-05 |
| TLR5 | 7100 | -1.839067793 | 0.002062558 | 0.015751206 |
| TNNC1 | 7134 | -2.282670924 | 1.95E-04 | 0.002198571 |
| TNNT3 | 7140 | -3.314561876 | 9.83E-07 | 2.16E-05 |
| TNXB | 7148 | -1.32451258 | 0.001264224 | 0.010466955 |
| C4BPB | 725 | -1.338925386 | 0.039640712 | 0.156518846 |
| C5 | 727 | -1.267825757 | 0.016211065 | 0.079634208 |
| TTN | 7273 | -1.845093956 | 1.70E-09 | 6.94E-08 |
| TUB | 7275 | -1.761564019 | 0.03684185 | 0.148871686 |
| MAGED4 | 728239 | -2.171005169 | 2.42E-04 | 0.002629591 |
| GOLGA6L7 | 728310 | -3.013491262 | 0.004230561 | 0.02786835 |
| DMRTC1B | 728656 | -2.316962913 | 0.004348342 | 0.028411967 |
| NBPF8 | 728841 | -1.121298483 | 5.83E-07 | 1.37E-05 |
| TXK | 7294 | -2.931698132 | 5.70E-68 | 2.41E-64 |
| FHIP1A | 729830 | -2.691532908 | 9.27E-05 | 0.001163074 |
| ZNF814 | 730051 | -1.069770973 | 1.08E-07 | 3.04E-06 |
| UBE2E2 | 7325 | -1.439556864 | 5.54E-08 | 1.68E-06 |
| UCP1 | 7350 | -1.719808286 | 0.024717982 | 0.110603495 |
| VIPR1 | 7433 | -2.412946554 | 7.62E-06 | 1.30E-04 |
| ZAN | 7455 | -4.924632264 | 0.011526925 | 0.061016628 |
| WNT7A | 7476 | -2.234960647 | 0.00354383 | 0.024104594 |
| LDLRAD4 | 753 | -1.027340166 | 1.28E-06 | 2.71E-05 |
| ZNF711 | 7552 | -1.816908558 | 0.011053769 | 0.059193839 |
| ZNF10 | 7556 | -1.899546021 | 4.68E-06 | 8.52E-05 |
| ZNF90 | 7643 | -1.335847738 | 0.022800866 | 0.103805511 |
| CA6 | 765 | -5.202460141 | 4.81E-06 | 8.72E-05 |
| MKRN3 | 7681 | -1.481494027 | 0.048006672 | 0.179897457 |
| PRCD | 768206 | -1.021654813 | 0.002199004 | 0.016561522 |
| ZNF135 | 7694 | -4.128076308 | 0.004987199 | 0.031681155 |
| ZNF154 | 7710 | -1.976579646 | 3.16E-14 | 3.03E-12 |
| ZNF208 | 7757 | -1.225133135 | 0.031490116 | 0.132432217 |
| ZNF223 | 7766 | -2.805280029 | 4.50E-13 | 3.48E-11 |
| ZNF112 | 7771 | -3.826086678 | 0.030462712 | 0.129203129 |
| ZNF229 | 7772 | -4.582209936 | 5.76E-08 | 1.74E-06 |
| CACNA1F | 778 | -1.386022892 | 0.005753457 | 0.035521983 |
| ZP3 | 7784 | -2.628391297 | 1.87E-06 | 3.77E-05 |
| PCYOX1L | 78991 | -1.474081954 | 3.35E-12 | 2.20E-10 |
| DBNDD1 | 79007 | -2.752956415 | 2.71E-04 | 0.002888436 |
| MMP28 | 79148 | -4.850988049 | 1.91E-09 | 7.61E-08 |
| MMEL1 | 79258 | -2.349321724 | 0.003439407 | 0.023517209 |
| BCL2L14 | 79370 | -2.919654136 | 0.037237665 | 0.150090216 |
| ZBED2 | 79413 | -1.109381281 | 3.99E-08 | 1.24E-06 |
| BIRC7 | 79444 | -2.875637866 | 2.58E-05 | 3.83E-04 |
| EPM2A | 7957 | -1.113231737 | 4.25E-04 | 0.00421799 |
| RIC3 | 79608 | -1.556297267 | 9.44E-13 | 6.95E-11 |
| NAA16 | 79612 | -1.604517857 | 4.24E-10 | 1.94E-08 |
| FAM184A | 79632 | -1.889534001 | 0.005337429 | 0.033429054 |
| CCDC121 | 79635 | -1.754736468 | 0.029877528 | 0.127488001 |
| TMEM204 | 79652 | -1.614978574 | 5.23E-06 | 9.35E-05 |
| BEND5 | 79656 | -3.360135392 | 2.09E-08 | 6.85E-07 |
| RTL10 | 79680 | -1.013689245 | 7.28E-07 | 1.66E-05 |
| GAL3ST4 | 79690 | -4.083953514 | 2.65E-37 | 4.08E-34 |
| ANKRD55 | 79722 | -3.655355735 | 2.83E-05 | 4.15E-04 |
| SH3D21 | 79729 | -1.347440861 | 0.004797446 | 0.030729437 |
| NSUN7 | 79730 | -2.354170328 | 0.02316488 | 0.105042676 |
| ASB13 | 79754 | -1.374089489 | 2.04E-06 | 4.06E-05 |
| IQCA1 | 79781 | -2.482617973 | 3.21E-05 | 4.62E-04 |
| SUGCT | 79783 | -2.172951603 | 0.002855322 | 0.020345521 |
| AGMAT | 79814 | -1.485625848 | 5.72E-09 | 2.09E-07 |
| DNAI4 | 79819 | -1.746316168 | 0.015504626 | 0.077171996 |
| CLMP | 79827 | -1.135542873 | 0.045487884 | 0.17279855 |
| CCDC102B | 79839 | -1.801688403 | 1.92E-04 | 0.002161181 |
| AGBL2 | 79841 | -1.240601689 | 7.01E-05 | 9.16E-04 |
| ZNF671 | 79891 | -1.187071128 | 7.15E-12 | 4.35E-10 |
| MRM1 | 79922 | -1.212476942 | 0.002434405 | 0.017950951 |
| SLC8B1 | 80024 | -1.878092807 | 1.64E-42 | 3.09E-39 |
| ZNF606 | 80095 | -1.624611992 | 1.44E-04 | 0.001707745 |
| ZFP2 | 80108 | -1.392060356 | 0.026901863 | 0.117699127 |
| UXS1 | 80146 | -1.198352093 | 1.11E-09 | 4.72E-08 |
| PGGHG | 80162 | -1.581578847 | 3.58E-16 | 4.53E-14 |
| HKDC1 | 80201 | -2.378156389 | 7.03E-04 | 0.006373786 |
| ALPK1 | 80216 | -3.142780687 | 1.66E-21 | 4.46E-19 |
| TASL | 80231 | -1.455308873 | 0.003334156 | 0.022936555 |
| EFHC2 | 80258 | -1.65775086 | 2.62E-05 | 3.90E-04 |
| TRIM45 | 80263 | -1.2229855 | 0.022553022 | 0.103108132 |
| CUBN | 8029 | -1.169213442 | 1.54E-04 | 0.001800887 |
| TSGA10 | 80705 | -1.104122797 | 2.06E-06 | 4.07E-05 |
| TSEN2 | 80746 | -1.235709607 | 1.39E-07 | 3.84E-06 |
| TMEM121 | 80757 | -2.573740523 | 0.027643843 | 0.120106998 |
| NDFIP1 | 80762 | -1.559871739 | 4.51E-20 | 1.04E-17 |
| TMEM177 | 80775 | -1.155686969 | 1.04E-04 | 0.001278731 |
| PRRT1 | 80863 | -2.020862526 | 1.32E-04 | 0.001582536 |
| AP3B2 | 8120 | -1.751367302 | 0.023748959 | 0.106943349 |
| CAMK4 | 814 | -1.475515511 | 1.18E-07 | 3.31E-06 |
| APOLD1 | 81575 | -1.197244169 | 0.041364419 | 0.161441436 |
| PLA2G12A | 81579 | -1.180955781 | 5.94E-10 | 2.61E-08 |
| AMN | 81693 | -3.466518899 | 0.026831586 | 0.117482777 |
| NUAK2 | 81788 | -1.072973359 | 8.43E-09 | 2.99E-07 |
| ZNF239 | 8187 | -1.124630377 | 3.30E-04 | 0.00343416 |
| SNN | 8303 | -2.302937556 | 2.21E-17 | 3.46E-15 |
| AXIN2 | 8313 | -2.089981542 | 7.68E-07 | 1.73E-05 |
| FZD6 | 8323 | -1.501039873 | 0.003752337 | 0.025265625 |
| DRC3 | 83450 | -1.124970018 | 0.005940584 | 0.036398442 |
| EPPK1 | 83481 | -3.641671572 | 2.66E-16 | 3.55E-14 |
| AKR1E2 | 83592 | -2.481523844 | 0.025508478 | 0.113123684 |
| NUDT12 | 83594 | -6.649826556 | 1.53E-08 | 5.15E-07 |
| RPS6KL1 | 83694 | -2.694610327 | 1.15E-06 | 2.48E-05 |
| SH3BGRL2 | 83699 | -2.128370203 | 0.014533123 | 0.073197634 |
| CASP6 | 839 | -1.208370432 | 2.35E-04 | 0.002573743 |
| IMMP2L | 83943 | -1.268262435 | 6.30E-11 | 3.29E-09 |
| REG4 | 83998 | -7.564515145 | 5.52E-43 | 1.34E-39 |
| OBSCN | 84033 | -1.889425576 | 1.40E-16 | 1.89E-14 |
| MYCBPAP | 84073 | -2.444918299 | 1.72E-04 | 0.001968362 |
| ZRANB3 | 84083 | -1.003604886 | 2.93E-06 | 5.60E-05 |
| USP44 | 84101 | -1.822579642 | 3.48E-10 | 1.60E-08 |
| SYDE2 | 84144 | -1.742450938 | 1.67E-05 | 2.65E-04 |
| ANXA9 | 8416 | -2.179115467 | 0.001023813 | 0.00873726 |
| CASP10 | 843 | -1.300402115 | 7.23E-07 | 1.65E-05 |
| ZNF397 | 84307 | -1.050470017 | 7.85E-08 | 2.30E-06 |
| ZBED3 | 84327 | -1.756765581 | 2.36E-05 | 3.57E-04 |
| ECRG4 | 84417 | -4.299988211 | 5.51E-10 | 2.47E-08 |
| MAML2 | 84441 | -1.946930095 | 2.13E-19 | 4.50E-17 |
| TPST1 | 8460 | -3.028706004 | 1.05E-13 | 9.32E-12 |
| ZNF594 | 84622 | -1.144687755 | 0.003685716 | 0.024879686 |
| ZNF347 | 84671 | -1.071149303 | 5.57E-05 | 7.48E-04 |
| BEX2 | 84707 | -1.449030281 | 3.99E-06 | 7.40E-05 |
| CBX2 | 84733 | -3.463157346 | 0.035742725 | 0.145193022 |
| GPAT3 | 84803 | -1.236815291 | 0.003638287 | 0.024662428 |
| PLPP7 | 84814 | -5.237199601 | 0.001567682 | 0.012508197 |
| ADTRP | 84830 | -3.212044323 | 0.005392874 | 0.033676609 |
| ZNF496 | 84838 | -1.874100644 | 5.68E-22 | 1.63E-19 |
| TMEM25 | 84866 | -1.369114951 | 7.87E-11 | 4.03E-09 |
| SLC7A3 | 84889 | -5.518998562 | 0.005226595 | 0.032893145 |
| UBASH3B | 84959 | -1.231931554 | 5.68E-10 | 2.54E-08 |
| CCDC183 | 84960 | -1.48128708 | 0.043770125 | 0.167964132 |
| TOX2 | 84969 | -5.149533759 | 8.33E-08 | 2.42E-06 |
| PAQR8 | 85315 | -1.394164569 | 0.015631708 | 0.077599117 |
| RHPN2 | 85415 | -4.329150681 | 0.001195542 | 0.009986255 |
| TSPYL5 | 85453 | -1.635406839 | 0.006502461 | 0.039077111 |
| DIXDC1 | 85458 | -1.5893776 | 9.24E-05 | 0.001160436 |
| ZNF518B | 85460 | -1.331027501 | 3.35E-09 | 1.30E-07 |
| KLF7 | 8609 | -3.064093526 | 1.64E-21 | 4.46E-19 |
| PLPP1 | 8611 | -1.575950996 | 5.66E-05 | 7.58E-04 |
| EIF4EBP3 | 8637 | -1.877906129 | 2.30E-08 | 7.50E-07 |
| DCHS1 | 8642 | -2.669591519 | 3.52E-09 | 1.36E-07 |
| PTCH2 | 8643 | -1.117569186 | 0.013749695 | 0.0698757 |
| IRS2 | 8660 | -1.359835508 | 1.34E-05 | 2.18E-04 |
| ACTN1 | 87 | -4.288668949 | 1.70E-97 | 2.22E-93 |
| TNK1 | 8711 | -2.295193213 | 4.41E-16 | 5.52E-14 |
| CBR3 | 874 | -2.679856772 | 1.03E-06 | 2.24E-05 |
| TNFRSF10D | 8793 | -2.901093215 | 8.60E-08 | 2.48E-06 |
| NR1I2 | 8856 | -3.162708858 | 4.85E-04 | 0.004700489 |
| FCGBP | 8857 | -2.106955632 | 0.001206851 | 0.010062874 |
| CACNA1I | 8911 | -2.094291602 | 0.016615111 | 0.081194781 |
| WASF1 | 8936 | -2.088132423 | 0.009935377 | 0.054660721 |
| UMODL1 | 89766 | -3.518985047 | 1.65E-04 | 0.001903327 |
| LMLN | 89782 | -1.751117705 | 2.00E-18 | 3.80E-16 |
| NAV2 | 89797 | -2.372288659 | 0.03585126 | 0.145477005 |
| CCNE1 | 898 | -1.058624821 | 0.043056977 | 0.165866883 |
| KLHL6 | 89857 | -2.266597272 | 1.18E-13 | 1.03E-11 |
| KALRN | 8997 | -1.538476204 | 0.006327865 | 0.038299879 |
| ZNF30 | 90075 | -1.051064475 | 3.46E-06 | 6.50E-05 |
| ZSWIM1 | 90204 | -1.845909997 | 2.53E-11 | 1.41E-09 |
| CH25H | 9023 | -2.500578451 | 1.01E-07 | 2.87E-06 |
| ZNF551 | 90233 | -1.147345482 | 1.69E-05 | 2.68E-04 |
| KLHL13 | 90293 | -2.004360172 | 0.031485901 | 0.132432217 |
| SEMA5A | 9037 | -1.688397649 | 0.035016561 | 0.143171118 |
| ZNF835 | 90485 | -2.228732206 | 2.57E-05 | 3.83E-04 |
| SLC7A6 | 9057 | -1.668981265 | 1.62E-11 | 9.33E-10 |
| ZNF439 | 90594 | -1.716128631 | 2.49E-10 | 1.18E-08 |
| PPP1R3E | 90673 | -1.616862807 | 3.65E-06 | 6.81E-05 |
| PRY | 9081 | -2.752479852 | 0.049113942 | 0.182952949 |
| SLC16A5 | 9121 | -1.540575355 | 3.58E-06 | 6.71E-05 |
| P2RX6 | 9127 | -2.896963727 | 4.33E-11 | 2.31E-09 |
| ZNF502 | 91392 | -1.284404254 | 0.009113597 | 0.05106934 |
| PCED1B | 91523 | -1.62668704 | 1.23E-14 | 1.27E-12 |
| PLXNA4 | 91584 | -4.667416471 | 2.15E-12 | 1.48E-10 |
| DEPDC7 | 91614 | -2.798119213 | 1.88E-06 | 3.78E-05 |
| CHMP7 | 91782 | -1.587006913 | 4.24E-13 | 3.32E-11 |
| TIMD4 | 91937 | -2.674914345 | 1.29E-07 | 3.58E-06 |
| ARRDC4 | 91947 | -2.093289805 | 1.54E-04 | 0.001805117 |
| ZNF300 | 91975 | -1.54134763 | 0.001527973 | 0.012249114 |
| OXNAD1 | 92106 | -1.804787059 | 1.04E-36 | 1.46E-33 |
| DSEL | 92126 | -2.353996283 | 2.36E-15 | 2.74E-13 |
| MTSS2 | 92154 | -1.690696574 | 0.008795928 | 0.049733874 |
| CRB3 | 92359 | -1.532643959 | 0.008829283 | 0.049805986 |
| PXYLP1 | 92370 | -1.060894858 | 2.84E-06 | 5.45E-05 |
| NOG | 9241 | -5.692603128 | 5.44E-36 | 6.68E-33 |
| FANK1 | 92565 | -3.161889526 | 0.00237149 | 0.017569606 |
| TMEM169 | 92691 | -1.480052961 | 0.00682262 | 0.040497787 |
| CD9 | 928 | -1.944996561 | 2.38E-04 | 0.00259893 |
| PEX11G | 92960 | -1.580423452 | 1.00E-05 | 1.67E-04 |
| ACVR2B | 93 | -1.078879965 | 1.34E-04 | 0.001599424 |
| SOCS6 | 9306 | -1.571096425 | 0.026704095 | 0.11701538 |
| ASIC3 | 9311 | -1.661472195 | 7.19E-04 | 0.006489629 |
| NREP | 9315 | -4.056234554 | 2.53E-14 | 2.45E-12 |
| SLC9A3R2 | 9351 | -1.208945874 | 0.048672803 | 0.181860639 |
| RAB33A | 9363 | -1.336742714 | 6.04E-08 | 1.81E-06 |
| ZFYVE9 | 9372 | -1.005160657 | 8.26E-05 | 0.001051356 |
| CD27 | 939 | -1.398970214 | 2.60E-07 | 6.60E-06 |
| CPA5 | 93979 | -6.676026904 | 9.10E-08 | 2.61E-06 |
| RECQL5 | 9400 | -1.069085013 | 1.95E-11 | 1.11E-09 |
| ZNF101 | 94039 | -1.169798513 | 2.19E-07 | 5.63E-06 |
| GNG8 | 94235 | -2.259423996 | 5.31E-04 | 0.005055421 |
| TNFSF8 | 944 | -1.739967989 | 3.95E-15 | 4.36E-13 |
| AKAP6 | 9472 | -3.528453518 | 1.08E-07 | 3.06E-06 |
| CEP41 | 95681 | -1.442402253 | 3.33E-16 | 4.26E-14 |
| RIN1 | 9610 | -1.424634823 | 0.00504581 | 0.031957427 |
| SH3PXD2A | 9644 | -1.496281097 | 3.44E-04 | 0.003551037 |
| ZNF516 | 9658 | -3.034008184 | 2.09E-12 | 1.45E-10 |
| GPRASP1 | 9737 | -2.041802852 | 2.32E-13 | 1.94E-11 |
| IFT140 | 9742 | -1.410476871 | 1.63E-06 | 3.33E-05 |
| ARHGAP32 | 9743 | -1.660749606 | 1.60E-04 | 0.001860189 |
| SNPH | 9751 | -1.016936646 | 0.001592749 | 0.012683168 |
| LZTS3 | 9762 | -1.792094536 | 1.49E-07 | 4.06E-06 |
| ARMCX2 | 9823 | -2.782792563 | 3.14E-15 | 3.59E-13 |
| ARHGEF11 | 9826 | -3.252379357 | 4.93E-14 | 4.63E-12 |
| TESPA1 | 9840 | -1.896086233 | 1.29E-17 | 2.18E-15 |
| CEP170 | 9859 | -1.123863524 | 6.24E-10 | 2.73E-08 |
| MAGI2 | 9863 | -1.390363896 | 6.01E-06 | 1.06E-04 |
| TBC1D4 | 9882 | -2.547535345 | 7.34E-24 | 2.49E-21 |
| SRGAP3 | 9901 | -2.268644897 | 1.12E-05 | 1.85E-04 |
| MRC2 | 9902 | -3.635726489 | 1.13E-09 | 4.76E-08 |
| KBTBD11 | 9920 | -2.803425943 | 4.02E-06 | 7.43E-05 |
| XYLB | 9942 | -1.48687519 | 2.67E-06 | 5.15E-05 |
| SLC12A6 | 9990 | -1.817424216 | 2.00E-19 | 4.34E-17 |
