# Supplementary material for: KLRG1-expressing CD8+ T cells are exhausted and polyfunctional in patients with chronic hepatitis B
Source: PLoS One. 2024 May 22;19(5):e0303945. doi: 10.1371/journal.pone.0303945 (PMC11111010; doi:10.1371/journal.pone.0303945)
Supplement: S4 Table — (DOCX) [file pone.0303945.s009.docx]

**KLRG1-expressing CD8+ T cells are exhausted and polyfunctional in patients with chronic hepatitis B.**

S4 Table. Specific primers.

| HBV DNA F | CCTAGTAGTCAGTTATGTCAAC |
| --- | --- |
| HBV DNA R | TCTATAAGCTGGAGGAGTGCGA |
