# Supplementary material for: KLRG1-expressing CD8+ T cells are exhausted and polyfunctional in patients with chronic hepatitis B
Source: PLoS One. 2024 May 22;19(5):e0303945. doi: 10.1371/journal.pone.0303945 (PMC11111010; doi:10.1371/journal.pone.0303945)
Supplement: S1 File — (DOCX) [file pone.0303945.s010.docx]

**KLRG1-expressing CD8+ T cells are exhausted and polyfunctional in patients with chronic hepatitis B.**

# Supplementary Materials and Methods

**Data acquisition and laboratory evaluation**

The basic clinical and laboratory data of patients and HCs included in the study were summarized in Table 1. Age, sex, and medication history were confirmed when they were first included in the study. Major laboratory tests (HBV DNA, HBsAg, HBeAg, and anti-HBe) were performed in the central laboratory. A highly sensitive quantification of serum HBV DNA was performed using Abbott kit (Abbott) with a lower and upper limits of detection of 10 IU/ml and 1.0 × 10^9^ IU/ml, respectively. HBsAg, HBeAg, and anti‐HBe were tested using Abbott kits (Abbott) according to the instructions, all indexes are measured by absolute quantitative method except for anti-HBeAg. ALT and AST were collected from the laboratory department of our hospital.

**Cell isolation**

Five milliliters of heparinized peripheral venous blood was obtained from patients with cHBV infection or HCs, followed by isolation of peripheral blood mononuclear cells (PBMCs) using Histopaque-1077 (Sigma-Oldridge, USA). According to the manufacturer's instructions, whole blood was gently added to a tube preloaded with 3-5 ml separation solution to avoid inconspicuous stratification of the liquid surface. After centrifugation at 400g, 4°C for 30 min, the PBMC layer in the middle white mist was carefully aspirated and washed once with 5 ml of pre-cooled PBS. PBMCs were then inoculated in FACS buffer in the wells of the plates (PBS containing 2% FCS [Invitrogen]) for subsequent experiments.

**ELISpot**

ELISpot assays for the detection of IFNγ–producing cells were performed on in vitro-expanded T cell lines using 15-mer peptides pooled of HBcAg (Genotype B). T cell lines were incubated overnight at 37°C with pools of HBV peptides (10 μg/ml). IFNγ ELISpot assays (Millipore) were performed as described previously (1).

**Apoptosis and akt(Ser473) phosflow assay for KLRG1+ T cell**

2×10^6^ PBMCs were stimulated by anti-CD3 (BD Bioscience,USA) overnight in addition of 5ug/ml sE-cadherin (ABclonal,USA). After that, cells were washed and resuspended in FACS buffer(PBS containing 2% FBS), and stained with PerCP/Cyanine 5.5 anti-human CD3, APC/Cyanine7 anti-human CD8, PE/Cyanine7 anti-human KLRG1 and FITC anti-human Annexin V according to the instructions of the reagent manufacturer. Isolated PBMC were irritated by sE-cadherin in the presence of anti-CD3 as experimental wells, while the adding of PBS was used as control wells as described previously (2), incubated for 2 hours at 37℃ in 5% CO2. Cells were fixed with Cytofix/Cytoperm™Fixation/Permeabilization Kit and pretreated with Phosflow III solution (BD Bioscience, USA), then stained with anti-human CD8, anti-human KLRG1 and anti-human Akt (Ser473), finally analyzed using the CytoFLEX flow cytometer (Beckman Coulter, Fullerton, CA, USA). All flow cytometry data were analyzed using FlowJo v10.8.1.

**In vitro HBV-specific IFN-γ detection assay**

The cryopreserved cells were thawed and rested overnight, and then inoculated in 96-well plates at an inoculation rate of 2×10^5 wells/well. 1ug/ml of sE-cadherin and 10ug/ml of HBc18-27 (FLPSDFFPSV, Sangon Biotech) were added to the experimental wells simultaneously, and the wells with HBc18-27 alone was considered as the control. Cytokine secretion from all wells was blocked with BFA/Monensin Mixture for the next 5h. Cells were stained with CD3, CD8 monoclonal antibody, and KLRG1 monoclonal antibody, after fixation and permeabilization, further stained with anti-IFN-γ monoclonal antibody for subsequent analysis.

**siRNA Transfections**

Fifty pmol of targeting small interfering or non-targeting siRNAs were transfected into HepG2.2.15 cell lines planted into 6-well plate using siRNA-mate (Genephama, Shanghai, China) according to the manufacturer’s protocol. The sequence used for siRNA targeting E-cadherin was: 5′-CAGACAAAGACCAGGACUA-3′.

**Western blotting (WB)**

Western blot analysis was performed as previously described (3). In brief, expanded HBV-specific CD8+ T cells were co-cultured with HepG2.2.15 at a ratio of 5:1 for 72h, in the presence or absence of sE-cadherin (ABclonal, USA). Removal of supernatant and suspended PBMCs and HepG2.2.15 were washed 3 times with pre-cooled phosphate-buffered saline, proteins of remained HepG2.2.15 were extracted by lysis buffer containing protease inhibitors (keygentec, China) according to the manufacturer’s instructions. The concentration of protein was quantified via bicinchoninic acid (BCA) protein assay (Beyotime, China). Equal amounts of protein samples were loaded and separated by sodium dodecyl sulfate-polyacrylamide gel electrophoresis (8%-12%, depending on the molecular weight of the target protein). The proteins were then transferred to polyvinylidene fluoride membranes (PVDF) using WB transfer buffer. The membranses were then blocked with 5% non-fat dry milk in TBST for 1h at 37℃and incubated with HBV core antigen primary antibody (gifted by Professor Xuefei Cai) at 4 ℃ overnight and then with goat polyclonal secondary antibody to mouse IgG-H&L (HRP) (Boster, China) for 1h. Finally, level of protein was detected with enhanced chemiluminescence (ECL) kit (NCM Biotech, China) and visualized by using ChemiDoc Imaging System (BIO-RAD, USA).

**HBV DNA extraction and quantitative PCR**

Extraction of HBV-DNA was performed as previously described in detail (4). Briefly, cells were incubated in 500 ul of lysis buffer (10 mM Tris-HCl pH 8.0, 1 mM EDTA, 1% NP-40, 2% sucrose) at 37°C for 15 min and then centrifuged at 16,000 for 5 min to remove nuclei. β-actin in cytoplasmic cell lysate were detected by western blot. The remaining cell lysates were digested (40 IU/mL DNase I and 10 mM MgCl2) for 4h to remove free cell nucleic acids. HBV DNA was acquired by precipitation with 5% PEG8000 and incubation with proteinase K. After purification by phenol/chloroform (1:1), HBV DNA was precipitated with ethanol and finally dissolved in TE buffer. The extracted HBV DNA was subjected to absolute quantitative PCR using TB Green premix Ex Taq (Takara Bio) with specific primers listed in the supplementary materials.

**Transcriptomic RNA sequencing (RNA-seq)**

Sorting of target cells based on KLRG1 expression was performed on 5 pairs of strictly matched CHB patients, and the total RNA was extracted using RNeasy Micro kit (QIAGEN, GER) according to the manual instruction. Next, the RNeasy MinElute spin column which contained RNA was placed in a new 2 mL collection tube, centrifuged with lid opened at full speed for 5 min to dry the membrane, followed by transferred to a new 1.5mL tube with 14 µL RNase-free water. Finally, tubes were centrifuged for 1 min at full speed to elute the RNA. Subsequently, a Nano Drop and Agilent 2100 bionanalyzer (Thermo Fisher Scientific, MA, USA) was used to qualify and quantify total RNA. Following acquisition of high quality RNA, the amplification product, namely cDNA, is generated by PCR. Fragmented cDNA were heated, denatured, and circularized by splint oligo sequence. The single strand circle DNA was formatted as the final library and amplified with phi29 (Thermo Fisher Scientific, MA, USA) to make DNA nanoballs that were loaded into patterned nanoarrays. Paired end 100-base reads were generated on a BGISEQ200 platform (BGI, Shenzhen, China) and data were analyzed with Dr. Tom.

**Enzyme-linked immunosorbent assay (ELISA)**

Serum levels of sE-cadherin were measured by human sE-cadherin ELISA kit (Shanghai Jianglai Industrial Limited by Share Ltd., Shanghai, China). Serum concentrations of KLRG1 were detected by human KLRG1 ELISA kit (Shanghai Jianglai Industrial Limited by Share Ltd., Shanghai, China). Briefly, plasma samples were added to 96-well plates, and then the detection solutions and wash buffers were dispensed into each well in accordance with the manufacturer’s instructions. The absorbance values of each well were read at 450 nm using a Thermo Fisher Scientific Varioskan Flash multimode reader.

# References

1. Tan AT, Loggi E, Boni C, Chia A, Gehring AJ, Sastry KS, et al. Host ethnicity and virus genotype shape the hepatitis B virus-specific T-cell repertoire. J Virol. 2008;82(22):10986-97.

2. Henson SM, Franzese O, Macaulay R, Libri V, Azevedo RI, Kiani-Alikhan S, et al. KLRG1 signaling induces defective Akt (ser473) phosphorylation and proliferative dysfunction of highly differentiated CD8+ T cells. Blood. 2009;113(26):6619-28.

3. Zhang X, Wei M, Fan J, Yan W, Zha X, Song H, et al. Ischemia-induced upregulation of autophagy preludes dysfunctional lysosomal storage and associated synaptic impairments in neurons. Autophagy. 2021;17(6):1519-42.

4. Cheng ST, Hu JL, Ren JH, Yu HB, Zhong S, Wai Wong VK, et al. Dicoumarol, an NQO1 inhibitor, blocks cccDNA transcription by promoting degradation of HBx. J Hepatol. 2021;74(3):522-34.
